# Efficient synthesis of complex supramolecular topologies via coordination angle modulation

**DOI:** 10.1093/nsr/nwag432

**Published:** 2026-07-13

**Authors:** Li-Long Dang, Xiao-Qian Wan, Jian-Xin Yang, Mei-Jia Chang, Francisco Aznarez, Ying Zhao, Feng Bai, Lu-Fang Ma

**Affiliations:** Henan Province Function-Oriented Porous Materials Key Laboratory, College of Chemistry and Chemical Engineering, Luoyang Normal University, Luoyang 471934, China; School of Nanoscience and Materials Engineering, Henan University, Kaifeng 475004, China; Henan Province Function-Oriented Porous Materials Key Laboratory, College of Chemistry and Chemical Engineering, Luoyang Normal University, Luoyang 471934, China; School of Nanoscience and Materials Engineering, Henan University, Kaifeng 475004, China; Henan Province Function-Oriented Porous Materials Key Laboratory, College of Chemistry and Chemical Engineering, Luoyang Normal University, Luoyang 471934, China; School of Environmental Engineering and Chemistry, Luoyang Institute of Science and Technology, Luoyang 471023, China; Henan Province Function-Oriented Porous Materials Key Laboratory, College of Chemistry and Chemical Engineering, Luoyang Normal University, Luoyang 471934, China; Henan Province Function-Oriented Porous Materials Key Laboratory, College of Chemistry and Chemical Engineering, Luoyang Normal University, Luoyang 471934, China; School of Nanoscience and Materials Engineering, Henan University, Kaifeng 475004, China; Henan Province Function-Oriented Porous Materials Key Laboratory, College of Chemistry and Chemical Engineering, Luoyang Normal University, Luoyang 471934, China

**Keywords:** catenane, molecular knot, folded assembly, *π*···*π* stacking interactions, photothermal conversion

## Abstract

Despite the appeal of mechanically interlocked supramolecular systems, precise control over their assembly into complicated topologies is still a central challenge. To address this, we employed a method centered on regulating the coordination angle. By designing two ligands (**L1** and **L2**) with differing carbazole-pyridine connection angles and combining them with tailored building units **E1**(OTf)_2_ and **E2**(OTf)_2_, we successfully directed the synthesis of four unique architectures: a molecular-tweezer-like compound, a snowflake-shaped folded assembly, an 8_18_ knot, and a closed three-link chain. Single-crystal X-ray diffraction analysis provided unambiguous structural elucidation, not only confirming the three-dimensional geometries but also revealing that the ligand angle dictates the outcome of assembly, steering it toward interlocked or folded structures. The formation and purity of these constructs in solution were verified by nuclear magnetic resonance spectroscopy and electrospray ionization time-of-flight mass spectrometry. Furthermore, photothermal conversion experiments demonstrated that compound **4**(OTf)_12_ exhibits favorable photothermal performance, with a relatively high photothermal conversion efficiency as well as excellent cycling stability. This observation is also supported by electron paramagnetic resonance measurement data.

## Teaser

Coordination-angle regulation enables the controlled self-assembly of four distinct topological architectures, including a molecular-tweezer-like compound, a snowflake-shaped folded assembly, an 8_18_ knot, and a closed three-link chain.

## INTRODUCTION

In nature, the diverse functions of proteins and DNA are often dictated by their distinct folding and interlocking patterns [1–5]. Inspired by this, we have synthesized supramolecular complexes with programmable architectures to explore the structure–function relationship. Even minor structural variations can have a profound functional impact, a central question that drives research in this field. Given its significant potential in life sciences, this area merits in-depth exploration [6–10].

In recent years, diverse construction strategies have enabled the synthesis of a wide array of intricate supramolecular complexes, including molecular knots [11–14], ravels [15–18], catenanes [19–23], and cages [24–28]. The successful preparation of these compounds has not only accelerated the development of topological chemistry but also facilitated detailed investigations into structural interconversions among complex architectures. Notable examples include a silver-ion-promoted conversion between an 8_18_ knot and a Borromean ring [29], as well as the transformation between a 4_1_ knot and a double trefoil knot, driven by the coordination of metal ions to amide oxygen atoms [30,31]. Intriguingly, solvent and concentration effects have also been employed to convert complicated topologies into their corresponding macrocyclic compounds [32,33].

Despite these advances, the fundamental mechanisms governing the synthesis of high-order topological compounds remain poorly understood. Consequently, developing reliable methods to construct even more complex topologies, such as 8_18_ knots or closed [3]catenanes, remains a significant challenge. Furthermore, there are very few reports on directing the mechanical interlocking of multinuclear compounds or forming multiply folded macrocycles through ligand configuration control. Advancing this area could hold substantial research significance, particularly for the field of life sciences.

Based on our systematic investigations and the collective findings accumulated in our group over the years, several empirical trends have emerged. In general, when employing a two-component assembly strategy to synthesize mechanical interlocking structures based on half-sandwich rhodium/iridium units, bidentate ligands with small coordination angles tend to afford metallamacrocycles [34] or molecular-tweezer-like compounds [35,36], owing to the steric hindrance imposed by the ligand framework. In contrast, linear rigid bidentate ligands frequently lead to the formation of linear [3]/[4]catenanes [37–39] and borromean rings [40–42]. Consequently, the realization of both cyclic and closed topologies—such as the 8_18_ knot and the closed three-link chain—demands precise ligand angle control. Specifically, the ligands need to adopt a subtly curved conformation with a gentle angle to facilitate the formation of initial boat-shaped metallamacrocycles, which can meet the requirements for finally forming cyclic and closed topologies.

For this study, we synthesized two highly symmetrical carbazole-based bidentate pyridyl ligands, **L1** and **L2**, both featuring extensive conjugate centers but differing in their coordination angles. We hypothesized that combining these with suitable half-sandwich rhodium building units—which possess specific conjugate planes—would steer the assembly toward distinct topological outcomes: high-order interlocked structures or folded, non-interlocked assemblies. To test this, we also prepared two such rhodium-based units, **E1**(OTf)₂ and **E2**(OTf)₂, which differ in their conjugate planes and longitudinal dimensions. Building on our previous work, where the combination of **L1** and **E1**(OTf)_2_ yielded a molecular-tweezer-like folded assembly, **1**(OTf)_8_, we now report that reacting ligand **L1** with the larger **E2**(OTf)_2_ unit directly produces an unprecedented dodecanuclear folded cage, **2**(OTf)_12_. Furthermore, when ligand **L2** was employed, its combination with **E1**(OTf)_2_ and **E2**(OTf)_2_ resulted in the formation of an 8_18_ knot, **3**(OTf)_16_, and a closed three-link chain, **4**(OTf)_12_, respectively. All the new complexes—**2**(OTf)_12_, **3**(OTf)_16_, and **4**(OTf)_12_—were isolated in high yields and thoroughly characterized by single-crystal X-ray diffraction (SC-XRD), nuclear magnetic resonance (NMR) spectroscopy, and electrospray ionization time-of-flight mass spectrometry (ESI-TOF-MS). We attribute the divergent formation of these topologies primarily to the distinct coordination angles of ligands **L1** and **L2**, coupled with the specific stacking interactions between their conjugate centers and those of the building units.

## RESULTS AND DISCUSSION

The combination of building blocks **E1**(OTf)_2_ or **E2**(OTf)_2_ with ligands **L1** or **L2** led to the formation of four distinct supramolecular complexes in excellent yields: a molecular-tweezer-like compound **1**(OTf)_8_ (86.9%), a snowflake-shaped folded assembly **2**(OTf)_12_ (88.7%), an 8_18_ knot **3**(OTf)_16_ (85.2%), and a closed three-link chain **4**(OTf)_12_ (89.4%). Single crystals of these compounds, suitable for X-ray diffraction analysis, were subsequently obtained by slowly diffusing isopropyl ether into their concentrated methanolic solutions (Scheme 1).

**Scheme 1.**
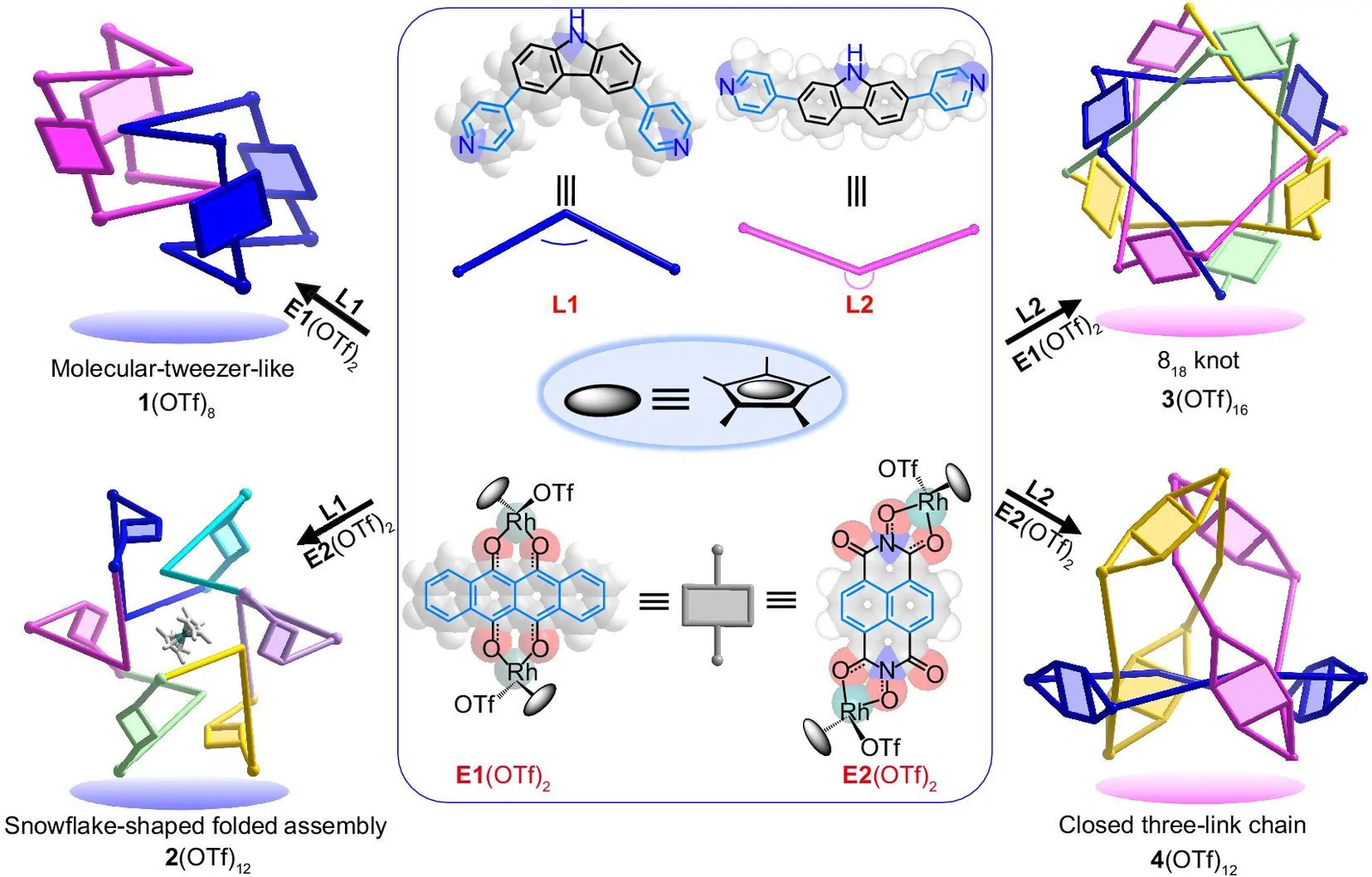
Synthesis of molecular-tweezer-like compound **1**(OTf)_8_, snowflake-shaped folded assembly **2**(OTf)_12_, 8_18_ knot **3**(OTf)_16_, and closed three-link chain **4**(OTf)_12_.

The structure of compound **1**(OTf)_8_ has been reported by our research team [43]. It was synthesized by connecting the half-sandwich building unit **E1**(OTf)_2_ with the pyridine-based ligand precursor **L1**. Notably, its architecture incorporates two bent tetranuclear metallamacrocycles embedded within an interdigitated macrocyclic topology via *π*···*π* stacking interactions between carbazolyl groups on adjacent ligands. The significant Rh···Rh separation within these units (∼8.44 Å; Fig. S3) facilitated this interpenetration by providing ample spatial accommodation. Consequently, two identical tetranuclear macrocycles adopt a clamped conformation, forming a stable octanuclear assembly (Fig. 1a and b). Distinct colors and simplified diagrams were employed to visualize the spatial relationships between components in **1**(OTf)_8_ (Fig. 1c and d). Slightly twisted *π*···*π* stacking was observed, characterized by interlayer distances of 3.70, 3.78, and 3.67 Å. To probe how building block length affects the resultant topology, we utilized a longer NDI-based dinuclear unit **E2**(OTf)_2_ (featuring an extended conjugated plane) combined with **L1**. This yielded the unprecedented snowflake-shaped folded assembly **2**(OTf)_12_, whose structure was confirmed by SC-XRD. Crystallizing in the triclinic *P*1 space group, **2**(OTf)_12_ exhibits alternating connectivity between organic ligands and metallobuilding units (Fig. 1e). We propose that its formation stems from *π*···*π* interactions between ligand carbazolyl groups and NDI moieties of the building unit. Furthermore, the angular geometry of **L1** inhibits interlocking in **2**(OTf)_12_, instead directing self-assembly into this unique supramolecular framework—highlighting ligand design’s crucial role in controlling structural topology. The central cavity of **2**(OTf)_12_ principle enables guest encapsulation within its stable architecture housing twelve Rh atoms (Fig. 1f and g). Following reduction via Reidemeister moves [44,45], **2**(OTf)_12_ was identified as a large fold-type metallamacrocycle comprising over 400 non-hydrogen atoms (Fig. 1h).

**Figure 1.**
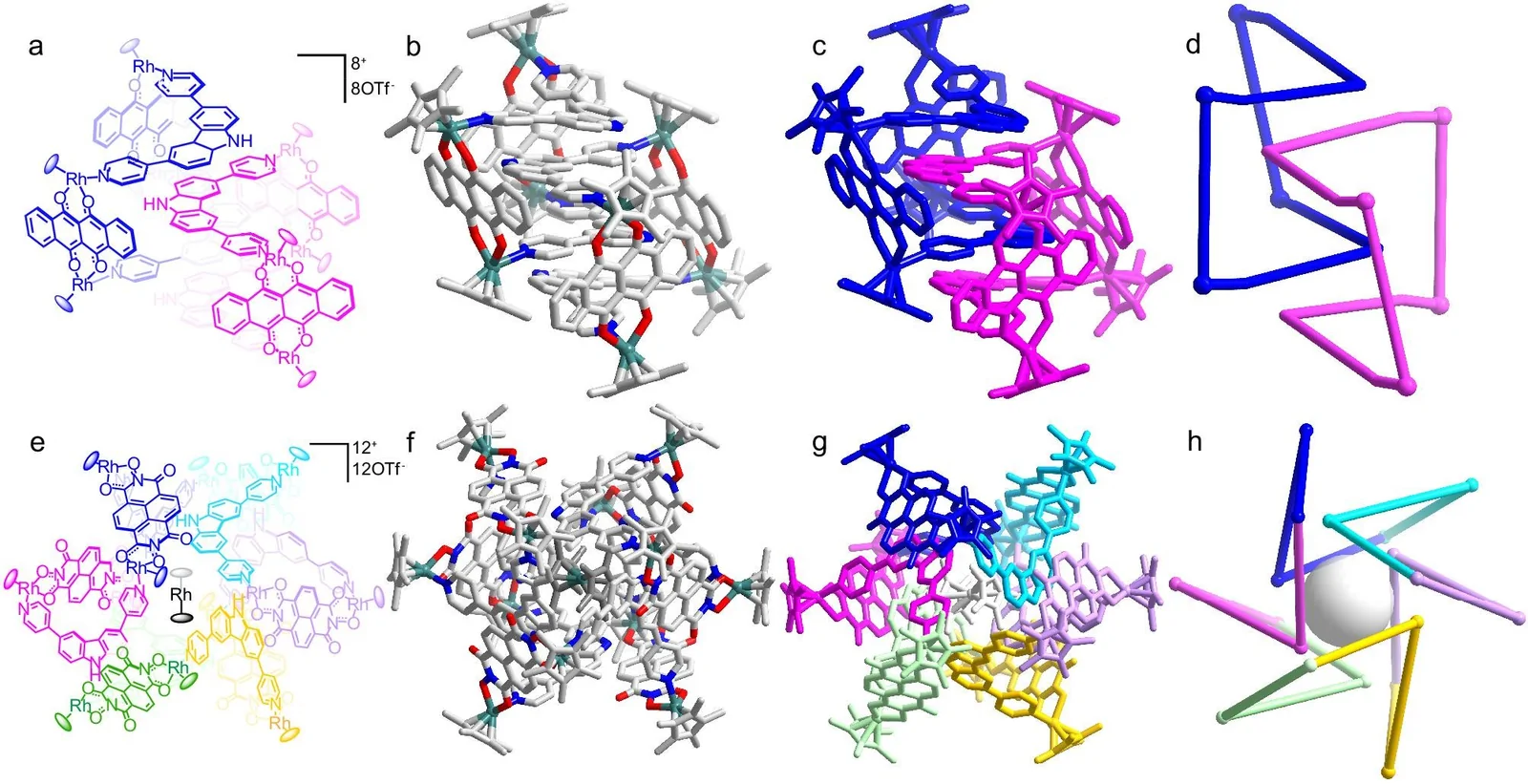
(a) Chemical structure, (b) molecular structure, (c) molecular structure with different colors, and (d) simplified topological representation of molecular-tweezer-like compound **1**(OTf)_8_. (e) Chemical structure, (f) molecular structure, (g) molecular structure with different colors, and (h) simplified topological representation of snowflake-shaped folded assembly **2**(OTf)_12_. The central larger sphere in compound **2**(OTf)_12_ denotes the Cp* group attached to Rh. The larger nodes represent the Rh metal centers in the complex, the broken line segments denote the bridging ligands, and the straight line segments stand for the building units. Most hydrogen atoms, anions, and solvent molecules have been omitted for clarity, and the color scheme is as follows: N (blue), O (red), C (gray), and Rh (sea green).

Figure S4a and b demonstrate compound **2**(OTf)_12_, with an outer edge diameter of ∼26.04 Å and a vertical height of 28.60 Å. In order to analyze the *π*···*π* stacking interactions within the molecule intuitively, the conjugate surfaces of the ligands and the building units were distinguished by different colors. As depicted in Fig. S4c, the measured distances between the ligands and the building units were 3.43 and 3.79 Å, respectively, clearly reflecting the *π*···*π* stacking interactions of each component.

The isolation of this rare contracted supramolecular metallamacrocycle represented a significant advancement in the exploration of supramolecular chemistry, offering new insights into the structural complexity and functional versatility of supramolecular compounds. This breakthrough not only expands the type of synthetic metallamacrocycle architectures but also develops our understanding of non-covalent interactions and molecular self-assembly. A structural comparison between compounds **1**(OTf)_8_ and **2**(OTf)_12_ revealed that both the appropriate size and conjugation effect of half-sandwich building units induced the assembly of multinuclear metallamacrocycle compounds, thereby suggesting that the key factor most likely resides in the geometry of the ligand. This insight provides a valuable foundation for advancing the construction of increasingly intricate entangled architectures.

We next acquired ^1^H NMR, ^1^H-^1^H correlation spectroscopy (COSY), and ^1^H diffusion-ordered spectroscopy (DOSY) spectra of **2**(OTf)_12_ in solution (Fig. 2a–c). Splitting patterns were analyzed and integrals determined for all signals. Assignment of all resonances was unambiguously achieved by using the ^1^H-^1^H COSY spectrum (Fig. S11). The signals at 8.28 and 7.82 ppm were confirmed, which belong to the pyridine protons of the ligand precursor **L1**. Furthermore, the signals corresponding to the carbazole protons of **L1** appeared at 7.88, 7.67, and 7.32 ppm, and the signal at 5.71 ppm was assigned to the protons of building unit **E2**(OTf)_2_. In addition, the resonances belonging to the protons of the Cp* groups appeared as a singlet at 1.72 ppm in the ^1^H NMR spectrum. Note that the presence of a single, sharp set of signals in the ^1^H NMR spectrum indicated a high degree of molecular symmetry. Compound **2**(OTf)_12_ exhibited a single diffusion coefficient (*D* = 2.54 × 10^−6^ cm^2^ s^−1^), thereby confirming the presence of only one species in solution. The hydrodynamic radius of **2**(OTf)_12_ (14.82 Å), derived from the ^1^H DOSY NMR diffusion constant, was in excellent agreement with its solid-state measurement (14.99 Å), which demonstrated structural consistency between the solution and solid phases (Fig. S18).

**Figure 2.**
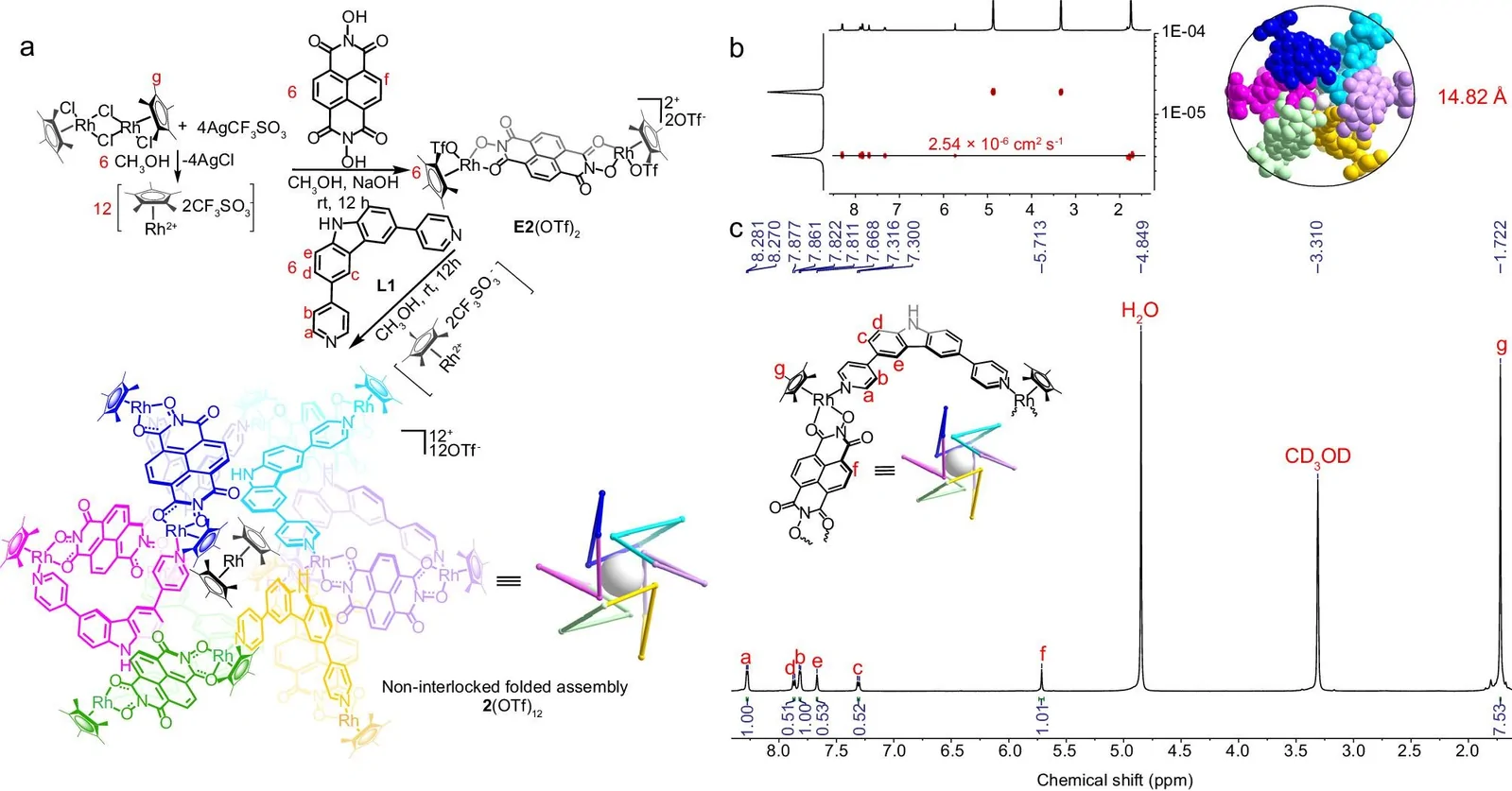
(a) Synthesis of snowflake-shaped folded assembly **2**(OTf)_12_. (b) ^1^H DOSY NMR spectrum (500 MHz, CD_3_OD, 298 K) (2.54 × 10^−6^ cm^2^ s^−1^), and (c) ^1^H NMR spectrum (500 MHz, CD_3_OD, 298 K) of snowflake-shaped folded assembly **2**(OTf)_12_ (24.5 mM, with respect to Cp*). The larger nodes represent the Rh metal centers in the complex, the angled line segments denote the bridging ligands, and the straight line segments stand for the building units.

It was observed that the relatively small bite angle and remarkable conjugation in the ligand precursor **L1** facilitated the formation of the molecular tweezer-like compound **1**(OTf)_8_ and the snowflake-shaped folded assembly **2**(OTf)_12_. In an attempt to increase the complexity of the resulting interlocking supramolecular structures, a new ligand precursor **L2** with an expanded angle was synthesized using a Suzuki–Miyaura cross-coupling reaction (Figs S1, S2, and S7–S10). Then, ligand precursor **L2** was reacted with building units **E1**(OTf)_2_ or **E2**(OTf)_2_ at ambient temperature for 12 h to produce compounds **3**(OTf)_16_ or **4**(OTf)_12_, respectively (Fig. 4a). Single crystals of the resulting self-assemblies suitable for X-ray diffraction analysis were obtained by slow diffusion of isopropyl ether into the corresponding methanolic solutions. SC-XRD analysis confirmed the intricately tangled architecture of compound **3**(OTf)_16_, belonging to the *P*2 space group. As shown in Fig. 3a and b, each building unit **E1**(OTf)_2_ and ligand **L2** were coordinatively bridged by a Rh metal center in the supramolecular assembly, thereby forming an interlocked supramolecular framework. Theoretical arithmetic revealed the presence of *π*···*π* stacking interactions between the tetraphenyl groups of **E1**(OTf)_2_ and the carbazolyl group of ligand **L2**, which enhanced the structural stability (Fig. S39). Additionally, the measured distances between ligand **L2** and the building unit **E1**(OTf)_2_ were 3.56 and 3.64 Å. Notably, the hydrogen bonds between the carbazolyl group and the pyridyl group of **E1**(OTf)_2_ were separated by distances of 2.50 and 2.83 Å, respectively (Fig. S5). The synergistic interaction of coordination-driven self-assembly and *π*···*π* stacking interactions led to the formation of a highly stable hexadecanuclear supramolecular knot. Moreover, the compound **3**(OTf)_16_ exhibited an outer diameter of 28.37 Å and a vertical height of 22.90 Å. The molecular structure was represented in five distinct colors (Fig. 3c) and simplified diagram (Fig. 3d) to provide an intuitive understanding of the spatial arrangement and interactions between moieties in this unique 8_18_ knot.

**Figure 3.**
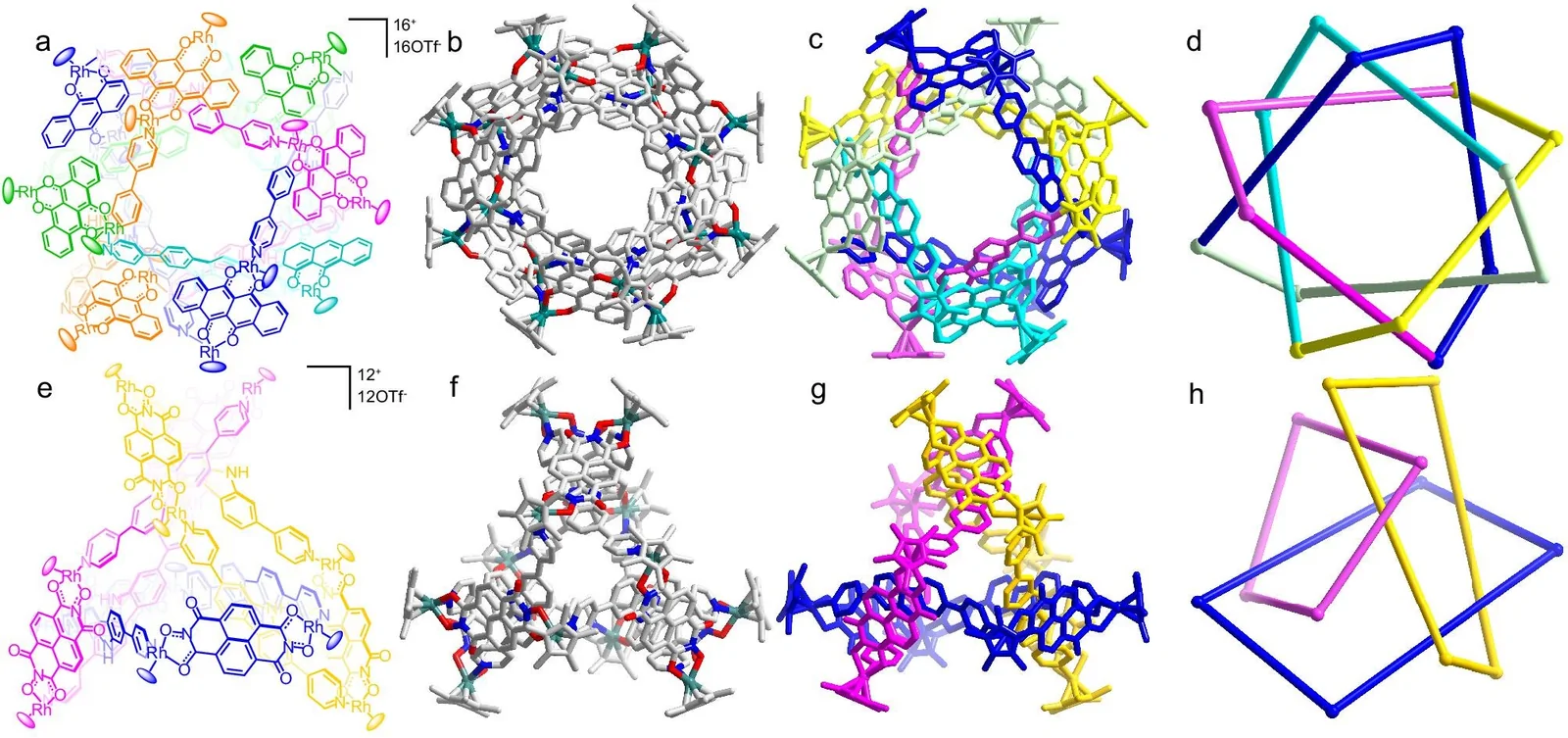
(a) Chemical structure, (b) molecular structure, (c) molecular structure with different colors, and (d) simplified topological representation of 8_18_ knot **3**(OTf)_16_. (e) Chemical structure, (f) molecular structure, (g) molecular structure with different colors, and (h) simplified topological representation of closed three-link chain **4**(OTf)_12_ (${\mathrm{6}}_{\mathrm{1}}^{\mathrm{3}}$ link). The larger nodes represent the Rh metal centers in the complex, the long line segments denote the bridging ligands, and the short line segments stand for the building units. Most hydrogen atoms, anions, and solvent molecules have been omitted for clarity, and the color scheme is as follows: N (blue), O (red), C (gray), and Rh (sea green).

The behavior of compound **3**(OTf)_16_ in solution was also recorded via ^1^H NMR, ^1^H-^1^H COSY NMR, and ^1^H DOSY NMR spectra. In the ^1^H NMR spectrum of **3**(OTf)_16_, clearly discernible signals and multiple coupling interactions were observed (Fig. S12). While the ^1^H NMR spectrum of **3**(OTf)_16_ exhibited significant signal overlap, complicating spectral analysis, ^1^H DOSY NMR spectroscopy confirmed the presence of a single species in solution (Fig. 4b). Furthermore, ^1^H-^1^H COSY NMR spectroscopy correlations enabled the assignment of coupling interactions to their respective proton signals (Figs S13 and S14). Two distinct singlets appeared at 1.704 and 1.697 ppm in the ^1^H NMR spectrum of **3**(OTf)_16_, which were assigned to the protons of the Cp* groups. Consequently, the aromatic region of the ^1^H NMR spectrum was expected to show two different sets of signals. The four signals that appeared at 8.86, 8.78, 8.50, and 8.25 ppm were assigned to the pyridine protons of the ligand **L2**. Furthermore, the resonances observed at 8.42, 8.38, 8.18, 8.09, 7.87, and 7.79 ppm were assigned to the carbazole protons of ligand **L2** and the other two signals detected at 8.67 and 8.26 ppm were attributed to the quaterphenylene protons of **E1**(OTf)_2_ (peak assignments are provided in Section 2.5 of the Supplementary Data). The hydrodynamic radius of **3**(OTf)_16_ (14.79 Å) was calculated from the ^1^H DOSY spectrum, and the result was compared with that obtained from the molecular structure (15.5 Å) (Fig. S19). ESI-TOF-MS analysis substantiated the structural assignment, revealing an intense peak at *m/z* = 2065.2412 [**3**(OTf)_16_–5OTf^−^]^5+^ that aligns with the theoretical distribution proposed for 8_18_ Knot **3**(OTf)_16_ (Fig. 4c, d and Figs S21–S23).

**Figure 4.**
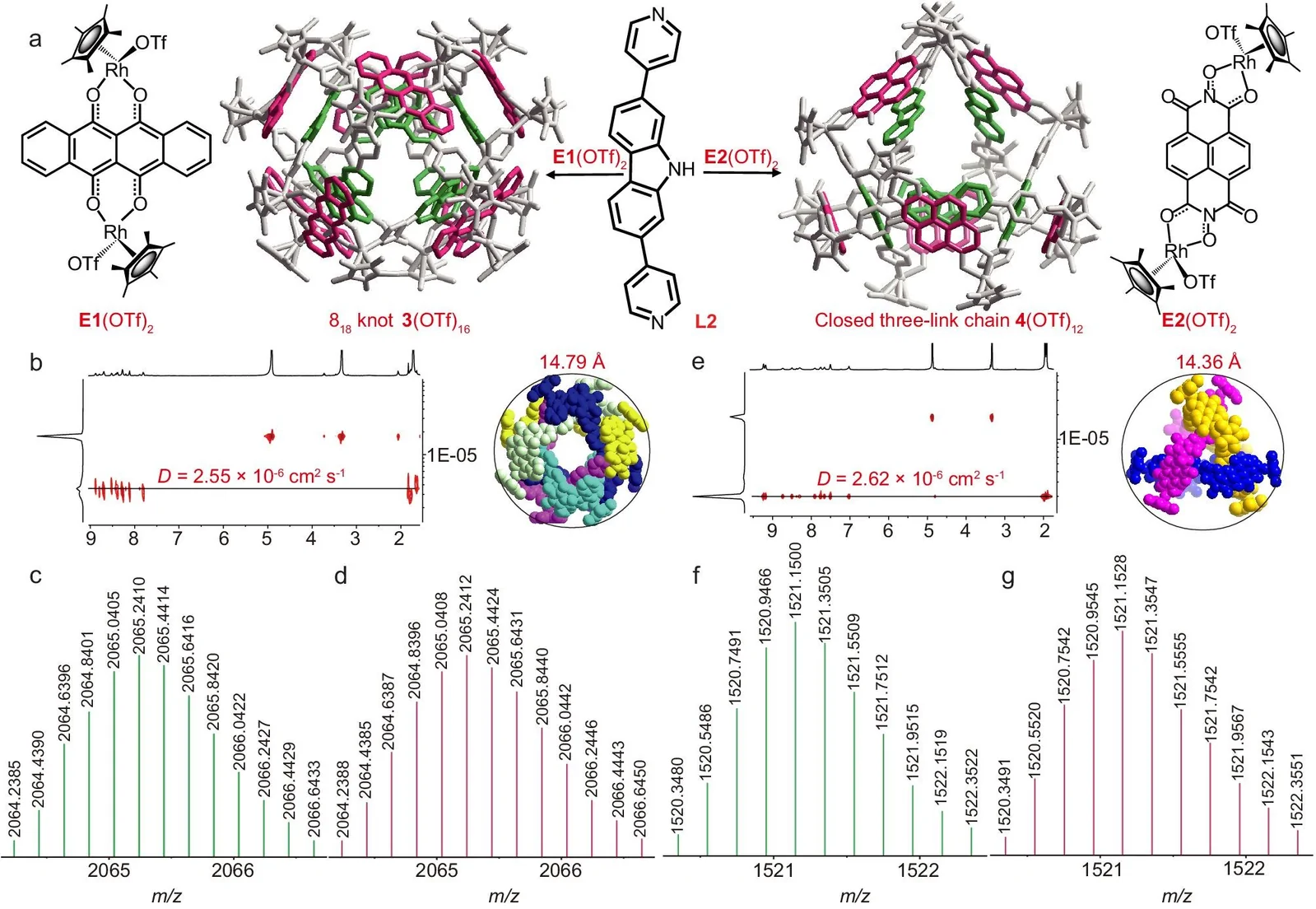
(a) Schematic diagram for the synthesis of 8_18_ knot **3**(OTf)_16_ and closed three-link chain **4**(OTf)_12_. (b) ^1^H DOSY NMR spectrum (500 MHz, CD_3_OD, 298 K, *c* = 14.5 mM with respect to Cp*, *D* = 2.55 × 10^−6^ cm^2^ s^−1^) of 8_18_ knot **3**(OTf)_16_. (c) Calculated value and (d) measured value of ESI-TOF-MS spectra of 8_18_ knot **3**(OTf)_16_ ([**3**(OTf)_16_–5OTf^−^]^5+^). (e) ^1^H DOSY NMR spectrum (500 MHz, CD_3_OD, 298 K, *c* = 13.0 mM with respect to Cp*, *D* = 2.62 × 10^−6^ cm^2^ s^−1^) of closed three-link chain **4**(OTf)_12_. (f) Calculated value and (g) measured value of ESI-TOF-MS spectra of closed three-link chain **4**(OTf)_12_ ([**4**(OTf)_12_–5OTf^−^]^5^^+^).

Moreover, single crystals suitable for SC-XRD analysis were obtained through slow vapor diffusion of diethyl ether into a solution of **4**(OTf)_12_ in methanol at ambient temperature (Fig. 3e and f). The elucidated molecular structure was represented in three different colors (Fig. 3g) and simplified diagram (Fig. 3h) to provide an intuitive understanding of the spatial arrangement and intercomponent relationships. The cyclic architecture of **4**(OTf)_12_ displays a rare interlocked topology in which each ring is interlinked with adjacent ones, corresponding to a ${\mathrm{6}}_{\mathrm{1}}^{\mathrm{3}}$ link according to the Alexander–Briggs notation. Three independent metallamacycles are interwoven through strict mechanical entanglement to form a stable dodecanuclear topology. In the case that one of the rings is removed, the other two can still exist stably. Owing to the *π*···*π* stacking interactions (3.58, 3.67, and 3.69 Å) between ligand **L2** and **E2**(OTf)_2_, the conjugate planes of the ligand and the building unit were drawn inward (Fig. S6). Note that the unique structure of compound **4**(OTf)_12_ also contributes to the complexity of NMR spectra, and the coupling relationships, as demonstrated by the ^1^H-^1^H COSY and ^1^H-^1^H nuclear Overhauser effect spectroscopy (Figs S16 and S17), facilitating definitive signal assignment for attributing different signal peaks. The ^1^H NMR spectrum of **4**(OTf)_12_ exhibited two different singlets at 1.94 and 1.90 ppm, which were assigned to the protons of the Cp* groups (Fig. S15). The aromatic protons of ligand **L2** corresponding to the pyridine moiety appeared as four distinct signals at 9.22, 9.16, 8.29, and 7.49 ppm, while the resonances of the carbazole protons were recorded at 8.72, 8.48, 8.36, 7.90, 7.75, and 7.64 ppm. Additionally, the signal belonging to the proton of **E2**(OTf)_2_ was observed at 7.00 ppm (peak assignments are provided in Section 2.6 of the Supplementary Data). The recorded hydrodynamic radius of **4**(OTf)_12_ (Fig. 4e) corroborated the proposed molecular architecture (14.36 Å) (Fig. S20). In addition, the ESI-TOF-MS spectrum of **4**(OTf)_12_ displayed a pronounced peak at *m/z* = 1521.1528, corresponding to [**4**(OTf)_12_–5OTf^−^]^5+^ (Fig. 4f, g and Figs S24–S26).

Previous studies indicated that half-sandwich-based supramolecular compounds exhibited promising photothermal conversion properties, and multiple *π*···*π* stacking interactions might enhance their photothermal conversion efficiency [46,47]. To the best of our knowledge, neither 8_18_ knots nor closed three-link chains have yet been subjected to photothermal testing. Note that compounds **3**(OTf)_16_ and **4**(OTf)_12_ contained eight and six sets of intramolecular *π*···*π* stacking interactions, respectively. Therefore, we hypothesized that compounds **3**(OTf)_16_ and **4**(OTf)_12_ would demonstrate remarkable photothermal conversion performance, thereby offering great potential for future applications in fields such as photothermal therapy, photothermal-driven actuation, or solar-powered seawater desalination. To ensure an equivalent number of *π*···*π* stacking interactions in both samples, compounds **3**(OTf)_16_ and **4**(OTf)_12_ were tested in a 3:4 molar ratio in the subsequent experiments. Ultraviolet–visible (UV–Vis) spectroscopy revealed remarkable absorption for both compounds **3**(OTf)_16_ and **4**(OTf)_12_ (Fig. 5a). Based on these results, a 730 nm laser was selected for the next photothermal conversion experiments. Moreover, the heating of compounds in methanol was recorded in real time using an infrared imager (Fig. 5b). As illustrated in Fig. 5c, both samples exhibited a significant photothermal response, with temperatures rising from 25.0 to 62.3°C and from 25.0 to 67.0°C, respectively (Figs S27 and S28). Thermogravimetric analysis also confirmed that these compounds exhibit high thermal stability (Figs S37 and S38).

**Figure 5.**
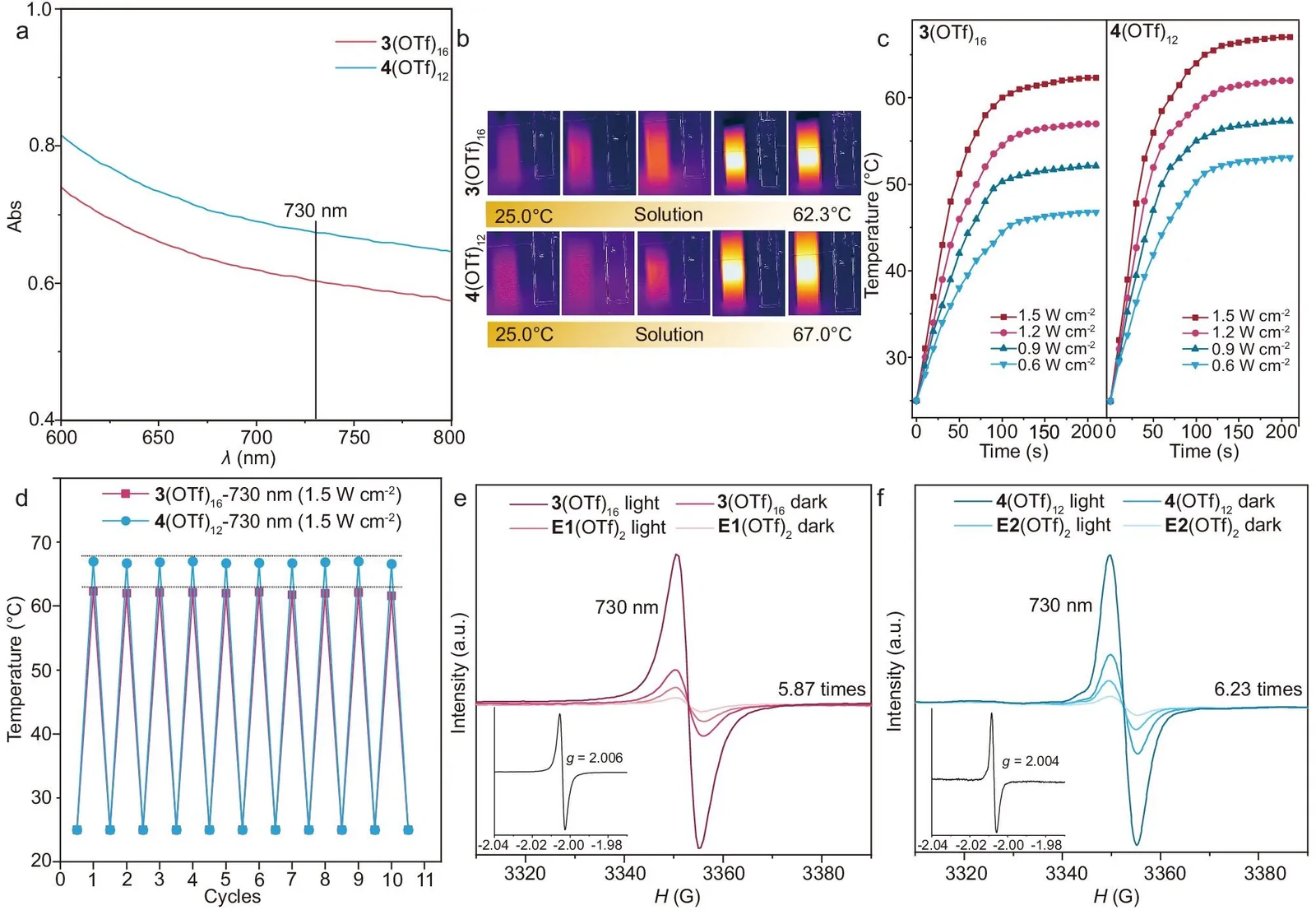
(a) UV–Vis absorption (Abs) of 8_18_ knot **3**(OTf)_16_ and closed three-link chain **4**(OTf)_12_ at 600–800 nm. (b) NIR thermal images of 8_18_ knot **3**(OTf)_16_ and closed three-link chain **4**(OTf)_12_ in the spectrophotometer cell (1 cm × 1 cm × 5 cm) under 730 nm laser irradiation. (c) Photothermal conversion of methanolic solutions of **3**(OTf)_16_ and **4**(OTf)_12_ under 730 nm. (d) Ten heating-cooling cycles of 8_18_ knot **3**(OTf)_16_ and closed three-link chain **4**(OTf)_12_ under 730 nm laser irradiation (1.5 W cm^−2^). EPR spectra of (e) 8_18_ knot **3**(OTf)_16_ and (f) closed three-link chain **4**(OTf)_12_.

In addition, over ten consecutive testing cycles, the samples exhibited no significant performance degradation, thereby demonstrating the robust stability of the compounds under photothermal conversion conditions and highlighting their potential for application across multiple fields (Fig. 5d). Linear fitting analysis revealed that the photothermal conversion efficiencies of compound **3**(OTf)_16_ and compound **4**(OTf)_12_ were from 53.82% to 57.12% and from 63.86% to 68.81% (Figs S29–S36), respectively. To further illustrate the photothermal performance level of compound **3**(OTf)_16_ and **4**(OTf)_12_, we summarize the performance data of representative photothermal materials for a comparative analysis (Tables S1 and S2). DOS calculations with and without *π*···*π* stacking interactions reveal that the narrowed bandgap promotes vibrational relaxation of excited electrons to the ground state and provides more nonradiative relaxation channels for excited state energy (Figs S40 and S41). The enhanced photothermal conversion efficiencies could be attributed to the synergistic effect of multiple stacking interactions within the supramolecular framework, which facilitated more efficient energy transfer and conversion processes. Electron paramagnetic resonance (EPR) experiments demonstrated a marked increase in signal intensity under light irradiation, confirming the generation of photoinduced organic radicals. The multiple *π*···*π* stacking interactions present in the supramolecular framework effectively suppress radiative transitions and favor non-radiative decay pathways. By promoting these non-radiative processes, the system enhances the yield of organic radicals, resulting in the observed desirable photothermal performance (Fig. 5e and f). Measurements of steady-state fluorescence spectra and fluorescence lifetimes also reveal that both compounds exhibit weak fluorescence emission intensity and short lifetimes, corresponding to a high non-radiative decay rate. A high non-radiative transition rate can remarkably boost the photothermal performance (Figs S42–S45). These findings underscore the importance of molecular design in optimizing photothermal conversion materials for specific applications.

## CONCLUSION

In summary, we utilized a strategy revolving around tuning the coordination angle, which enables precise construction of diverse and topologically distinct supramolecular architectures. Four novel interlocked and non-interlocked structures have been successfully synthesized: a molecular-tweezer-like compound **1**(OTf)_8_, a snowflake-shaped folded assembly **2**(OTf)_12_, an 8_18_ knot **3**(OTf)_16_, and a closed three-link chain **4**(OTf)_12_. This approach leverages two carbazole-based ligands with carefully tailored angular geometries, achieved through coordination-driven self-assembly of finely designed building blocks. Steric constraints prevented the smaller-angled ligand precursor **L1** from forming interlocked structures, whereas the larger-angled precursor **L2** offered greater conformational flexibility, thereby facilitating the formation of these intricate interlocked topologies. The structures of all compounds were unambiguously confirmed in the solid state by SC-XRD analysis, supported by NMR spectroscopy and ESI-TOF-MS data. We propose that multiple *π*···*π* stacking interactions within these architectures contribute to their enhanced photothermal conversion performance. Notably, compound **4**(OTf)_12_ exhibited exceptional photothermal conversion efficiency, with EPR spectroscopy providing further mechanistic insight. Additionally, our findings highlight the broad potential of the coordination-angle modulation strategy. This approach not only offers a powerful method for constructing complex supramolecular topologies but also establishes a tunable platform for designing smart materials. By simply adjusting the ligand angle, it becomes possible to systematically control the size, shape, and interlocking behavior of molecular architectures. This capability enables new applications in areas, including molecular machines, stimuli-responsive materials, and photothermal devices.

## METHODS

### Synthesis of ligand L1

Under argon, 3, 6-dibromo-9*H*-carbazole (100 mg, 0.24 mmol), 4-(4,4,5,5-tetramethyl-1,3,2-dioxaborolan-2-yl)pyridine (301 mg, 1.47 mmol), Pd(PPh_3_)_4_ (28 mg, 0.02 mmol), and K_3_PO_4_ (156 mg, 0.73 mmol) were added to degassed DMF/H_2_O (3 : 1, 30 mL). The mixture was heated at 100°C in a pressure reactor for 3 days. After cooling, the mixture was washed with water, brine, and saturated NH_4_Cl, dried over MgSO_4_, and concentrated. The product was purified by column chromatography (pentane/acetone, 1:1) and GPC (chloroform, CHCl_3_) to afford yellow solid **L1**.

### Synthesis of ligand L2

The synthesis of **L2** was carried out similarly to that of **L1** with the use of 2,7-dibromo-9*H*-carbazole (100 mg, 0.24 mmol) instead of 3,6-dibromo-9*H*-carbazole (100 mg, 0.24 mmol).

### Synthesis of 2(OTf)_12_

AgOTf (123 mg, 0.48 mmol) and [Cp*RhCl_2_]_2_ (74 mg, 0.12 mmol) were added to methanol (18 mL) at room temperature, protected from light and stirred in the dark for 12 h. The AgCl precipitate was filtered off to get a yellow solution. 2,7-Dihydroxybenzo[lmn][3,8]phenanthroline-1,3,6,8(2H, 7H)-tetraone (36 mg, 0.12 mmol) and NaOH (10 mg, 0.24 mmol) were added to the filtrate, followed by stirring for 12 h, and the mixture was treated with ether to obtain **E2**(OTf)_2_. **L1** (38.56 mg, 0.12 mmol) was added to **E2**(OTf)_2_ in methanol, and the mixture was stirred for 12 h to obtain a dark yellow solution. Single crystals were grown by diffusing ether into a methanol solution, and recrystallized from diethyl ether to afford brown block crystals **2**(OTf)_12_. ^1^H NMR (500 MHz, CD_3_OD, ppm, with respect to Cp*Rh): *δ* = 8.28 (d, 2H *J* = 5.5 Hz, pyridyl-H_a_), *δ* = 7.82 (d, 2H, *J* = 5.5 Hz, pyridyl-H_b_), *δ* = 7.32 (d, 1H, *J* = 8.00 Hz, carbazole-H_c_), *δ* = 7.88 (d, 1H, *J* = 8.00 Hz, carbazole-H_d_), *δ* = 7.67 (s, 1H, carbazole-H_e_), *δ* = 5.71 (s, 2H, naphthyl-H_f_), and *δ* = 1.72 (s, 15H, Cp*-H_g_).

### Synthesis of 3(OTf)_16_

AgOTf (123 mg, 0.48 mmol) and [Cp*RhCl_2_]_2_ (74 mg, 0.12 mmol) were added to methanol (18 mL), stirred in the dark for 12 h, and then filtered. 6,11-Dihydroxytetracene-5,12-dione (35 mg, 0.12 mmol) and NaOH (10 mg, 0.24 mmol) were added to the filtrate, stirred for 12 h (purple solution), and treated with ether to get **E1**(OTf)_2_. **L2** (38.56 mg, 0.12 mmol) was added to **E1**(OTf)_2_ in methanol, followed by stirring for 12 h. Single crystals were grown by diffusing ether into a methanol solution, and recrystallized from diethyl ether to afford dark green block crystals **3**(OTf)_16_. ^1^H NMR (500 MHz, CD_3_OD, ppm, with respect to Cp*Rh): *δ* = 8.86, 8.78 (d, 2H *J* = 6.00 Hz, *J* = 5.50 Hz pyridyl-H_a1_, H_a2_), *δ* = 8.50, 8.25 (d, 2H, *J* = 6.0 Hz, pyridyl-H_b1_, H_b2_), *δ* = 7.87, 7.79 (d, 1H, *J* = 9.0 Hz, *J* = 8.0 Hz carbazole-H_c1_, H_c2_), *δ* = 8.42, 8.38 (d, 1H, *J* = 8.0 Hz, *J* = 8.0 Hz carbazole-H_d1_, H_d2_), *δ* = 8.18, 8.09 (s, 1H, carbazole-H_e1_, H_e2_), *δ* = 8.67, (d, 2H, **E1**(OTf)_2_-H_f_), *δ* = 8.26, (d, 2H, **E1**(OTf)_2_-H_g_), and *δ* = 1.704, 1.697 (s, 15H, Cp*-H_h1_, H_h2_).

### Synthesis of 4(OTf)_12_

The synthesis of **4**(OTf)_12_ was carried out similarly to that of **3**(OTf)_16_ with the use of 2,7-dihydroxybenzo[lmn][3,8]phenanthroline-1,3,6,8(2*H*,7*H*)-tetraone (36 mg, 0.12 mmol) instead of 6,11-dihydroxytetracene-5,12-dione (35 mg, 0.12 mmol). **4**(OTf)_12_ was obtained as a black solid. ^1^H NMR (500 MHz, CD_3_OD, ppm, with respect to Cp*Rh): *δ* = 9.22, 9.16 (d, 2H *J* = 4.00 Hz, *J* = 4.50 Hz pyridyl-H_a1_, H_a2_), *δ* = 7.49, 8.29 (d, 2H, *J* = 5.0 Hz, *J* = 8.0 Hz, pyridyl-H_b1_, H_b2_), *δ* = 7.90, 7.75 (d, 1H, *J* = 8.5 Hz, *J* = 6.5 Hz carbazole-H_c1_, H_c2_), *δ* = 8.72, 8.48 (d, 1H, *J* = 10.0 Hz, *J* = 7.0 Hz carbazole-H_d1_, H_d2_), *δ* = 8.36, 7.64 (s, 1H, carbazole-H_e1_, H_e2_), *δ* = 7.00, (d, 2H, **E2**(OTf)_2_-H_f_), and *δ* = 1.94, 1.90 (s, 15H, Cp*-H_g1_, H_g2_).

### Investigation of photothermal properties

To ensure equal conjugated *π*-area, compounds **3**(OTf)_16_ and **4**(OTf)_12_ were mixed in a 3:4 molar ratio with 3 mL CH_3_OH. After complete dissolution, 2.0 mL of the solution was transferred to a quartz cuvette for absorbance measurement and irradiated with a 730-nm laser at varying current intensities, with temperature changes monitored via an infrared camera. Additionally, 0.5 mL aliquots of the methanol solutions were placed in sample vessels, irradiated similarly, and temperature variations were detected using the same infrared camera.

## Supplementary Material

nwag432_Supplemental_Files
