# Supplementary material for: Efficient synthesis of complex supramolecular topologies via coordination angle modulation
Source: Natl Sci Rev. 2026 Jul 13;13(18):nwag432. doi: 10.1093/nsr/nwag432 (PMC13629236; doi:10.1093/nsr/nwag432)

checkCIF/PLATON report

Structure factors have been supplied for datablock(s) cu_20250311ru1203nacl_a

THIS REPORT IS FOR GUIDANCE ONLY. IF USED AS PART OF A REVIEW PROCEDURE FOR PUBLICATION, IT SHOULD NOT REPLACE THE EXPERTISE OF AN EXPERIENCED CRYSTALLOGRAPHIC REFEREE.

No syntax errors found. CIF dictionary Interpreting this report

Datablock: cu_20250311ru1203nacl_a

Bond precision:	C-C = 0.0298 A		Wavelength=1.54178
Cell:	a=20.2428(17)	b=22.0368(17)	c=23.2357(18)
	alpha=80.668(4)	beta=76.437(5)	gamma=76.641(5)
Temperature:	150 K		

	Calculated	Reported
Volume	9739.4(14)	9739.3(14)
Space group	P -1	P -1
Hall group	-P 1	-P 1
Moiety formula	C336 H292 N30 O36 Rh12, C20 H30 Rh, 10(C F3 O3 S) [+ solvent]	C336 H292 N30 O36 Rh12, C20 H30 Rh, 10(C F3 O3 S), 6[CF3SO3]
Sum formula	C366 H322 F30 N30 O66 Rh13 S10 [+ solvent]	C372 H322 F48 N30 O84 Rh13 S16
Mr	8424.99	9319.37
Dx,g cm-3	1.436	1.589
Z	1	1
Mu (mm-1)	5.574	6.034
F000	4271.0	4709.0
F000'	4288.37	
h,k,lmax	23,25,27	23,25,27
Nref	32187	31315
Tmin,Tmax	0.344,0.405	0.378,0.753
Tmin'	0.260	

```
Correction method= # Reported T Limits: Tmin=0.378 Tmax=0.753
AbsCorr = MULTI-SCAN

Data completeness= 0.973            Theta(max)= 63.821

R(reflections)= 0.1356( 15348)                      wR2(reflections)= 0.3508( 31315)

S = 1.018                   Npar= 2342
```

The following ALERTS were generated. Each ALERT has the format
test-name_ALERT_alert-type_alert-level.
Click on the hyperlinks for more details of the test.

Alert level B

```
PLAT342_ALERT_3_B Low Bond Precision on  C-C Bonds ..............    0.02984 Ang.
```

Author Response: Poor data quality in context with large assymetric unit and heavy rhodium atoms in the lattice.

```
PLAT973_ALERT_2_B Check Calcd Positive Resid. Density on       Rh4       1.55 eA-3
```

Author Response: The high residual density near rhodium atoms result from tail effect and poor data quality.

```
PLAT973_ALERT_2_B Check Calcd Positive Resid. Density on       Rh5       1.53 eA-3
```

Author Response: The high residual density near rhodium atoms result from tail effect and poor data quality.

Alert level C

```
RINTA01_ALERT_3_C  The value of Rint is greater than 0.12
            Rint given   0.123
THETM01_ALERT_3_C  The value of sine(theta_max)/wavelength is less than 0.590
            Calculated sin(theta_max)/wavelength =    0.5821
PLAT020_ALERT_3_C The Value of Rint is Greater Than 0.12 .........      0.123 Report
PLAT026_ALERT_3_C Ratio Observed / Unique Reflections (too) Low ..        49% Check
PLAT029_ALERT_3_C _diffrn_measured_fraction_theta_full value Low .      0.973 Why?
PLAT082_ALERT_2_C High R1 Value ..................................       0.14 Report
PLAT084_ALERT_3_C High wR2 Value (i.e. > 0.25) ...................       0.35 Report
PLAT242_ALERT_2_C Low   MainResAtom  Ueq as Compared to Neighbours        Rh6 Check
PLAT242_ALERT_2_C Low   MainResAtom  Ueq as Compared to Neighbours        Rh4 Check
PLAT244_ALERT_4_C Low  MinorResAtom  Ueq as Compared to Neighbours         S1 Check
PLAT244_ALERT_4_C Low  MinorResAtom  Ueq as Compared to Neighbours         S4 Check
PLAT250_ALERT_2_C Large U3/U1 Ratio for <U(i,j)> Tensor(Resd    2)        2.2 Note
PLAT250_ALERT_2_C Large U3/U1 Ratio for <U(i,j)> Tensor(Resd    7)        2.2 Note
PLAT260_ALERT_2_C Large Average Ueq of Residue Including       Rh1      0.111 Check
```

```
PLAT260_ALERT_2_C Large Average Ueq of Residue Including       Rh4      0.162 Check
PLAT260_ALERT_2_C Large Average Ueq of Residue Including        S1      0.165 Check
PLAT260_ALERT_2_C Large Average Ueq of Residue Including        S2      0.157 Check
PLAT260_ALERT_2_C Large Average Ueq of Residue Including        S3      0.159 Check
PLAT260_ALERT_2_C Large Average Ueq of Residue Including        S4      0.194 Check
PLAT260_ALERT_2_C Large Average Ueq of Residue Including        S5      0.182 Check
PLAT363_ALERT_2_C Long   C(sp3)-C(sp2) Bond  C117     - C183     .        1.65 Ang.
PLAT369_ALERT_2_C Long   C(sp2)-C(sp2) Bond  C12      - C41      .        1.54 Ang.
PLAT369_ALERT_2_C Long   C(sp2)-C(sp2) Bond  C18      - C86      .        1.54 Ang.
PLAT369_ALERT_2_C Long   C(sp2)-C(sp2) Bond  C65      - C95      .        1.53 Ang.
PLAT410_ALERT_2_C Short Intra H...H Contact  H25      ..H61      .        1.94 Ang.
                                                      x,y,z  =      1_555 Check
PLAT410_ALERT_2_C Short Intra H...H Contact  H87      ..H108     .        1.95 Ang.
                                                      x,y,z  =      1_555 Check
PLAT413_ALERT_2_C Short Inter XH3 .. XHn     H13B     ..H16H     .        2.09 Ang.
                                                 2-x,-y,1-z  =      2_756 Check
PLAT905_ALERT_3_C Negative K value in the Analysis of Variance ...     -3.158 Report
PLAT911_ALERT_3_C Missing FCF Refl Between Thmin & STh/L=    0.582        870 Report
                6  0  0,  10  0  0,  -7  1  0,  -6  1  0,  -4  2  0,   4  2  0,
                6  3  0, -11  4  0,   3  4  0,  -9  5  0,  -7  5  0, -11  6  0,
               -8  6  0,  -5  6  0,   4  6  0,  -9  7  0,  -7  7  0,  -6  7  0,
               -4  7  0,  -6  8  0,  -9  9  0,  -3  9  0,  -2  9  0,   1  9  0,
              -10 10  0,  -9 10  0,  -7 10  0,  -7 11  0,  -1 12  0,   7 12  0,
                   (  840 More NOT listed: see .ckf listing file)
PLAT973_ALERT_2_C Check Calcd Positive Resid. Density on       Rh2       1.44 eA-3
```

Author Response: The high residual density near rhodium atoms result from tail effect and poor data quality.

```
PLAT973_ALERT_2_C Check Calcd Positive Resid. Density on       Rh6       1.43 eA-3
```

Author Response: The high residual density near rhodium atoms result from tail effect and poor data quality.

```
PLAT973_ALERT_2_C Check Calcd Positive Resid. Density on       Rh3       1.39 eA-3
```

Author Response: The high residual density near rhodium atoms result from tail effect and poor data quality.

```
PLAT973_ALERT_2_C Check Calcd Positive Resid. Density on       Rh7       1.37 eA-3
```

Author Response: The high residual density near rhodium atoms result from tail effect and poor data quality.

```
PLAT973_ALERT_2_C Check Calcd Positive Resid. Density on       Rh1       1.21 eA-3
```

Author Response: The high residual density near rhodium atoms result from tail effect and poor data quality.

```
PLAT977_ALERT_2_C Check Negative Difference Density on H17R      .      -0.43 eA-3
PLAT977_ALERT_2_C Check Negative Difference Density on H18B      .      -0.31 eA-3
```

Alert level G

```
FORMU01_ALERT_2_G  There is a discrepancy between the atom counts in the
            _chemical_formula_sum and the formula from the _atom_site* data.
            Atom count from _chemical_formula_sum:C372 H322 F48 N30 O84 Rh13 S16
            Atom count from the _atom_site data:  C366 H322 F30 N30 O66 Rh13 S10
CELLZ01_ALERT_1_G Difference between formula and atom_site contents detected.
CELLZ01_ALERT_1_G ALERT: Large difference may be due to a
            symmetry error - see SYMMG tests
           From the CIF: _cell_formula_units_Z    1
           From the CIF: _chemical_formula_sum  C372 H322 F48 N30 O84 Rh13 S16
           TEST: Compare cell contents of formula and atom_site data

           atom    Z*formula  cif sites diff
           C        372.00    366.00    6.00
           H        322.00    322.00    0.00
           F         48.00     30.00   18.00
           N         30.00     30.00    0.00
           O         84.00     66.00   18.00
           Rh        13.00     13.00    0.00
           S         16.00     10.00    6.00
PLAT002_ALERT_2_G Number of Distance or Angle Restraints on AtSite          4 Note
PLAT003_ALERT_2_G Number of Uiso or U(i,j)  Restrained non-H-Atoms        258 Report
PLAT007_ALERT_5_G Number of Unrefined Donor-H Atoms .............           3 Report
              H8      H10     H15
PLAT041_ALERT_1_G Calc. and Reported SumFormula    Strings  Differ     Please Check
              Calc: C366 H322 F30 N30 O66 Rh13 S10
              Rep.: C372 H322 F48 N30 O84 Rh13 S16
PLAT042_ALERT_1_G Calc. and Reported MoietyFormula Strings  Differ     Please Check
              Calc: C336 H292 N30 O36 Rh12, C20 H30 Rh, 10(C F3 O3
                     S)
              Rep.: C336 H292 N30 O36 Rh12, C20 H30 Rh, 10(C F3 O3
                     S), 6[CF3SO3]
PLAT051_ALERT_1_G Mu(calc) and Mu(cif) Ratio Differs from 1.0 by .       7.62 %
PLAT083_ALERT_2_G SHELXL Second Parameter in WGHT  Unusually Large     100.00 Why ?
PLAT172_ALERT_4_G The CIF-Embedded .res File Contains DFIX Records          2 Report
PLAT177_ALERT_4_G The CIF-Embedded .res File Contains DELU Records          1 Report
PLAT178_ALERT_4_G The CIF-Embedded .res File Contains SIMU Records          1 Report
PLAT188_ALERT_3_G A Non-default SIMU Restraint Value has been used     0.0100 Report
PLAT244_ALERT_4_G Low  MinorResAtom  Ueq as Compared to Neighbours        C62 Check
PLAT244_ALERT_4_G Low  MinorResAtom  Ueq as Compared to Neighbours       C170 Check
PLAT244_ALERT_4_G Low  MinorResAtom  Ueq as Compared to Neighbours       C143 Check
PLAT244_ALERT_4_G Low  MinorResAtom  Ueq as Compared to Neighbours       C162 Check
PLAT244_ALERT_4_G Low  MinorResAtom  Ueq as Compared to Neighbours       C157 Check
PLAT335_ALERT_2_G Check Large C6 Ring C-C Range C3       -C47            0.17 Ang.
PLAT380_ALERT_4_G Incorrectly? Oriented X(sp2)-Methyl Moiety .....       C100 Check
PLAT380_ALERT_4_G Incorrectly? Oriented X(sp2)-Methyl Moiety .....       C183 Check
PLAT380_ALERT_4_G Incorrectly? Oriented X(sp2)-Methyl Moiety .....       C118 Check
PLAT380_ALERT_4_G Incorrectly? Oriented X(sp2)-Methyl Moiety .....       C158 Check
PLAT380_ALERT_4_G Incorrectly? Oriented X(sp2)-Methyl Moiety .....       C165 Check
PLAT380_ALERT_4_G Incorrectly? Oriented X(sp2)-Methyl Moiety .....       C167 Check
PLAT380_ALERT_4_G Incorrectly? Oriented X(sp2)-Methyl Moiety .....       C176 Check
PLAT432_ALERT_2_G Short Inter X...Y Contact  F11      ..C4       .       2.92 Ang.
                                                2-x,1-y,1-z  =      2_766 Check
```

```
PLAT432_ALERT_2_G Short Inter X...Y Contact  O17      ..C76      .       2.90 Ang.
                                              x,1+y,z  =     1_565 Check
PLAT434_ALERT_2_G Short Inter HL..HL Contact F2       ..F14      .       2.83 Ang.
                                          2-x,1-y,1-z  =     2_766 Check
PLAT434_ALERT_2_G Short Inter HL..HL Contact F14      ..F14      .       2.71 Ang.
                                          2-x,1-y,1-z  =     2_766 Check
PLAT606_ALERT_4_G Solvent Accessible VOID(S) in  Crystal Structure          ! Info
PLAT720_ALERT_4_G Number of Unusual/Non-Standard Labels .........          1 Note
              Ha
PLAT790_ALERT_4_G Centre of Gravity not Within Unit-Cell: Resd.  #          6 Note
              C F3 O3 S
PLAT860_ALERT_3_G Number of Least-Squares Restraints ............       2495 Note
PLAT868_ALERT_4_G ALERTS Due to the Use of _smtbx_masks Suppressed          ! Info
PLAT883_ALERT_1_G Absent Datum for _atom_sites_solution_primary ..     Please Do !
PLAT910_ALERT_3_G Missing FCF Reflection(s) Below Theta(Min)[Deg]=       2.29 Note
                0  1  0,   0  0  1,
PLAT933_ALERT_2_G Number of HKL-OMIT Records in Embedded .res File        594 Note
              -12 -8 10, -12 13  0, -11 -4  6, -11  0  1, -11  4  0, -11  6  0,
              -10-14  8, -10-13  9, -10 -7 11, -10 -6 10, -10 -3 11, -10  1  1,
              -10  3  1, -10 10  0,  -9-12  6,  -9-11 12,  -9 -8  8,  -9 -7  9,
               -9 -7 10,  -9 -5 10,  -9 -2  9,  -9  0  3,  -9  5  0,  -9  7  0,
               -9  9  0,  -9 10  0,  -9 10  1,  -9 11  1,  -9 13  0,  -8 -9  6,
               -8 -7  7,  -8 -6  8,  -8 -6  9,  -8 -4  9,  -8 -3  6,  -8 -3 10,
               -8  0 12,  -8  1 10,  -8  4  3,  -8  4 10,  -8  6  0,  -8 13  0,
               -8 16  0,  -7 -4  6,  -7 -1  4,  -7 -1  6,  -7  0  2,  -7  0  7,
               -7  0  8,  -7  0 11,
                   (  544 More NOT listed: see .ckf listing file)
PLAT941_ALERT_3_G Average HKL Measurement Multiplicity ..........        3.3 Low
PLAT969_ALERT_5_G The 'Henn et al.' R-Factor-gap value ..........      1.745 Note
              Predicted wR2: Based on SigI**2 20.10 or SHELX Weight 34.47
PLAT978_ALERT_2_G Number C-C Bonds with Positive Residual Density.          0 Info
PLAT992_ALERT_5_G Repd & Actual _reflns_number_gt Values Differ by          2 Check
PLAT994_ALERT_1_G SHELXL .ins Contains no or MERG 0 Instruction ..          ! Note
```

---

0 **ALERT level A** = Most likely a serious problem - resolve or explain
3 **ALERT level B** = A potentially serious problem, consider carefully
36 **ALERT level C** = Check. Ensure it is not caused by an omission or oversight
44 **ALERT level G** = General information/check it is not something unexpected

7 ALERT type 1 CIF construction/syntax error, inconsistent or missing data
39 ALERT type 2 Indicator that the structure model may be wrong or deficient
13 ALERT type 3 Indicator that the structure quality may be low
21 ALERT type 4 Improvement, methodology, query or suggestion
3 ALERT type 5 Informative message, check

It is advisable to attempt to resolve as many as possible of the alerts in all categories. Often the minor alerts point to easily fixed oversights, errors and omissions in your CIF or refinement strategy, so attention to these fine details can be worthwhile. It is up to the individual to critically assess their own results and, if necessary, seek expert advice.

PLATON version of 15/01/2026; check.def file version of 02/01/2026

duplicate check

No duplication found

Datablock cu_20250311ru1203nacl_a - ellipsoid plot

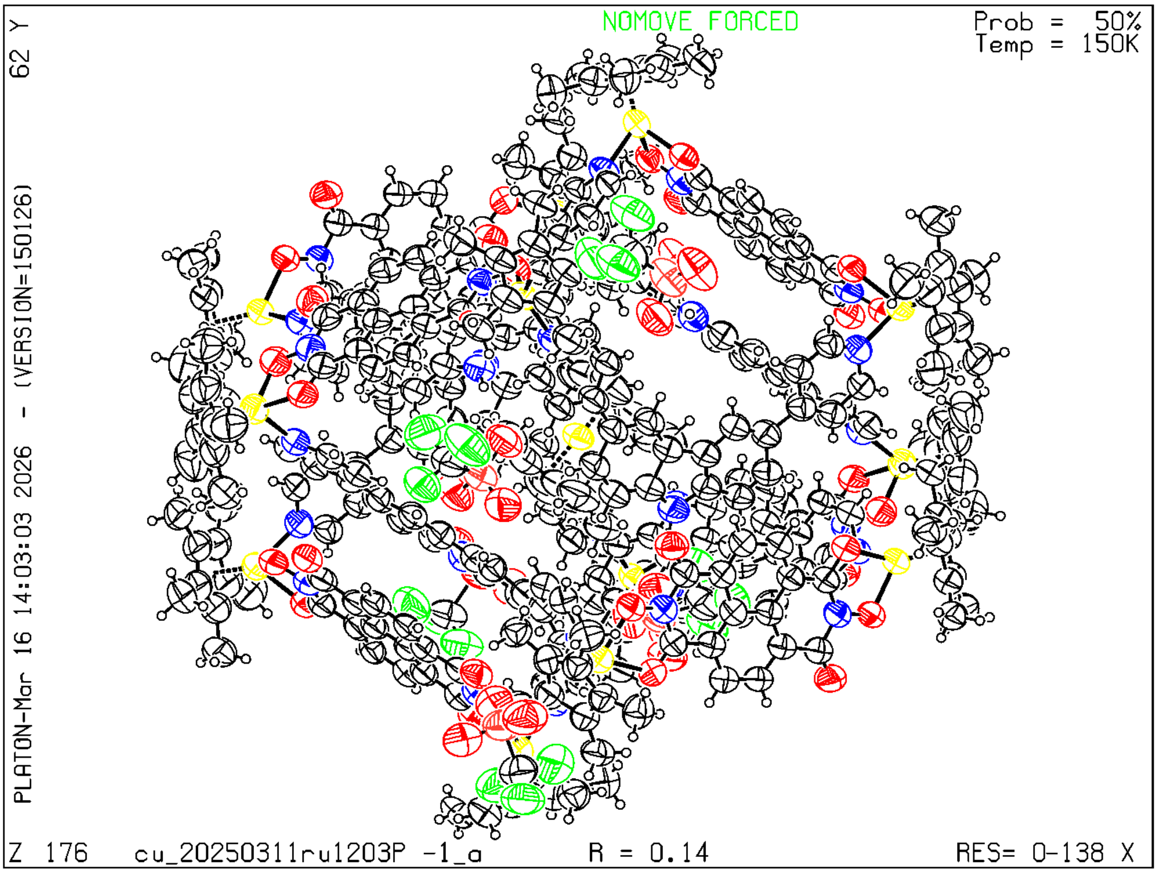

Supplement: Supplementary file 1 [file nwag432_supplemental_files.zip › checkcif-2.pdf]
