# Supplementary material for: Efficient synthesis of complex supramolecular topologies via coordination angle modulation
Source: Natl Sci Rev. 2026 Jul 13;13(18):nwag432. doi: 10.1093/nsr/nwag432 (PMC13629236; doi:10.1093/nsr/nwag432)

checkCIF/PLATON report

Structure factors have been supplied for datablock(s) cu_dll_w6405_0m_sq_sq

THIS REPORT IS FOR GUIDANCE ONLY. IF USED AS PART OF A REVIEW PROCEDURE FOR PUBLICATION, IT SHOULD NOT REPLACE THE EXPERTISE OF AN EXPERIENCED CRYSTALLOGRAPHIC REFEREE.

No syntax errors found.   CIF dictionary   Interpreting this report

Datablock: cu_dll_w6405_0m_sq_sq

Bond precision:	C- C = 0.0001 A		Wavelength=1.54178
Cell:	a=53.5860(18)	b=22.2676(8)	c=54.8920(19)
	alpha=90	beta=118.834(2)	gamma=90
Temperature:	150 K		

	Calculated	Reported
Volume	57378(4)	57379(4)
Space group	P 2	P 1 2 1
Hall group	P 2y	P 2y
Moiety formula	C480 H424 N24 O32 Rh16, 2(C F3 O3 S) [+ solvent]	C480 H424 N24 O32 Rh16, 2(C F3 O3 S)
Sum formula	C482 H424 F6 N24 O38 Rh16 S2 [+ solvent]	C482 H424 F6 N24 O38 Rh16 S2
Mr	8985.06	8985.11
Dx,g cm-3	1.040	1.040
Z	4	4
Mu (mm-1)	4.106	4.106
F000	18376.0	18376.0
F000'	18431.12	
h,k,lmax	59,24,61	59,24,61
Nref	167605[86329]	145651
Tmin,Tmax	0.462,0.540	0.494,0.578
Tmin'	0.419	

```
Correction method= # Reported T Limits: Tmin=0.494 Tmax=0.578
AbsCorr = MULTI-SCAN

Data completeness= 1.69/0.87       Theta(max)= 59.460

R(reflections)= 0.1031( 51962)                  wR2(reflections)=
                                                0.2316(145651)
S = 1.107                 Npar= 10183
```

The following ALERTS were generated. Each ALERT has the format
test-name_ALERT_alert-type_alert-level.
Click on the hyperlinks for more details of the test.

Alert level B

```
THETM01_ALERT_3_B  The value of sine(theta_max)/wavelength is less than 0.575
            Calculated sin(theta_max)/wavelength =    0.5586
```

Author Response: This is due to the poor diffraction of the crystal.

```
PLAT026_ALERT_3_B Ratio Observed / Unique Reflections (too) Low ..        36% Check
```

Author Response: This is due to the poor diffraction of the crystal.

Alert level C

```
ABSTY02_ALERT_1_C  An _exptl_absorpt_correction_type has been given without
            a literature citation. This should be contained in the
            _exptl_absorpt_process_details field.
            Absorption correction given as multi-scan
PLAT018_ALERT_1_C _diffrn_measured_fraction_theta_max .NE. *_full          ! Check
PLAT034_ALERT_1_C No Flack Parameter Given. Z > Si, NonCentro ....     Please Do !
PLAT241_ALERT_2_C High  MainResAtom  Ueq as Compared to Neighbours    Rh8_200 Check
PLAT242_ALERT_2_C Low   MainResAtom  Ueq as Compared to Neighbours    Rh3_100 Check
PLAT242_ALERT_2_C Low   MainResAtom  Ueq as Compared to Neighbours      O3_17 Check
PLAT242_ALERT_2_C Low   MainResAtom  Ueq as Compared to Neighbours    Rh1_400 Check
PLAT242_ALERT_2_C Low   MainResAtom  Ueq as Compared to Neighbours    Rh6_400 Check
PLAT242_ALERT_2_C Low   MainResAtom  Ueq as Compared to Neighbours    Rh3_500 Check
PLAT242_ALERT_2_C Low   MainResAtom  Ueq as Compared to Neighbours    Rh7_500 Check
PLAT242_ALERT_2_C Low   MainResAtom  Ueq as Compared to Neighbours      O1_31 Check
PLAT260_ALERT_2_C Large Average Ueq of Residue Including   Rh1_100      0.147 Check
PLAT260_ALERT_2_C Large Average Ueq of Residue Including   Rh1_200      0.203 Check
PLAT260_ALERT_2_C Large Average Ueq of Residue Including   Rh1_400      0.181 Check
PLAT260_ALERT_2_C Large Average Ueq of Residue Including   Rh1_500      0.216 Check
PLAT260_ALERT_2_C Large Average Ueq of Residue Including     S1_42      0.172 Check
PLAT260_ALERT_2_C Large Average Ueq of Residue Including     S1_45      0.150 Check
PLAT260_ALERT_2_C Large Average Ueq of Residue Including     S1_57      0.204 Check
PLAT260_ALERT_2_C Large Average Ueq of Residue Including     S1_68      0.205 Check
PLAT410_ALERT_2_C Short Intra H...H Contact  H4_2     ..H7_2     .       1.92 Ang.
                                                 x,y,z  =      1_555 Check
```

```
PLAT410_ALERT_2_C Short Intra H...H Contact  H15_2    ..H21_2    .       1.91 Ang.
                                                  x,y,z  =      1_555 Check
PLAT410_ALERT_2_C Short Intra H...H Contact  H15_5    ..H21_5    .       1.94 Ang.
                                                  x,y,z  =      1_555 Check
PLAT410_ALERT_2_C Short Intra H...H Contact  H17_5    ..H19_5    .       1.98 Ang.
                                                  x,y,z  =      1_555 Check
PLAT410_ALERT_2_C Short Intra H...H Contact  H4_7     ..H7_7     .       1.92 Ang.
                                                  x,y,z  =      1_555 Check
PLAT410_ALERT_2_C Short Intra H...H Contact  H17_7    ..H19_7    .       1.92 Ang.
                                                  x,y,z  =      1_555 Check
PLAT410_ALERT_2_C Short Intra H...H Contact  H2_8     ..H11_8    .       1.98 Ang.
                                                  x,y,z  =      1_555 Check
PLAT410_ALERT_2_C Short Intra H...H Contact  H2_12    ..H11_12   .       1.96 Ang.
                                                  x,y,z  =      1_555 Check
PLAT410_ALERT_2_C Short Intra H...H Contact  H2_16    ..H11_16   .       1.98 Ang.
                                                  x,y,z  =      1_555 Check
PLAT410_ALERT_2_C Short Intra H...H Contact  H4_16    ..H7_16    .       1.95 Ang.
                                                  x,y,z  =      1_555 Check
PLAT410_ALERT_2_C Short Intra H...H Contact  H15_16   ..H21_16   .       1.93 Ang.
                                                  x,y,z  =      1_555 Check
PLAT410_ALERT_2_C Short Intra H...H Contact  H17_16   ..H19_16   .       1.94 Ang.
                                                  x,y,z  =      1_555 Check
PLAT410_ALERT_2_C Short Intra H...H Contact  H2_15    ..H11_15   .       1.99 Ang.
                                                  x,y,z  =      1_555 Check
PLAT410_ALERT_2_C Short Intra H...H Contact  H4_15    ..H7_15    .       1.99 Ang.
                                                  x,y,z  =      1_555 Check
PLAT410_ALERT_2_C Short Intra H...H Contact  H4_19    ..H7_19    .       1.97 Ang.
                                                  x,y,z  =      1_555 Check
PLAT410_ALERT_2_C Short Intra H...H Contact  H15_19   ..H21_19   .       1.92 Ang.
                                                  x,y,z  =      1_555 Check
PLAT410_ALERT_2_C Short Intra H...H Contact  H2_22    ..H11_22   .       1.93 Ang.
                                                  x,y,z  =      1_555 Check
PLAT410_ALERT_2_C Short Intra H...H Contact  H15_22   ..H21_22   .       1.90 Ang.
                                                  x,y,z  =      1_555 Check
PLAT410_ALERT_2_C Short Intra H...H Contact  H17_22   ..H19_22   .       1.96 Ang.
                                                  x,y,z  =      1_555 Check
PLAT410_ALERT_2_C Short Intra H...H Contact  H2_23    ..H11_23   .       1.94 Ang.
                                                  x,y,z  =      1_555 Check
PLAT410_ALERT_2_C Short Intra H...H Contact  H15_23   ..H21_23   .       1.95 Ang.
                                                  x,y,z  =      1_555 Check
PLAT410_ALERT_2_C Short Intra H...H Contact  H15_24   ..H21_24   .       1.98 Ang.
                                                  x,y,z  =      1_555 Check
PLAT410_ALERT_2_C Short Intra H...H Contact  H2_27    ..H11_27   .       1.98 Ang.
                                                  x,y,z  =      1_555 Check
PLAT410_ALERT_2_C Short Intra H...H Contact  H2_32    ..H11_32   .       1.94 Ang.
                                                  x,y,z  =      1_555 Check
PLAT410_ALERT_2_C Short Intra H...H Contact  H4_32    ..H7_32    .       1.97 Ang.
                                                  x,y,z  =      1_555 Check
PLAT413_ALERT_2_C Short Inter XH3 .. XHn     H2_3     ..H9A_44   .       2.05 Ang.
                                               2-x,y,-z  =      2_755 Check
PLAT413_ALERT_2_C Short Inter XH3 .. XHn     H17_3    ..H10B_39  .       2.05 Ang.
                                          1-x,-1+y,-1-z  =      2_644 Check
PLAT413_ALERT_2_C Short Inter XH3 .. XHn     H8B_36   ..H9A_61   .       2.10 Ang.
                                          1-x,-1+y,-1-z  =      2_644 Check
PLAT413_ALERT_2_C Short Inter XH3 .. XHn     H8B_36   ..H9C_61   .       2.11 Ang.
                                          1-x,-1+y,-1-z  =      2_644 Check
PLAT413_ALERT_2_C Short Inter XH3 .. XHn     H8C_38   ..H8B_49   .       2.05 Ang.
```

```
                                                  1-x,y,-z  =      2_655 Check
PLAT413_ALERT_2_C Short Inter XH3 .. XHn     H8B_40   ..H7A_1    .       2.06 Ang.
                                                     x,y,z  =      1_555 Check
PLAT413_ALERT_2_C Short Inter XH3 .. XHn     H2_10    ..H8B_55   .       2.06 Ang.
                                              2-x,-1+y,1-z  =      2_746 Check
PLAT413_ALERT_2_C Short Inter XH3 .. XHn     H2_10    ..H8C_55   .       2.01 Ang.
                                              2-x,-1+y,1-z  =      2_746 Check
PLAT413_ALERT_2_C Short Inter XH3 .. XHn     H2_17    ..H10A_48  .       2.02 Ang.
                                                  x,-1+y,z  =      1_545 Check
PLAT413_ALERT_2_C Short Inter XH3 .. XHn     H8B_43   ..H9C_66   .       2.06 Ang.
                                                  x,-1+y,z  =      1_545 Check
PLAT413_ALERT_2_C Short Inter XH3 .. XHn     H8B_48   ..H9C_58   .       2.10 Ang.
                                                     x,y,z  =      1_555 Check
PLAT413_ALERT_2_C Short Inter XH3 .. XHn     H9B_49   ..H9A_35   .       2.14 Ang.
                                                 x,1+y,1+z  =      1_566 Check
PLAT413_ALERT_2_C Short Inter XH3 .. XHn     H6A_53   ..H8A_63   .       2.11 Ang.
                                                 2-x,y,1-z  =      2_756 Check
PLAT413_ALERT_2_C Short Inter XH3 .. XHn     H6A_53   ..H8C_63   .       2.06 Ang.
                                                 2-x,y,1-z  =      2_756 Check
PLAT413_ALERT_2_C Short Inter XH3 .. XHn     H10A_54  ..H9A_56   .       2.04 Ang.
                                                  1-x,y,-z  =      2_655 Check
PLAT413_ALERT_2_C Short Inter XH3 .. XHn     H10B_54  ..H9C_56   .       2.01 Ang.
                                                  1-x,y,-z  =      2_655 Check
PLAT413_ALERT_2_C Short Inter XH3 .. XHn     H2_18    ..H7B_56   .       2.09 Ang.
                                                1-x,1+y,-z  =      2_665 Check
PLAT413_ALERT_2_C Short Inter XH3 .. XHn     H2_18    ..H7C_56   .       2.04 Ang.
                                                1-x,1+y,-z  =      2_665 Check
PLAT413_ALERT_2_C Short Inter XH3 .. XHn     H17_20   ..H7C_65   .       2.13 Ang.
                                                     x,y,z  =      1_555 Check
PLAT413_ALERT_2_C Short Inter XH3 .. XHn     H2_25    ..H8C_60   .       2.12 Ang.
                                               -1+x,-1+y,z  =      1_445 Check
PLAT413_ALERT_2_C Short Inter XH3 .. XHn     H7A_46   ..H7C_60   .       2.13 Ang.
                                                  2-x,y,-z  =      2_755 Check
PLAT413_ALERT_2_C Short Inter XH3 .. XHn     H7A_50   ..H7C_41   .       2.00 Ang.
                                                   x,1+y,z  =      1_565 Check
PLAT413_ALERT_2_C Short Inter XH3 .. XHn     H7C_50   ..H7C_41   .       2.09 Ang.
                                                   x,1+y,z  =      1_565 Check
PLAT413_ALERT_2_C Short Inter XH3 .. XHn     H3_26    ..H7C_70   .       2.02 Ang.
                                               2-x,-1+y,-z  =      2_745 Check
PLAT413_ALERT_2_C Short Inter XH3 .. XHn     H3_29    ..H10A_65  .       2.01 Ang.
                                                2-x,1+y,-z  =      2_765 Check
PLAT413_ALERT_2_C Short Inter XH3 .. XHn     H3_29    ..H10B_65  .       2.09 Ang.
                                                2-x,1+y,-z  =      2_765 Check
PLAT413_ALERT_2_C Short Inter XH3 .. XHn     H3_33    ..H6A_36   .       2.05 Ang.
                                              1-x,1+y,-1-z  =      2_664 Check
PLAT413_ALERT_2_C Short Inter XH3 .. XHn     H17_33   ..H10A_63  .       2.13 Ang.
                                                2-x,1+y,-z  =      2_765 Check
PLAT413_ALERT_2_C Short Inter XH3 .. XHn     H6A_61   ..H9C_65   .       2.14 Ang.
                                                   x,1+y,z  =      1_565 Check
PLAT413_ALERT_2_C Short Inter XH3 .. XHn     H6A_65   ..H8B_70   .       2.09 Ang.
                                               2-x,-1+y,-z  =      2_745 Check
PLAT413_ALERT_2_C Short Inter XH3 .. XHn     H6A_65   ..H8C_70   .       2.06 Ang.
                                               2-x,-1+y,-z  =      2_745 Check
PLAT420_ALERT_2_C D-H Bond Without Acceptor  N2_2     --H2A_2    .    Please Check
PLAT420_ALERT_2_C D-H Bond Without Acceptor  N2_5     --H2A_5    .    Please Check
PLAT420_ALERT_2_C D-H Bond Without Acceptor  N2_7     --H2A_7    .    Please Check
PLAT420_ALERT_2_C D-H Bond Without Acceptor  N2_8     --H2A_8    .    Please Check
```

```
PLAT420_ALERT_2_C D-H Bond Without Acceptor  N2_12    --H2A_12   .     Please Check
PLAT420_ALERT_2_C D-H Bond Without Acceptor  N2_16    --H2A_16   .     Please Check
PLAT420_ALERT_2_C D-H Bond Without Acceptor  N2_19    --H2A_19   .     Please Check
PLAT420_ALERT_2_C D-H Bond Without Acceptor  N2_22    --H2A_22   .     Please Check
PLAT420_ALERT_2_C D-H Bond Without Acceptor  N2_23    --H2A_23   .     Please Check
PLAT420_ALERT_2_C D-H Bond Without Acceptor  N2_24    --H2A_24   .     Please Check
PLAT420_ALERT_2_C D-H Bond Without Acceptor  N2_27    --H2A_27   .     Please Check
PLAT420_ALERT_2_C D-H Bond Without Acceptor  N2_28    --H2A_28   .     Please Check
PLAT420_ALERT_2_C D-H Bond Without Acceptor  N2_32    --H2A_32   .     Please Check
PLAT420_ALERT_2_C D-H Bond Without Acceptor  N2_15    --H2A_15   .     Please Check
PLAT420_ALERT_2_C D-H Bond Without Acceptor  N2_11    --H2A_11   .     Please Check
PLAT420_ALERT_2_C D-H Bond Without Acceptor  N2_30    --H2A_30   .     Please Check
PLAT790_ALERT_4_C Centre of Gravity not Within Unit-Cell: Resd.  #          1 Note
              C480 H424 N24 O32 Rh16
PLAT905_ALERT_3_C Negative K value in the Analysis of Variance ...     -6.177 Report
PLAT905_ALERT_3_C Negative K value in the Analysis of Variance ...     -0.071 Report
PLAT910_ALERT_3_C Missing FCF Reflection(s) Below Theta(Min)[Deg]=       1.65 Note
                1  0  0,  -1  0  1,   0  0  1,   1  0  1,  -1  0  2,
PLAT911_ALERT_3_C Missing FCF Refl Between Thmin & STh/L=    0.559       1242 Report
                0  4  0,   2  0  0,   2  2  0,   6  2  0,   8  2  0,  14  0  0,
               15  0  0,  36 18  0,  38 17  0,  40 16  0,  47 11  0,  48 10  0,
               52  3  0, -45 13  1,  -9  3  1,  -7  0  1,  -6  2  1,   3  0  1,
               10  1  1,  24 22  1,  41 15  1,  44 13  1,  49  8  1, -44 14  2,
              -41 16  2, -29 21  2, -10  0  2,  -7  1  2,  -6  0  2,  -6  2  2,
                   ( 1212 More NOT listed: see .ckf listing file)
PLAT922_ALERT_1_C wR2 in the CIF and FCF Differ by ..............    -0.0011 Check
```

Alert level G

```
PLAT002_ALERT_2_G Number of Distance or Angle Restraints on AtSite       1106 Note
PLAT003_ALERT_2_G Number of Uiso or U(i,j)  Restrained non-H-Atoms       1136 Report
PLAT007_ALERT_5_G Number of Unrefined Donor-H Atoms .............          16 Report
              H15#    H46#    H69#    H84#    H115#   H146#   H773#   H788#
              H177#   H208#   H223#   H238#   H269#   H284#   H315#   H803#
PLAT083_ALERT_2_G SHELXL Second Parameter in WGHT  Unusually Large     250.00 Why ?
PLAT172_ALERT_4_G The CIF-Embedded .res File Contains DFIX Records          1 Report
PLAT174_ALERT_4_G The CIF-Embedded .res File Contains FLAT Records          1 Report
PLAT175_ALERT_4_G The CIF-Embedded .res File Contains SAME Records          7 Report
PLAT176_ALERT_4_G The CIF-Embedded .res File Contains SADI Records         11 Report
PLAT177_ALERT_4_G The CIF-Embedded .res File Contains DELU Records          1 Report
PLAT178_ALERT_4_G The CIF-Embedded .res File Contains SIMU Records          6 Report
PLAT186_ALERT_4_G The CIF-Embedded .res File Contains ISOR Records          2 Report
PLAT187_ALERT_4_G The CIF-Embedded .res File Contains RIGU Records          2 Report
PLAT188_ALERT_3_G A Non-default SIMU Restraint Value has been used     0.0100 Report
PLAT244_ALERT_4_G Low  MinorResAtom  Ueq as Compared to Neighbours      C1_68 Check
PLAT333_ALERT_2_G Large Aver C6-Ring C-C Dist C5_3     -C10_3    .       1.44 Ang.
PLAT333_ALERT_2_G Large Aver C6-Ring C-C Dist C8_3     -C14_3    .       1.44 Ang.
PLAT333_ALERT_2_G Large Aver C6-Ring C-C Dist C5_10    -C10_10   .       1.44 Ang.
PLAT333_ALERT_2_G Large Aver C6-Ring C-C Dist C8_10    -C14_10   .       1.44 Ang.
PLAT343_ALERT_2_G Unusual sp3      Angle Range in Main Residue for     C10_48 Check
PLAT343_ALERT_2_G Unusual sp3      Angle Range in Main Residue for      C7_56 Check
PLAT343_ALERT_2_G Unusual sp3      Angle Range in Main Residue for     C10_65 Check
PLAT380_ALERT_4_G Incorrectly? Oriented X(sp2)-Methyl Moiety .....     C10_34 Check
PLAT380_ALERT_4_G Incorrectly? Oriented X(sp2)-Methyl Moiety .....      C9_34 Check
PLAT380_ALERT_4_G Incorrectly? Oriented X(sp2)-Methyl Moiety .....      C8_34 Check
PLAT380_ALERT_4_G Incorrectly? Oriented X(sp2)-Methyl Moiety .....      C7_34 Check
PLAT380_ALERT_4_G Incorrectly? Oriented X(sp2)-Methyl Moiety .....      C6_34 Check
```

```
PLAT380_ALERT_4_G Incorrectly? Oriented X(sp2)-Methyl Moiety .....     C10_36 Check
PLAT380_ALERT_4_G Incorrectly? Oriented X(sp2)-Methyl Moiety .....      C9_36 Check
PLAT380_ALERT_4_G Incorrectly? Oriented X(sp2)-Methyl Moiety .....      C7_36 Check
PLAT380_ALERT_4_G Incorrectly? Oriented X(sp2)-Methyl Moiety .....      C6_36 Check
PLAT380_ALERT_4_G Incorrectly? Oriented X(sp2)-Methyl Moiety .....     C10_37 Check
PLAT380_ALERT_4_G Incorrectly? Oriented X(sp2)-Methyl Moiety .....      C9_37 Check
PLAT380_ALERT_4_G Incorrectly? Oriented X(sp2)-Methyl Moiety .....      C8_37 Check
PLAT380_ALERT_4_G Incorrectly? Oriented X(sp2)-Methyl Moiety .....      C7_37 Check
PLAT380_ALERT_4_G Incorrectly? Oriented X(sp2)-Methyl Moiety .....      C6_37 Check
PLAT380_ALERT_4_G Incorrectly? Oriented X(sp2)-Methyl Moiety .....     C10_38 Check
PLAT380_ALERT_4_G Incorrectly? Oriented X(sp2)-Methyl Moiety .....      C9_38 Check
PLAT380_ALERT_4_G Incorrectly? Oriented X(sp2)-Methyl Moiety .....      C7_38 Check
PLAT380_ALERT_4_G Incorrectly? Oriented X(sp2)-Methyl Moiety .....      C6_38 Check
PLAT380_ALERT_4_G Incorrectly? Oriented X(sp2)-Methyl Moiety .....     C10_39 Check
PLAT380_ALERT_4_G Incorrectly? Oriented X(sp2)-Methyl Moiety .....      C9_39 Check
PLAT380_ALERT_4_G Incorrectly? Oriented X(sp2)-Methyl Moiety .....      C8_39 Check
PLAT380_ALERT_4_G Incorrectly? Oriented X(sp2)-Methyl Moiety .....      C7_39 Check
PLAT380_ALERT_4_G Incorrectly? Oriented X(sp2)-Methyl Moiety .....      C6_39 Check
PLAT380_ALERT_4_G Incorrectly? Oriented X(sp2)-Methyl Moiety .....     C10_40 Check
PLAT380_ALERT_4_G Incorrectly? Oriented X(sp2)-Methyl Moiety .....      C9_40 Check
PLAT380_ALERT_4_G Incorrectly? Oriented X(sp2)-Methyl Moiety .....      C8_40 Check
PLAT380_ALERT_4_G Incorrectly? Oriented X(sp2)-Methyl Moiety .....      C7_40 Check
PLAT380_ALERT_4_G Incorrectly? Oriented X(sp2)-Methyl Moiety .....      C6_40 Check
PLAT380_ALERT_4_G Incorrectly? Oriented X(sp2)-Methyl Moiety .....      C9_35 Check
PLAT380_ALERT_4_G Incorrectly? Oriented X(sp2)-Methyl Moiety .....      C8_35 Check
PLAT380_ALERT_4_G Incorrectly? Oriented X(sp2)-Methyl Moiety .....      C7_35 Check
PLAT380_ALERT_4_G Incorrectly? Oriented X(sp2)-Methyl Moiety .....      C6_35 Check
PLAT380_ALERT_4_G Incorrectly? Oriented X(sp2)-Methyl Moiety .....     C10_41 Check
PLAT380_ALERT_4_G Incorrectly? Oriented X(sp2)-Methyl Moiety .....      C9_41 Check
PLAT380_ALERT_4_G Incorrectly? Oriented X(sp2)-Methyl Moiety .....      C8_41 Check
PLAT380_ALERT_4_G Incorrectly? Oriented X(sp2)-Methyl Moiety .....      C7_41 Check
PLAT380_ALERT_4_G Incorrectly? Oriented X(sp2)-Methyl Moiety .....     C10_43 Check
PLAT380_ALERT_4_G Incorrectly? Oriented X(sp2)-Methyl Moiety .....      C9_43 Check
PLAT380_ALERT_4_G Incorrectly? Oriented X(sp2)-Methyl Moiety .....      C8_43 Check
PLAT380_ALERT_4_G Incorrectly? Oriented X(sp2)-Methyl Moiety .....      C7_43 Check
PLAT380_ALERT_4_G Incorrectly? Oriented X(sp2)-Methyl Moiety .....      C6_43 Check
PLAT380_ALERT_4_G Incorrectly? Oriented X(sp2)-Methyl Moiety .....     C10_44 Check
PLAT380_ALERT_4_G Incorrectly? Oriented X(sp2)-Methyl Moiety .....      C9_44 Check
PLAT380_ALERT_4_G Incorrectly? Oriented X(sp2)-Methyl Moiety .....      C8_44 Check
PLAT380_ALERT_4_G Incorrectly? Oriented X(sp2)-Methyl Moiety .....      C7_44 Check
PLAT380_ALERT_4_G Incorrectly? Oriented X(sp2)-Methyl Moiety .....      C6_44 Check
PLAT380_ALERT_4_G Incorrectly? Oriented X(sp2)-Methyl Moiety .....      C9_48 Check
PLAT380_ALERT_4_G Incorrectly? Oriented X(sp2)-Methyl Moiety .....      C8_48 Check
PLAT380_ALERT_4_G Incorrectly? Oriented X(sp2)-Methyl Moiety .....      C7_48 Check
PLAT380_ALERT_4_G Incorrectly? Oriented X(sp2)-Methyl Moiety .....      C6_48 Check
PLAT380_ALERT_4_G Incorrectly? Oriented X(sp2)-Methyl Moiety .....     C10_49 Check
PLAT380_ALERT_4_G Incorrectly? Oriented X(sp2)-Methyl Moiety .....      C8_49 Check
PLAT380_ALERT_4_G Incorrectly? Oriented X(sp2)-Methyl Moiety .....      C7_49 Check
PLAT380_ALERT_4_G Incorrectly? Oriented X(sp2)-Methyl Moiety .....      C6_49 Check
PLAT380_ALERT_4_G Incorrectly? Oriented X(sp2)-Methyl Moiety .....     C10_52 Check
PLAT380_ALERT_4_G Incorrectly? Oriented X(sp2)-Methyl Moiety .....      C9_52 Check
PLAT380_ALERT_4_G Incorrectly? Oriented X(sp2)-Methyl Moiety .....      C8_52 Check
PLAT380_ALERT_4_G Incorrectly? Oriented X(sp2)-Methyl Moiety .....      C7_52 Check
PLAT380_ALERT_4_G Incorrectly? Oriented X(sp2)-Methyl Moiety .....      C6_52 Check
PLAT380_ALERT_4_G Incorrectly? Oriented X(sp2)-Methyl Moiety .....     C10_53 Check
PLAT380_ALERT_4_G Incorrectly? Oriented X(sp2)-Methyl Moiety .....      C9_53 Check
PLAT380_ALERT_4_G Incorrectly? Oriented X(sp2)-Methyl Moiety .....      C8_53 Check
```

```
PLAT380_ALERT_4_G Incorrectly? Oriented X(sp2)-Methyl Moiety .....      C7_53 Check
PLAT380_ALERT_4_G Incorrectly? Oriented X(sp2)-Methyl Moiety .....      C9_54 Check
PLAT380_ALERT_4_G Incorrectly? Oriented X(sp2)-Methyl Moiety .....      C8_54 Check
PLAT380_ALERT_4_G Incorrectly? Oriented X(sp2)-Methyl Moiety .....      C7_54 Check
PLAT380_ALERT_4_G Incorrectly? Oriented X(sp2)-Methyl Moiety .....      C6_54 Check
PLAT380_ALERT_4_G Incorrectly? Oriented X(sp2)-Methyl Moiety .....     C10_55 Check
PLAT380_ALERT_4_G Incorrectly? Oriented X(sp2)-Methyl Moiety .....      C9_55 Check
PLAT380_ALERT_4_G Incorrectly? Oriented X(sp2)-Methyl Moiety .....      C7_55 Check
PLAT380_ALERT_4_G Incorrectly? Oriented X(sp2)-Methyl Moiety .....      C6_55 Check
PLAT380_ALERT_4_G Incorrectly? Oriented X(sp2)-Methyl Moiety .....     C10_46 Check
PLAT380_ALERT_4_G Incorrectly? Oriented X(sp2)-Methyl Moiety .....      C9_46 Check
PLAT380_ALERT_4_G Incorrectly? Oriented X(sp2)-Methyl Moiety .....      C8_46 Check
PLAT380_ALERT_4_G Incorrectly? Oriented X(sp2)-Methyl Moiety .....      C7_46 Check
PLAT380_ALERT_4_G Incorrectly? Oriented X(sp2)-Methyl Moiety .....      C6_46 Check
PLAT380_ALERT_4_G Incorrectly? Oriented X(sp2)-Methyl Moiety .....     C10_47 Check
PLAT380_ALERT_4_G Incorrectly? Oriented X(sp2)-Methyl Moiety .....      C9_47 Check
PLAT380_ALERT_4_G Incorrectly? Oriented X(sp2)-Methyl Moiety .....      C8_47 Check
PLAT380_ALERT_4_G Incorrectly? Oriented X(sp2)-Methyl Moiety .....      C7_47 Check
PLAT380_ALERT_4_G Incorrectly? Oriented X(sp2)-Methyl Moiety .....      C6_47 Check
PLAT380_ALERT_4_G Incorrectly? Oriented X(sp2)-Methyl Moiety .....     C10_50 Check
PLAT380_ALERT_4_G Incorrectly? Oriented X(sp2)-Methyl Moiety .....      C9_50 Check
PLAT380_ALERT_4_G Incorrectly? Oriented X(sp2)-Methyl Moiety .....      C8_50 Check
PLAT380_ALERT_4_G Incorrectly? Oriented X(sp2)-Methyl Moiety .....      C6_50 Check
PLAT380_ALERT_4_G Incorrectly? Oriented X(sp2)-Methyl Moiety .....     C10_51 Check
PLAT380_ALERT_4_G Incorrectly? Oriented X(sp2)-Methyl Moiety .....      C9_51 Check
PLAT380_ALERT_4_G Incorrectly? Oriented X(sp2)-Methyl Moiety .....      C8_51 Check
PLAT380_ALERT_4_G Incorrectly? Oriented X(sp2)-Methyl Moiety .....      C7_51 Check
PLAT380_ALERT_4_G Incorrectly? Oriented X(sp2)-Methyl Moiety .....      C6_51 Check
PLAT380_ALERT_4_G Incorrectly? Oriented X(sp2)-Methyl Moiety .....     C10_56 Check
PLAT380_ALERT_4_G Incorrectly? Oriented X(sp2)-Methyl Moiety .....      C9_56 Check
PLAT380_ALERT_4_G Incorrectly? Oriented X(sp2)-Methyl Moiety .....      C8_56 Check
PLAT380_ALERT_4_G Incorrectly? Oriented X(sp2)-Methyl Moiety .....      C6_56 Check
PLAT380_ALERT_4_G Incorrectly? Oriented X(sp2)-Methyl Moiety .....     C10_58 Check
PLAT380_ALERT_4_G Incorrectly? Oriented X(sp2)-Methyl Moiety .....      C9_58 Check
PLAT380_ALERT_4_G Incorrectly? Oriented X(sp2)-Methyl Moiety .....      C8_58 Check
PLAT380_ALERT_4_G Incorrectly? Oriented X(sp2)-Methyl Moiety .....      C7_58 Check
PLAT380_ALERT_4_G Incorrectly? Oriented X(sp2)-Methyl Moiety .....      C6_58 Check
PLAT380_ALERT_4_G Incorrectly? Oriented X(sp2)-Methyl Moiety .....     C10_67 Check
PLAT380_ALERT_4_G Incorrectly? Oriented X(sp2)-Methyl Moiety .....      C9_67 Check
PLAT380_ALERT_4_G Incorrectly? Oriented X(sp2)-Methyl Moiety .....      C8_67 Check
PLAT380_ALERT_4_G Incorrectly? Oriented X(sp2)-Methyl Moiety .....      C7_67 Check
PLAT380_ALERT_4_G Incorrectly? Oriented X(sp2)-Methyl Moiety .....      C6_67 Check
PLAT380_ALERT_4_G Incorrectly? Oriented X(sp2)-Methyl Moiety .....      C10_1 Check
PLAT380_ALERT_4_G Incorrectly? Oriented X(sp2)-Methyl Moiety .....       C8_1 Check
PLAT380_ALERT_4_G Incorrectly? Oriented X(sp2)-Methyl Moiety .....       C7_1 Check
PLAT380_ALERT_4_G Incorrectly? Oriented X(sp2)-Methyl Moiety .....       C6_1 Check
PLAT380_ALERT_4_G Incorrectly? Oriented X(sp2)-Methyl Moiety .....     C10_60 Check
PLAT380_ALERT_4_G Incorrectly? Oriented X(sp2)-Methyl Moiety .....      C9_60 Check
PLAT380_ALERT_4_G Incorrectly? Oriented X(sp2)-Methyl Moiety .....      C8_60 Check
PLAT380_ALERT_4_G Incorrectly? Oriented X(sp2)-Methyl Moiety .....      C6_60 Check
PLAT380_ALERT_4_G Incorrectly? Oriented X(sp2)-Methyl Moiety .....     C10_61 Check
PLAT380_ALERT_4_G Incorrectly? Oriented X(sp2)-Methyl Moiety .....      C8_61 Check
PLAT380_ALERT_4_G Incorrectly? Oriented X(sp2)-Methyl Moiety .....      C7_61 Check
PLAT380_ALERT_4_G Incorrectly? Oriented X(sp2)-Methyl Moiety .....      C6_61 Check
PLAT380_ALERT_4_G Incorrectly? Oriented X(sp2)-Methyl Moiety .....     C10_62 Check
PLAT380_ALERT_4_G Incorrectly? Oriented X(sp2)-Methyl Moiety .....      C9_62 Check
PLAT380_ALERT_4_G Incorrectly? Oriented X(sp2)-Methyl Moiety .....      C8_62 Check
```

```
PLAT380_ALERT_4_G Incorrectly? Oriented X(sp2)-Methyl Moiety .....      C7_62 Check
PLAT380_ALERT_4_G Incorrectly? Oriented X(sp2)-Methyl Moiety .....      C6_62 Check
PLAT380_ALERT_4_G Incorrectly? Oriented X(sp2)-Methyl Moiety .....     C10_63 Check
PLAT380_ALERT_4_G Incorrectly? Oriented X(sp2)-Methyl Moiety .....      C9_63 Check
PLAT380_ALERT_4_G Incorrectly? Oriented X(sp2)-Methyl Moiety .....      C7_63 Check
PLAT380_ALERT_4_G Incorrectly? Oriented X(sp2)-Methyl Moiety .....      C6_63 Check
PLAT380_ALERT_4_G Incorrectly? Oriented X(sp2)-Methyl Moiety .....     C10_64 Check
PLAT380_ALERT_4_G Incorrectly? Oriented X(sp2)-Methyl Moiety .....      C9_64 Check
PLAT380_ALERT_4_G Incorrectly? Oriented X(sp2)-Methyl Moiety .....      C8_64 Check
PLAT380_ALERT_4_G Incorrectly? Oriented X(sp2)-Methyl Moiety .....      C7_64 Check
PLAT380_ALERT_4_G Incorrectly? Oriented X(sp2)-Methyl Moiety .....      C6_64 Check
PLAT380_ALERT_4_G Incorrectly? Oriented X(sp2)-Methyl Moiety .....      C9_65 Check
PLAT380_ALERT_4_G Incorrectly? Oriented X(sp2)-Methyl Moiety .....      C8_65 Check
PLAT380_ALERT_4_G Incorrectly? Oriented X(sp2)-Methyl Moiety .....      C7_65 Check
PLAT380_ALERT_4_G Incorrectly? Oriented X(sp2)-Methyl Moiety .....      C6_65 Check
PLAT380_ALERT_4_G Incorrectly? Oriented X(sp2)-Methyl Moiety .....     C10_66 Check
PLAT380_ALERT_4_G Incorrectly? Oriented X(sp2)-Methyl Moiety .....      C9_66 Check
PLAT380_ALERT_4_G Incorrectly? Oriented X(sp2)-Methyl Moiety .....      C8_66 Check
PLAT380_ALERT_4_G Incorrectly? Oriented X(sp2)-Methyl Moiety .....      C7_66 Check
PLAT380_ALERT_4_G Incorrectly? Oriented X(sp2)-Methyl Moiety .....      C6_66 Check
PLAT380_ALERT_4_G Incorrectly? Oriented X(sp2)-Methyl Moiety .....     C10_70 Check
PLAT380_ALERT_4_G Incorrectly? Oriented X(sp2)-Methyl Moiety .....      C9_70 Check
PLAT380_ALERT_4_G Incorrectly? Oriented X(sp2)-Methyl Moiety .....      C7_70 Check
PLAT380_ALERT_4_G Incorrectly? Oriented X(sp2)-Methyl Moiety .....      C6_70 Check
PLAT606_ALERT_4_G Solvent Accessible VOID(S) in  Crystal Structure          ! Info
PLAT790_ALERT_4_G Centre of Gravity not Within Unit-Cell: Resd.  #          5 Note
              C F3 O3 S
PLAT790_ALERT_4_G Centre of Gravity not Within Unit-Cell: Resd.  #          6 Note
              C F3 O3 S
PLAT790_ALERT_4_G Centre of Gravity not Within Unit-Cell: Resd.  #          7 Note
              C F3 O3 S
PLAT790_ALERT_4_G Centre of Gravity not Within Unit-Cell: Resd.  #          8 Note
              C F3 O3 S
PLAT860_ALERT_3_G Number of Least-Squares Restraints ............     142629 Note
PLAT869_ALERT_4_G ALERTS Related to the Use of SQUEEZE  Suppressed          ! Info
PLAT870_ALERT_4_G ALERTS Related to Twinning Effects Suppressed ..          ! Info
PLAT883_ALERT_1_G Absent Datum for _atom_sites_solution_primary ..     Please Do !
PLAT913_ALERT_3_G Missing # of Very Strong Reflections in FCF ....          3 Note
               -4  0  2,   2  0  2,  -6  0  6,
PLAT933_ALERT_2_G Number of HKL-OMIT Records in Embedded .res File        136 Note
              -19  0 23, -17  0 12, -17  0 19, -16  0  6, -16  0 18, -14 -2 12,
              -14  0  6, -14  2 12, -13  2 20, -11  0  5, -11  0  6, -11  0 15,
              -11  3  4, -10  0  2, -10  0  4,  -9  0  7,  -9  0  8,  -7 -4  2,
               -7 -2  3,  -7 -1  2,  -7  0  1,  -7  0  3,  -7  0  4,  -7  0  6,
               -7  1  2,  -7  2  3,  -6 -4  2,  -6 -2  1,  -6 -2  2,  -6 -1  9,
               -6  1  5,  -6  2  1,  -6  2  2,  -6  4  2,  -5 -3  5,  -5 -1  3,
               -5  0  7,  -5  1  3,  -4 -5  8,  -4 -3  4,  -4 -2  8,  -4 -1  6,
               -4 -1 13,  -4  0  2,  -4  0  6,  -4  1  6,  -4  1 13,  -4  3  4,
               -4  5  8,  -3 -4  7,
                    (   86 More NOT listed: see .ckf listing file)
PLAT941_ALERT_3_G Average HKL Measurement Multiplicity ..........        4.2 Low
PLAT969_ALERT_5_G The 'Henn et al.' R-Factor-gap value ..........      2.840 Note
              Predicted wR2: Based on SigI**2  8.19 or SHELX Weight 21.24
PLAT992_ALERT_5_G Repd & Actual _reflns_number_gt Values Differ by          2 Check
PLAT994_ALERT_1_G SHELXL .ins Contains no or MERG 0 Instruction ..          ! Note
```

```
  0 ALERT level A = Most likely a serious problem - resolve or explain
  2 ALERT level B = A potentially serious problem, consider carefully
 97 ALERT level C = Check. Ensure it is not caused by an omission or oversight
179 ALERT level G = General information/check it is not something unexpected

  6 ALERT type 1 CIF construction/syntax error, inconsistent or missing data
 99 ALERT type 2 Indicator that the structure model may be wrong or deficient
 10 ALERT type 3 Indicator that the structure quality may be low
160 ALERT type 4 Improvement, methodology, query or suggestion
  3 ALERT type 5 Informative message, check
```

It is advisable to attempt to resolve as many as possible of the alerts in all categories. Often the minor alerts point to easily fixed oversights, errors and omissions in your CIF or refinement strategy, so attention to these fine details can be worthwhile. It is up to the individual to critically assess their own results and, if necessary, seek expert advice.

PLATON version of 15/01/2026; check.def file version of 02/01/2026

---

duplicate check

No duplication found

Datablock cu_dll_w6405_0m_sq_sq - ellipsoid plot

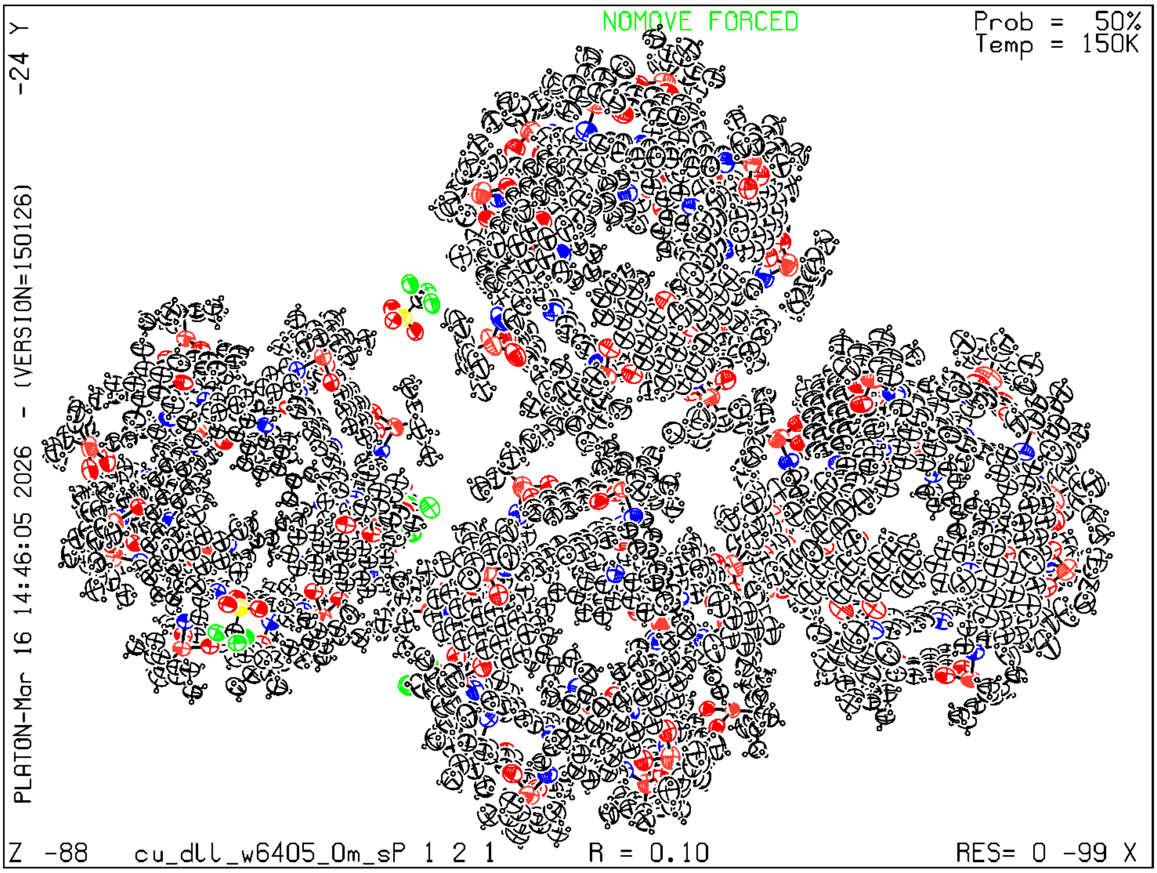

Supplement: Supplementary file 1 [file nwag432_supplemental_files.zip › checkcif-3.pdf]
