# Supplementary material for: Efficient synthesis of complex supramolecular topologies via coordination angle modulation
Source: Natl Sci Rev. 2026 Jul 13;13(18):nwag432. doi: 10.1093/nsr/nwag432 (PMC13629236; doi:10.1093/nsr/nwag432)

checkCIF/PLATON report

Structure factors have been supplied for datablock(s) r-3c

THIS REPORT IS FOR GUIDANCE ONLY. IF USED AS PART OF A REVIEW PROCEDURE FOR PUBLICATION, IT SHOULD NOT REPLACE THE EXPERTISE OF AN EXPERIENCED CRYSTALLOGRAPHIC REFEREE.

No syntax errors found. CIF dictionary Interpreting this report

Datablock: r-3c

Bond precision:	C-C = 0.0256 A		Wavelength=1.54178
Cell:	a=32.8386(8)	b=32.8386(8)	c=137.726(5)
	alpha=90	beta=90	gamma=120
Temperature:	150 K		

	Calculated	Reported
Volume	128622(8)	128622(8)
Space group	R -3 c	R-3c
Hall group	-R 3 2"c	-R 3 2"c
Moiety formula	C112 H98 N10 O12 Rh4 [+ solvent]	C112 H98 N10 O12 Rh4, 6[CF3SO3]
Sum formula	C112 H98 N10 O12 Rh4 [+ solvent]	C118 H98 F18 N10 O30 Rh4 S6
Mr	2187.65	3082.06
Dx,g cm-3	1.017	1.432
Z	36	36
Mu (mm-1)	4.050	5.304
F000	40176.0	55944.0
F000’	40292.90	
h,k,lmax	33,33,138	32,31,136
Nref	15421	14801
Tmin,Tmax	0.398,0.451	0.513,0.750
Tmin’	0.301	

```
Correction method= # Reported T Limits: Tmin=0.513 Tmax=0.750
AbsCorr = NONE

Data completeness= 0.960            Theta(max)= 51.089

R(reflections)= 0.1545( 5459)       wR2(reflections)= 0.3357( 14801)

S = 1.070                  Npar= 1101
```

The following ALERTS were generated. Each ALERT has the format
test-name_ALERT_alert-type_alert-level.
Click on the hyperlinks for more details of the test.

Alert level A

```
THETM01_ALERT_3_A  The value of sine(theta_max)/wavelength is less than 0.550
            Calculated sin(theta_max)/wavelength =    0.5047
```

Author Response: This is due to the poor diffraction of the crystal.

Alert level B

```
PLAT026_ALERT_3_B Ratio Observed / Unique Reflections (too) Low ..        37% Check
```

Author Response: This is due to the poor diffraction of the crystal.

```
PLAT342_ALERT_3_B Low Bond Precision on  C-C Bonds ..............    0.02564 Ang.
```

Author Response: Poor data quality in context with large assymetric unit and heavy rhodium atoms in the lattice.

Alert level C

```
PLAT029_ALERT_3_C _diffrn_measured_fraction_theta_full value Low .      0.960 Why?
PLAT057_ALERT_3_C Correction for Absorption Required   RT(exp) ...       1.13 Do !
PLAT082_ALERT_2_C High R1 Value ..................................       0.15 Report
PLAT084_ALERT_3_C High wR2 Value (i.e. > 0.25) ...................       0.34 Report
PLAT234_ALERT_4_C Large Hirshfeld Difference Rh4      --O8       .       0.17 Ang.
PLAT242_ALERT_2_C Low   MainResAtom  Ueq as Compared to Neighbours        Rh1 Check
PLAT242_ALERT_2_C Low   MainResAtom  Ueq as Compared to Neighbours         O4 Check
PLAT242_ALERT_2_C Low   MainResAtom  Ueq as Compared to Neighbours         O8 Check
PLAT242_ALERT_2_C Low   MainResAtom  Ueq as Compared to Neighbours         O9 Check
PLAT242_ALERT_2_C Low   MainResAtom  Ueq as Compared to Neighbours         N1 Check
PLAT242_ALERT_2_C Low   MainResAtom  Ueq as Compared to Neighbours         N9 Check
PLAT260_ALERT_2_C Large Average Ueq of Residue Including       Rh1      0.229 Check
PLAT309_ALERT_2_C Single Bonded Oxygen (C-O > 1.3 Ang) ..........         O3 Check
PLAT309_ALERT_2_C Single Bonded Oxygen (C-O > 1.3 Ang) ..........        O10 Check
```

```
PLAT369_ALERT_2_C Long   C(sp2)-C(sp2) Bond  C15      - C67      .       1.54 Ang.
PLAT369_ALERT_2_C Long   C(sp2)-C(sp2) Bond  C56      - C60      .       1.53 Ang.
PLAT410_ALERT_2_C Short Intra H...H Contact  H57      ..H61      .       1.96 Ang.
                                                   x,y,z  =      1_555 Check
PLAT412_ALERT_2_C Short Intra XH3 .. XHn     H78A     ..H79A     .       1.80 Ang.
                                                   x,y,z  =      1_555 Check
PLAT413_ALERT_2_C Short Inter XH3 .. XHn     H9       ..H11H     .       2.04 Ang.
                                          2/3+y,1/3+x,5/6-z  =     10_555 Check
PLAT413_ALERT_2_C Short Inter XH3 .. XHn     H10      ..H11H     .       2.11 Ang.
                                          2/3+y,1/3+x,5/6-z  =     10_555 Check
PLAT413_ALERT_2_C Short Inter XH3 .. XHn     H10      ..H11I     .       2.11 Ang.
                                          2/3+y,1/3+x,5/6-z  =     10_555 Check
PLAT413_ALERT_2_C Short Inter XH3 .. XHn     H11D     ..H92B     .       2.03 Ang.
                                        -1/3+y,-2/3+x,5/6-z  =     10_445 Check
PLAT413_ALERT_2_C Short Inter XH3 .. XHn     H11I     ..H92A     .       2.13 Ang.
                                        -1/3+y,-2/3+x,5/6-z  =     10_445 Check
PLAT413_ALERT_2_C Short Inter XH3 .. XHn     H11I     ..H92C     .       2.13 Ang.
                                        -1/3+y,-2/3+x,5/6-z  =     10_445 Check
PLAT413_ALERT_2_C Short Inter XH3 .. XHn     H19      ..H99B     .       2.05 Ang.
                                        2/3-x,1/3-x+y,5/6-z  =     12_555 Check
PLAT413_ALERT_2_C Short Inter XH3 .. XHn     H19      ..H99C     .       2.14 Ang.
                                        2/3-x,1/3-x+y,5/6-z  =     12_555 Check
PLAT420_ALERT_2_C D-H Bond Without Acceptor  N2       --H2A      .    Please Check
PLAT420_ALERT_2_C D-H Bond Without Acceptor  N8       --H8       .    Please Check
PLAT905_ALERT_3_C Negative K value in the Analysis of Variance ...    -56.893 Report
PLAT905_ALERT_3_C Negative K value in the Analysis of Variance ...     -7.346 Report
PLAT905_ALERT_3_C Negative K value in the Analysis of Variance ...     -0.562 Report
PLAT911_ALERT_3_C Missing FCF Refl Between Thmin & STh/L=    0.505        608 Report
               -2  4  0,  -4  8  0,  -8 16  0,  -1 29  0,  -3 30  0, -15 33  0,
               -5  6  1,  -2  6  1,  -1  7  1,  -6  8  1,  -1 10  1, -27 29  1,
              -26 30  1, -25 31  1, -14 33  1,  -2  2  2,  -2  5  2,  -6  7  2,
                0  7  2,  -7  9  2,   0 28  2,  -2 29  2,  -4 30  2,  -6 31  2,
              -23 32  2, -19 33  2,  -1  5  3,  -3  6  3,  -5  7  3,  -5 10  3,
                   (  578 More NOT listed: see .ckf listing file)
PLAT977_ALERT_2_C Check Negative Difference Density on H79B      .      -0.36 eA-3
PLAT977_ALERT_2_C Check Negative Difference Density on H79C      .      -0.34 eA-3
PLAT977_ALERT_2_C Check Negative Difference Density on H99A      .      -0.34 eA-3
```

Alert level G

```
FORMU01_ALERT_2_G  There is a discrepancy between the atom counts in the
            _chemical_formula_sum and the formula from the _atom_site* data.
            Atom count from _chemical_formula_sum:C118 H98 F18 N10 O30 Rh4 S6
            Atom count from the _atom_site data:  C112 H98 N10 O12 Rh4
CELLZ01_ALERT_1_G Difference between formula and atom_site contents detected.
CELLZ01_ALERT_1_G ALERT: Large difference may be due to a
            symmetry error - see SYMMG tests
           From the CIF: _cell_formula_units_Z   36
           From the CIF: _chemical_formula_sum  C118 H98 F18 N10 O30 Rh4 S6
           TEST: Compare cell contents of formula and atom_site data

           atom    Z*formula  cif sites diff
           C       4248.00   4032.00  216.00
           H       3528.00   3528.00    0.00
           F        648.00      0.00  648.00
           N        360.00    360.00    0.00
           O       1080.00    432.00  648.00
```

```
          Rh       144.00    144.00    0.00
          S        216.00      0.00  216.00
PLAT002_ALERT_2_G Number of Distance or Angle Restraints on AtSite         67 Note
PLAT003_ALERT_2_G Number of Uiso or U(i,j)  Restrained non-H-Atoms        138 Report
PLAT007_ALERT_5_G Number of Unrefined Donor-H Atoms .............           2 Report
              H2A     H8
PLAT041_ALERT_1_G Calc. and Reported SumFormula    Strings  Differ     Please Check
              Calc: C112 H98 N10 O12 Rh4
              Rep.: C118 H98 F18 N10 O30 Rh4 S6
PLAT042_ALERT_1_G Calc. and Reported MoietyFormula Strings  Differ     Please Check
              Calc: C112 H98 N10 O12 Rh4
              Rep.: C112 H98 N10 O12 Rh4, 6[CF3SO3]
PLAT051_ALERT_1_G Mu(calc) and Mu(cif) Ratio Differs from 1.0 by .      23.64 %
PLAT083_ALERT_2_G SHELXL Second Parameter in WGHT  Unusually Large    3880.00 Why ?
PLAT172_ALERT_4_G The CIF-Embedded .res File Contains DFIX Records         38 Report
PLAT173_ALERT_4_G The CIF-Embedded .res File Contains DANG Records         16 Report
PLAT174_ALERT_4_G The CIF-Embedded .res File Contains FLAT Records          4 Report
PLAT176_ALERT_4_G The CIF-Embedded .res File Contains SADI Records         16 Report
PLAT177_ALERT_4_G The CIF-Embedded .res File Contains DELU Records          1 Report
PLAT178_ALERT_4_G The CIF-Embedded .res File Contains SIMU Records          5 Report
PLAT187_ALERT_4_G The CIF-Embedded .res File Contains RIGU Records          4 Report
PLAT188_ALERT_3_G A Non-default SIMU Restraint Value has been used     0.0100 Report
PLAT191_ALERT_3_G A Non-default SADI Restraint Value has been used     0.0400 Report
PLAT191_ALERT_3_G A Non-default SADI Restraint Value has been used     0.0400 Report
PLAT191_ALERT_3_G A Non-default SADI Restraint Value has been used     0.0400 Report
PLAT191_ALERT_3_G A Non-default SADI Restraint Value has been used     0.0400 Report
PLAT191_ALERT_3_G A Non-default SADI Restraint Value has been used     0.0400 Report
PLAT191_ALERT_3_G A Non-default SADI Restraint Value has been used     0.0400 Report
PLAT191_ALERT_3_G A Non-default SADI Restraint Value has been used     0.0400 Report
PLAT191_ALERT_3_G A Non-default SADI Restraint Value has been used     0.0400 Report
PLAT380_ALERT_4_G Incorrectly? Oriented X(sp2)-Methyl Moiety .....        C82 Check
PLAT380_ALERT_4_G Incorrectly? Oriented X(sp2)-Methyl Moiety .....        C81 Check
PLAT380_ALERT_4_G Incorrectly? Oriented X(sp2)-Methyl Moiety .....        C80 Check
PLAT380_ALERT_4_G Incorrectly? Oriented X(sp2)-Methyl Moiety .....        C79 Check
PLAT380_ALERT_4_G Incorrectly? Oriented X(sp2)-Methyl Moiety .....        C78 Check
PLAT380_ALERT_4_G Incorrectly? Oriented X(sp2)-Methyl Moiety .....        C91 Check
PLAT380_ALERT_4_G Incorrectly? Oriented X(sp2)-Methyl Moiety .....        C89 Check
PLAT380_ALERT_4_G Incorrectly? Oriented X(sp2)-Methyl Moiety .....       C102 Check
PLAT380_ALERT_4_G Incorrectly? Oriented X(sp2)-Methyl Moiety .....       C108 Check
PLAT432_ALERT_2_G Short Inter X...Y Contact  O3        ..C112     .       2.64 Ang.
                                  2/3-x,1/3-x+y,5/6-z  =     12_555 Check
PLAT432_ALERT_2_G Short Inter X...Y Contact  O11       ..C90      .       2.30 Ang.
                                        y,1-x+y,1-z  =     20_566 Check
PLAT432_ALERT_2_G Short Inter X...Y Contact  C9        ..C112     .       2.70 Ang.
                                  2/3+y,1/3+x,5/6-z  =     10_555 Check
PLAT432_ALERT_2_G Short Inter X...Y Contact  C10       ..C112     .       2.67 Ang.
                                  2/3+y,1/3+x,5/6-z  =     10_555 Check
PLAT432_ALERT_2_G Short Inter X...Y Contact  C92       ..C111     .       2.90 Ang.
                                  2/3+y,1/3+x,5/6-z  =     10_555 Check
PLAT432_ALERT_2_G Short Inter X...Y Contact  C92       ..C104     .       3.01 Ang.
                                  2/3+y,1/3+x,5/6-z  =     10_555 Check
PLAT432_ALERT_2_G Short Inter X...Y Contact  C92       ..C112     .       3.15 Ang.
                                  2/3+y,1/3+x,5/6-z  =     10_555 Check
PLAT606_ALERT_4_G Solvent Accessible VOID(S) in  Crystal Structure          ! Info
PLAT860_ALERT_3_G Number of Least-Squares Restraints ............        2178 Note
PLAT868_ALERT_4_G ALERTS Due to the Use of _smtbx_masks Suppressed          ! Info
PLAT910_ALERT_3_G Missing FCF Reflection(s) Below Theta(Min)[Deg]=       2.69 Note
```

```
               0  1  2,  -1  1  4,   0  0  6,
PLAT913_ALERT_3_G Missing # of Very Strong Reflections in FCF ....          3 Note
              -2  2  2,   0  1  8,   0  0 18,
PLAT933_ALERT_2_G Number of HKL-OMIT Records in Embedded .res File        165 Note
             -10 10  4, -10 13 19, -10 13 37, -10 14  6,  -9  9 42,  -9 11 28,
              -9 11 37,  -8 10  9,  -8 10 42,  -8 11 11,  -8 12  4,  -8 14  5,
              -8 15 31,  -8 16  0,  -7  7 10,  -7  7 28,  -7  7 58,  -7  8 39,
              -7  9  2,  -7  9 38,  -7 10 40,  -7 10 43,  -7 11 15,  -7 11 42,
              -7 16  7,  -7 16 25,  -6  7  2,  -6  7  8,  -6  7 14,  -6  8  1,
              -6  8 10,  -6  9 12,  -6  9 36,  -6  9 45,  -6 10 11,  -6 10 20,
              -6 11 28,  -6 13 20,  -6 13 29,  -5  5 26,  -5  6  1,  -5  6 10,
              -5  6 25,  -5  7  3,  -5  7 21,  -5  7 30,  -5  7 36,  -5  8 14,
              -5  9 25,  -5 10  3,
                  (  115 More NOT listed: see .ckf listing file)
PLAT951_ALERT_5_G Calculated (ThMax) and CIF-Reported Kmax Differ           2 Units
PLAT952_ALERT_5_G Calculated (ThMax) and CIF-Reported Lmax Differ.          2 Units
PLAT958_ALERT_1_G Calculated (ThMax) and Actual (FCF) Lmax Differ.          2 Units
PLAT969_ALERT_5_G The 'Henn et al.' R-Factor-gap value ..........       3.990 Note
              Predicted wR2: Based on SigI**2  8.41 or SHELX Weight 31.37
PLAT978_ALERT_2_G Number C-C Bonds with Positive Residual Density.          0 Info
PLAT994_ALERT_1_G SHELXL .ins Contains no or MERG 0 Instruction ..          ! Note
```

```
  1 ALERT level A = Most likely a serious problem - resolve or explain
  2 ALERT level B = A potentially serious problem, consider carefully
 35 ALERT level C = Check. Ensure it is not caused by an omission or oversight
 54 ALERT level G = General information/check it is not something unexpected

  7 ALERT type 1 CIF construction/syntax error, inconsistent or missing data
 40 ALERT type 2 Indicator that the structure model may be wrong or deficient
 22 ALERT type 3 Indicator that the structure quality may be low
 19 ALERT type 4 Improvement, methodology, query or suggestion
  4 ALERT type 5 Informative message, check
```

It is advisable to attempt to resolve as many as possible of the alerts in all categories. Often the minor alerts point to easily fixed oversights, errors and omissions in your CIF or refinement strategy, so attention to these fine details can be worthwhile. It is up to the individual to critically assess their own results and, if necessary, seek expert advice.

PLATON version of 15/01/2026; check.def file version of 02/01/2026

---

duplicate check

No duplication found

Datablock r-3c - ellipsoid plot

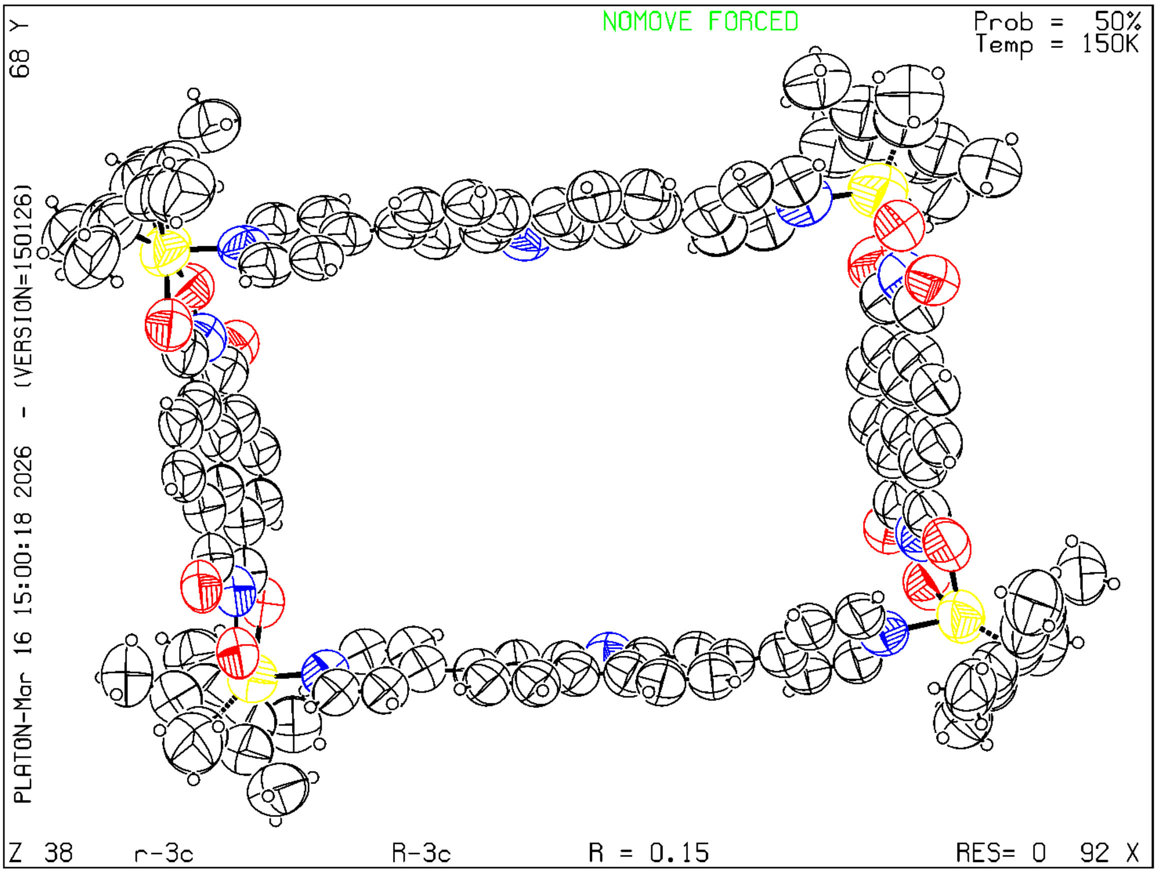

Supplement: Supplementary file 1 [file nwag432_supplemental_files.zip › checkcif-4.pdf]
