# Supplementary material for: Efficient synthesis of complex supramolecular topologies via coordination angle modulation
Source: Natl Sci Rev. 2026 Jul 13;13(18):nwag432. doi: 10.1093/nsr/nwag432 (PMC13629236; doi:10.1093/nsr/nwag432)
Supplement: Supplementary file 1 [file nwag432_supplemental_files.zip › SI-Dang-NSR_MS-2025-2753.R3 .pdf]

Supporting Information

Efficient synthesis of a snowflake-shaped folded assembly, an 8_{18} knot, and a closed three-link chain via coordination angle modulation

Li-Long Dang[1,2,†,*], Xiao-Qian Wan[1,2,†], Jian-Xin Yang[1,†], Mei-Jia Chang[3,*], Francisco Aznarez[1], Ying Zhao[1,*], Feng Bai[2] and Lu-Fang Ma[1]

[1]College of Chemistry and Chemical Engineering, Luoyang Normal University, Henan Province Function-Oriented Porous Materials Key Laboratory, Luoyang 471934, China;

[2]School of Nanoscience and Materials Engineering, Henan University, Kaifeng 475004, China;

[3]School of Environmental Engineering and Chemistry, Luoyang Institute of Science and Technology, Luoyang 471023, China.

*Corresponding author. E-mail: danglilong8@163.com; cmj@mail.nankai.edu.cn; zhaoyingchem@126.com

[†]Equally contributed to this work.

Table of Contents

1. General procedures 3

2. Synthesis and experimental methods 4

3. Single crystal structure details 8

4. NMR Spectra 10

5. Hydrodynamic radii 16

6. ESI-TOF-MS spectra 18

7. Experimental details of photothermal conversion 21

8. Thermogravimetric analysis 30

9. Theoretical arithmetic 31

10. Measurement of photophysical properties 32

11. X-ray crystallography details and experimental details 35

12. References 39

1. General procedures

All reagents and solvents were purchased from commercial sources and used as supplied unless otherwise mentioned. NMR spectra were recorded on Bruker AVANCE I 500 spectrometers at room temperature and referenced to the residual protonated solvent. Proton chemical shifts are reported relative to the solvent residual peak (δ H = 3.31 (CD_3OD))[1]. Coupling constants are expressed in Hertz. Elemental analyses were performed on an Elementar Vario EL III analyzer. ESI-MS spectra were recorded on a Micro TOF II mass spectrometer. EPR signals were measured on an Electronic paramagnetic resonance spectrometer (Magnettech ESR 5000, Bruker, Germany). An infrared thermal imager (testo IRSoft, Germany) was used to record changes in sample surface temperature during the test. UV-Vis-IR reflectance spectra were measured on a UV-Vis-IR (Lambda 950, PE, USA) spectrometer equipped with an integrating sphere. The various ions presented in this paper were measured by inductively coupled plasma mass spectrometry (7580 ICP-MS, Agilent, USA). Using CuKα radiation (λ = 1.54178 Å) at 150 K, a Bruker D8 Venture diffractometer (Bruker AXS GmbH, Germany) was performed to determine the structural parameters of all compounds. Their structures were merged by SHELXL according to the crystal class for the calculation of statistics and refinement[2]. Furthermore, the X-ray crystallographic data for **2**$(OTf)_{12}$, **3**$(OTf)_{16}$ and **4**$(OTf)_{12}$ are shown in Tables S3–S5. Cambridge Crystallographic Data Centre (CCDC) numbers: 2482774 (**2**$(OTf)_{12}$), 2482775 (**3**$(OTf)_{16}$), and 2482776 (**4**$(OTf)_{12}$).

2. Synthesis and experimental methods

2.1 Synthesis of Ligand **L1**

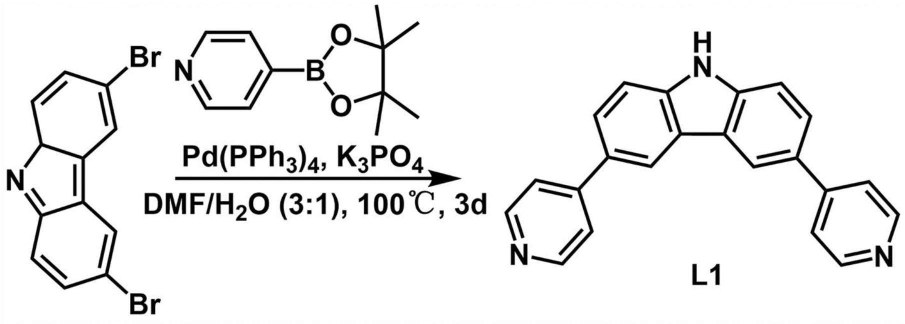

Figure S1. Synthesis of **L1**.

Under an argon atmosphere, 3,6-dibromo-9H-carbazole (100.00 mg, 0.24 mmol, 1.0 eq.), 4-(4,4,5,5-tetramethyl-1,3,2-dioxaborolan-2-yl)pyridine (300.71 mg, 1.47 mmol, 6.0 eq.), $Pd(PPh_3)_4$ (28.24 mg, 0.02 mmol, 0.1 eq), and K_3PO_4 (155.63 mg, 0.73 mmol, 3.0 eq.) were added to a degassed DMF/H_2O mixture (3:1, 30 mL). The mixture was heated at 100 °C in a pressure reactor for 3 days. After cooling to room temperature, the mixture was washed with water, brine, and saturated NH_4Cl solution, dried over $MgSO_4$, and the solvent was removed under reduced pressure. The crude product was purified by column chromatography (pentane/acetone, 1:1) and GPC ($CHCl_3$) to afford yellow solid **L1**.

2.2 Synthesis of Ligand **L2**

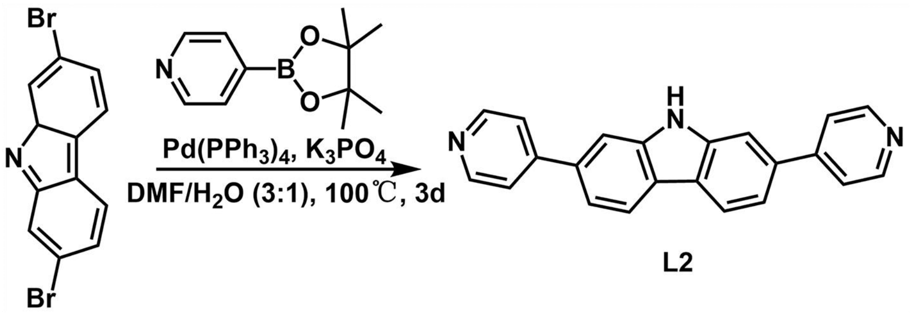

Figure S2. Synthesis of **L2**.

Under an argon atmosphere, 2,7-dibromo-9H-carbazole (100.00 mg, 0.24 mmol, 1.0 eq.), 4-(4,4,5,5-tetramethyl-1,3,2-dioxaborolan-2-yl)pyridine (300.71 mg, 1.47 mmol, 6.0 eq.), $Pd(PPh_3)_4$ (28.24 mg, 0.02 mmol, 0.1 eq.), and K_3PO_4 (155.63 mg, 0.73 mmol, 3.0 eq.) were added to a degassed DMF/H_2O mixture (3:1, 30 mL). The mixture was heated at 100 °C in a pressure reactor

for 3 days. After cooling to room temperature, the mixture was washed with water, brine, and saturated NH_4Cl solution, dried over $MgSO_4$, and the solvent was removed under reduced pressure. The crude product was purified by column chromatography (pentane/acetone, 1:1) and GPC ($CHCl_3$) to afford yellow solid **L2**.

2.3 Synthesis of compound **1**$(OTf)_8$ (molecular-tweezer-like compound)

AgOTf (123.2 mg, 0.48 mmol) and $[Cp^*RhCl_2]_2$ (74.4 mg, 0.12 mmol) were added to a methanol solution (18 mL) at room temperature, the mixture was treated with light protection and stirred in the dark for 12 h, and then the white AgCl precipitate was filtered out to obtain an orange solution. Subsequently, 6,11-Dihydroxy-5,12-naphthacenedione (38.53 mg, 0.12 mmol) and NaOH (9.6 mg, 0.24 mmol) were added to the filtrate, and then the mixed solution was stirred at room temperature for 12 h to give a light green solution, and the solution was treated with ether to obtain the building block **E1**$(OTf)_2$. Adding 3,6-di(pyridin-4-yl)-9H-carbazole (**L1**) (38.56 mg, 0.12 mmol) to a methanol solution of 0.12 mmol **E1**$(OTf)_2$, the mixture was stirred at room temperature for another 12 h to give a green solution. Finally, the single crystals were grown by liquid diffusion, and the nonpolar solvent ether was added to the mixed solution of methanol. The product was recrystallized from a diethyl ether mixture to afford green block-shaped crystals **1**$(OTf)_8$. Crystals of **1**$(OTf)_{12}$ suitable for single-crystal X-ray diffraction were typically obtained within 3 days.

2.4 Synthesis of compound **2**$(OTf)_{12}$ (snowflake-shaped folded assembly)

AgOTf (123.2 mg, 0.48 mmol) and $[Cp^*RhCl_2]_2$ (74.4 mg, 0.12 mmol) were added to a methanol solution (18 mL) at room temperature, the mixture was treated with light protection and stirred in the dark for 12 h, and then the white AgCl precipitate was filtered out to obtain a yellow solution. Subsequently, 2,7-dihydroxybenzo[lmn][3,8]phenanthroline-1,3,6,8(2H,7H)-tetraone (35.78 mg, 0.12 mmol) and NaOH (9.6 mg, 0.24 mmol) were added to the filtrate, and then the mixed solution was stirred at room temperature for 12 h to give a yellow solution, and the solution was treated with ether to obtain the building block **E2**$(OTf)_2$. Adding 3,6-di(pyridin-4-yl)-9H-carbazole (**L1**) (38.56 mg, 0.12 mmol) to a methanol solution of 0.12 mmol **E2**$(OTf)_2$, the mixture was stirred at room temperature for another 12 h to give a dark yellow solution. Finally, the single crystals were grown by liquid diffusion, and the nonpolar solvent ether was added to the mixed solution of methanol. The product was recrystallized from a diethyl ether mixture to afford brown block-shaped crystals **2**$(OTf)_{12}$. Crystals of **2**$(OTf)_{12}$ suitable for single-crystal X-ray diffraction were typically obtained

within 4 days. ^{1}H NMR (500 MHz, CD_3OD, ppm, with respect to Cp*Rh): δ = 8.28 (d, 2H, J = 5.5 Hz, pyridyl-H_a), δ = 7.82 (d, 2H, J = 5.5 Hz, pyridyl-H_b), δ = 7.32 (d, 1H, J = 8.00 Hz, carbazole-H_c), δ = 7.88 (d, 1H, J = 8.00 Hz, carbazole-H_d), δ = 7.67 (s, 1H, carbazole-H_e), δ = 5.71 (s, 2H, naphthyl-H_f), δ = 1.72 (s, 15H, Cp*-H_g).

2.5 Synthesis of compound **3**$(OTf)_{16}$ (8_{18} knot)

In order to synthesize building block **E1**$(OTf)_2$, add AgOTf (123.2 mg, 0.48 mmol) and $[Cp^*RhCl_2]_2$ (74.4 mg, 0.12 mmol) to a methanol solution (18 mL). The mixture was stirred in the dark for 12 h and then filtered at room temperature. Then 6,11-dihydroxytetracene-5,12-dione (34.83 mg, 0.12 mmol) and NaOH (9.6 mg, 0.24 mmol) were added to the filtrate. Then the mixed solution was stirred at room temperature for 12 h to give a green solution, and the solution was treated with ether to obtain the building block **E1**$(OTf)_2$. Adding 2,7-di(pyridin-4-yl)-9H-carbazole (**L2**) (38.56 mg, 0.12 mmol) to a methanol solution of 0.12 mmol **E1**$(OTf)_2$, the mixture was stirred at room temperature for another 12 h to give a dark green solution. Finally, the single crystals were grown by dark purple diffusion, and the nonpolar solvent ether was added to the mixed solution of methanol. The product was recrystallized from a diethyl ether mixture to afford dark green block-shaped crystals **3**$(OTf)_{16}$. Crystals of **3**$(OTf)_{16}$ suitable for single-crystal X-ray diffraction were typically obtained within 5 days. ^{1}H NMR (500 MHz, CD_3OD, ppm, with respect to Cp*Rh): δ = 8.86, 8.78 (d, 2H J = 6.00 Hz, J = 5.50 Hz pyridyl-H_{a1}, H_{a2}), δ = 8.50, 8.25 (d, 2H, J = 6.0 Hz, pyridyl-H_{b1}, H_{b2}), δ = 7.87, 7.79 (d, 1H, J = 9.0 Hz, J = 8.0 Hz carbazole-H_{c1}, H_{c2}), δ = 8.42, 8.38 (d, 1H, J = 8.0 Hz, J = 8.0 Hz carbazole-H_{d1}, H_{d2}), δ = 8.18, 8.09 (s, 1H, carbazole-H_{e1}, H_{e2}), δ = 8.67, (d, 2H, **E1**$(OTf)_2$-H_f), δ = 8.26, (d, 2H, **E1**$(OTf)_2$-H_g), δ = 1.704, 1.697 (s, 15H, Cp*-H_{h1}, H_{h2}).

2.6 Synthesis of compound **4**$(OTf)_{12}$ (closed three-link chain)

The first step was to add AgOTf (123.2 mg, 0.48 mmol) and $[Cp^*RhCl_2]_2$ (74.4 mg, 0.12 mmol) to a methanol solution (18 mL) at room temperature. The solution was stirred in the dark for 12 h and then filtered to remove the AgCl precipitate. Subsequently, 2,7-dihydroxybenzo[lmn][3,8]phenanthroline-1,3,6,8(2H,7H)-tetraone (35.78 mg, 0.12 mmol) and NaOH (9.6 mg, 0.24 mmol) were added to the filtrate, and the mixed solution was stirred at room temperature for 12 h to obtain a brown solution, and the solution was treated with ether to obtain the building block **E2**$(OTf)_2$. 2,7-di(pyridin-4-yl)-9H-carbazole (38.56 mg, 0.12 mmol) was added to the solution of **E2**$(OTf)_2$. Stirring the mixture at room temperature for another 12 hours generated a

dark brown solution. Finally, upon the addition of isopropyl ether, a black solid was precipitated and collected. The product was recrystallized from a CH_3OH/isopropyl ether mixture to afford dark block-shaped crystals **4**$(OTf)_{12}$. Crystals of **4**$(OTf)_{12}$ suitable for single-crystal X-ray diffraction were typically obtained within 4 days. ^1H NMR (500 MHz, CD_3OD, ppm, with respect to Cp*Rh): δ = 9.22, 9.16 (d, 2H J = 4.00 Hz, J = 4.50 Hz pyridyl-H_{a1}, H_{a2}), δ = 7.49, 8.29 (*d*, 2H, J = 5.0 Hz, J = 8.0 Hz, pyridyl-H_{b1}, H_{b2}), δ = 7.90, 7.75 (d, 1H, J = 8.5 Hz, J = 6.5 Hz carbazole-H_{c1}, H_{c2}), δ = 8.72, 8.48 (d, 1H, J = 10.0 Hz, J = 7.0 Hz carbazole-H_{d1}, H_{d2}), δ = 8.36, 7.64 (s, 1H, carbazole-H_{e1}, H_{e2}), δ = 7.00, (d, 2H, **E2**$(OTf)_2$-H_f), δ = 1.94, 1.90 (s, 15H, Cp*-H_{g1}, H_{g2}).

3. Single crystal structure details

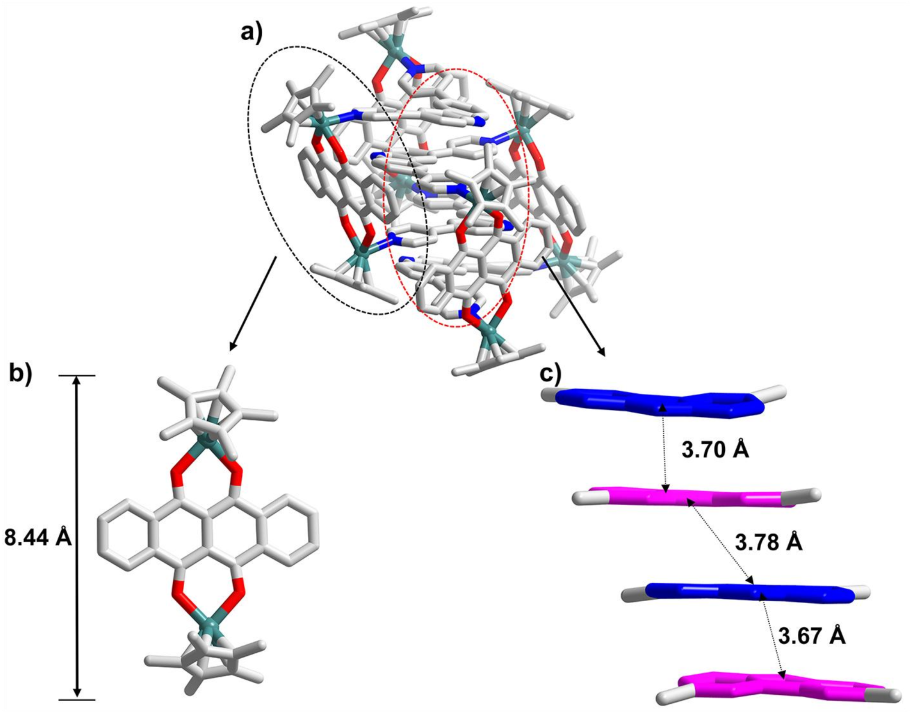

Figure S3. Single crystal details of compound **1**$(OTf)_8$. (a) Side view of the solid-state structure of compound **1**$(OTf)_8$. (b) The distance between Rh···Rh of building block **E1**$(OTf)_2$. (c) The distances between each plane of ligand **L1**. Most hydrogen atoms, anions, and solvent molecules have been omitted for clarity; N, blue; O, red; C, gray; Rh, sea green.

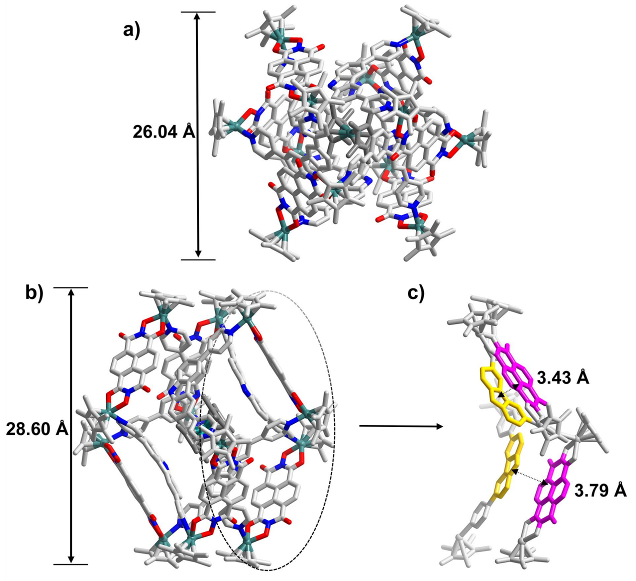

Figure S4. (a) Top view, (b) side view, and (c) local magnification of the π-π stacking interactions in snowflake-shaped folded assembly **2**$(OTf)_{12}$. Most hydrogen atoms, anions, and solvent

molecules have been omitted for clarity; N, blue; O, red; C, gray; Rh, sea green.

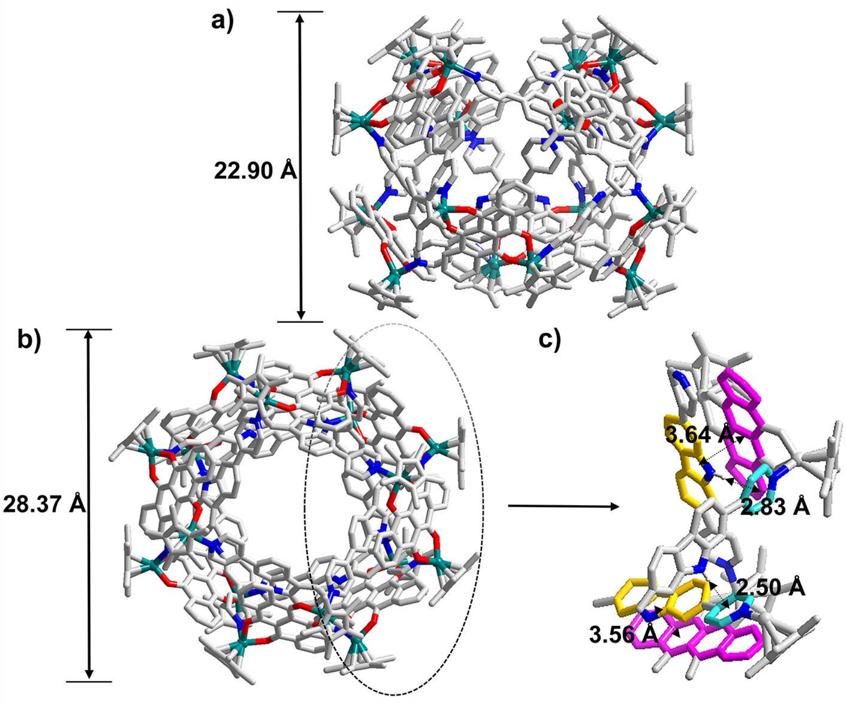

Figure S5. Single crystal details of compound **3**$(OTf)_{16}$. (a) Vertical distance of compound **3**$(OTf)_{16}$. (b) Horizontal distance of compound **3**$(OTf)_{16}$. (c) The distances between carbazole planes and quaterphenylene planes of compound **3**$(OTf)_{16}$. Most hydrogen atoms, anions, and solvent molecules have been omitted for clarity; N, blue; O, red; C, gray; Rh, sea green.

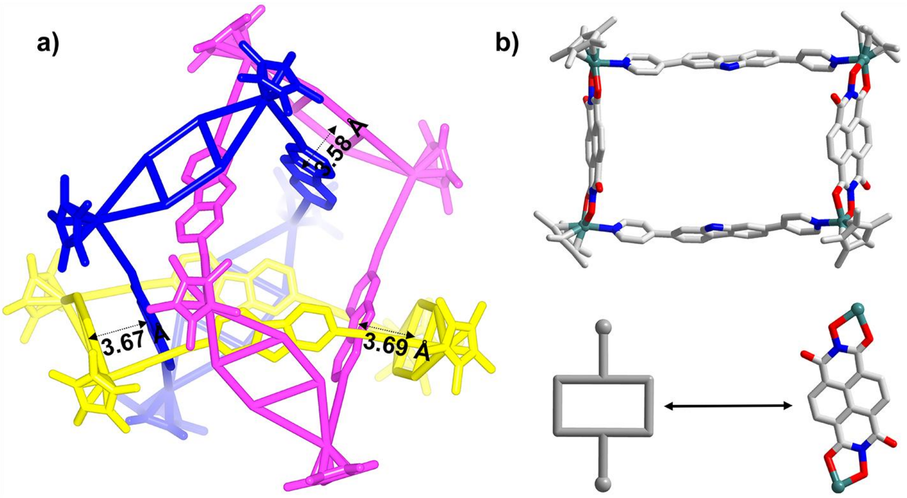

Figure S6. Single crystal details of compound **4**$(OTf)_{12}$. (a) The distances between carbazole planes and biphenyl planes of compound **4**$(OTf)_{12}$. (b) The quadnuclear metallamacrocycle that constitutes compound **4**$(OTf)_{12}$. Most hydrogen atoms, anions, and solvent molecules have been omitted for clarity; N, blue; O, red; C, gray; Rh, sea green.

4. NMR Spectra

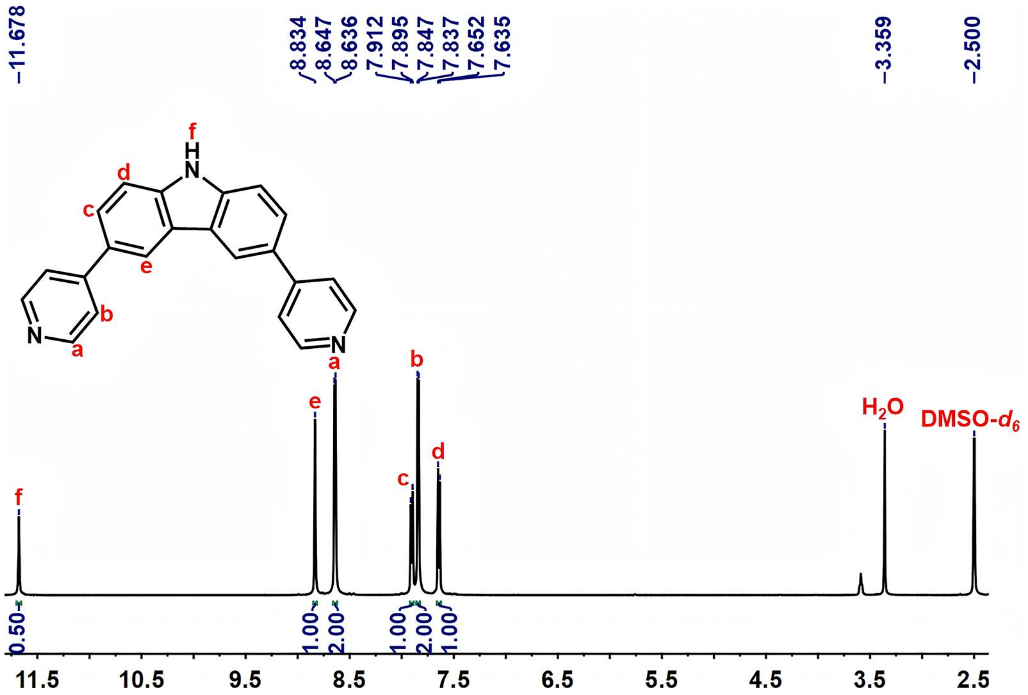

Figure S7. The ^{1}H NMR (500 MHz, DMSO-d_6, ppm) for ligand **L1** (30.0 mM, with respect to Cp*Rh).

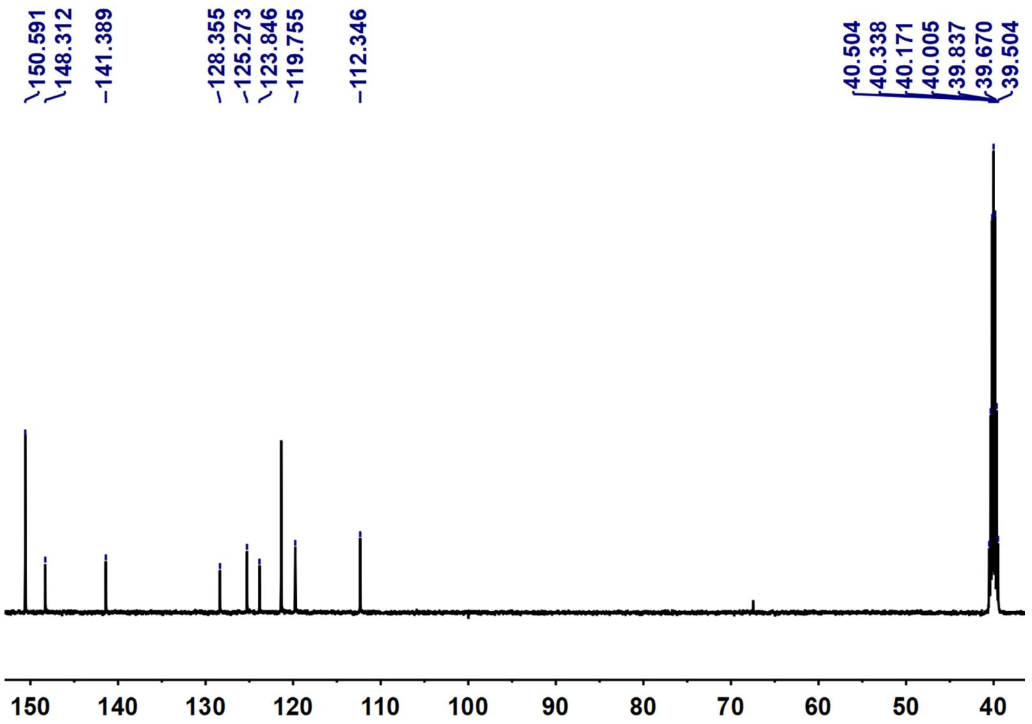

Figure S8. The ^{13}C NMR (298 K, 500 MHz, DMSO-d_6, ppm) for ligand **L1** (30.0 mM, with respect to Cp*Rh).

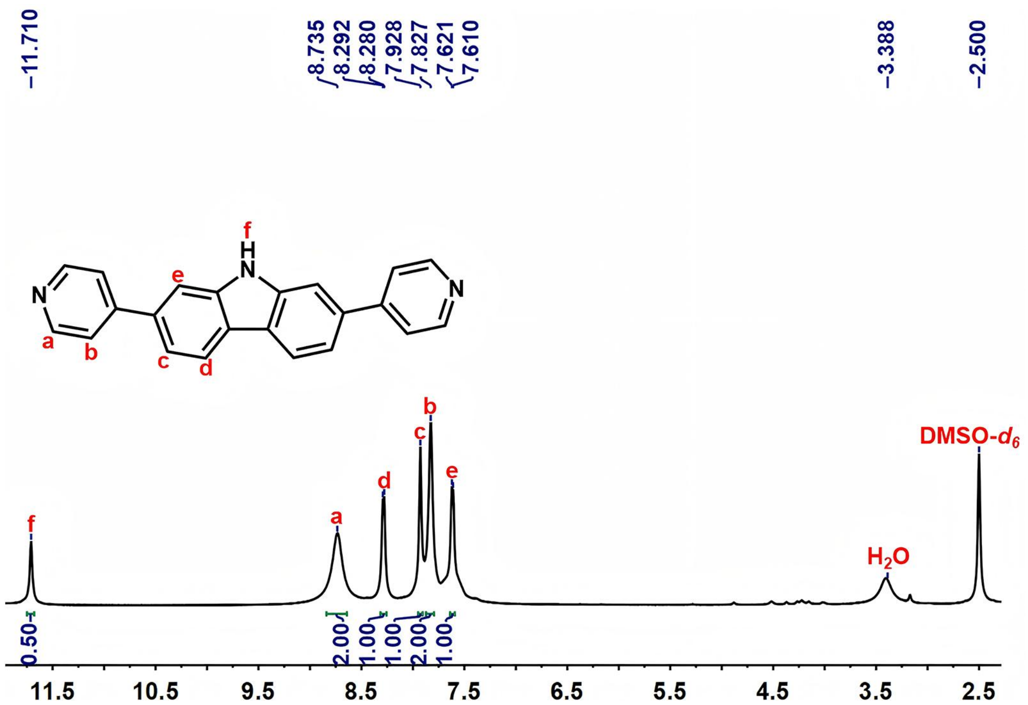

Figure S9. The ^{1}H NMR (500 MHz, DMSO-d_6, ppm) for ligand **L2** (20.0 mM, with respect to Cp*Rh).

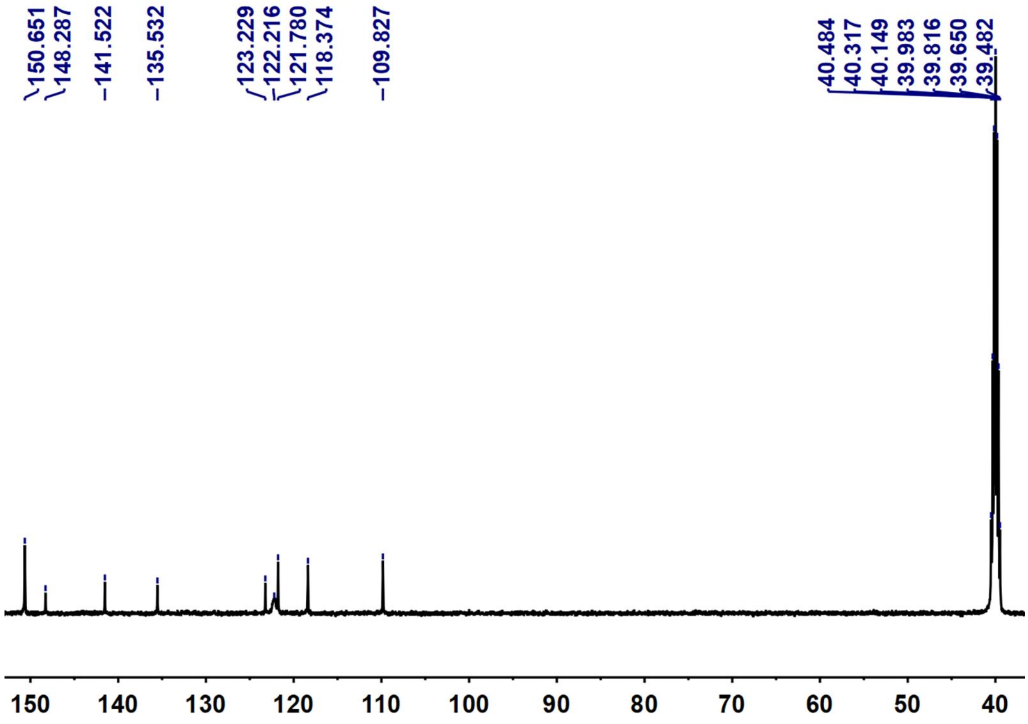

Figure S10. The ^{13}C NMR (298 K, 500 MHz, DMSO-d_6, ppm) for ligand **L2** (20.0 mM, with respect to Cp*Rh).

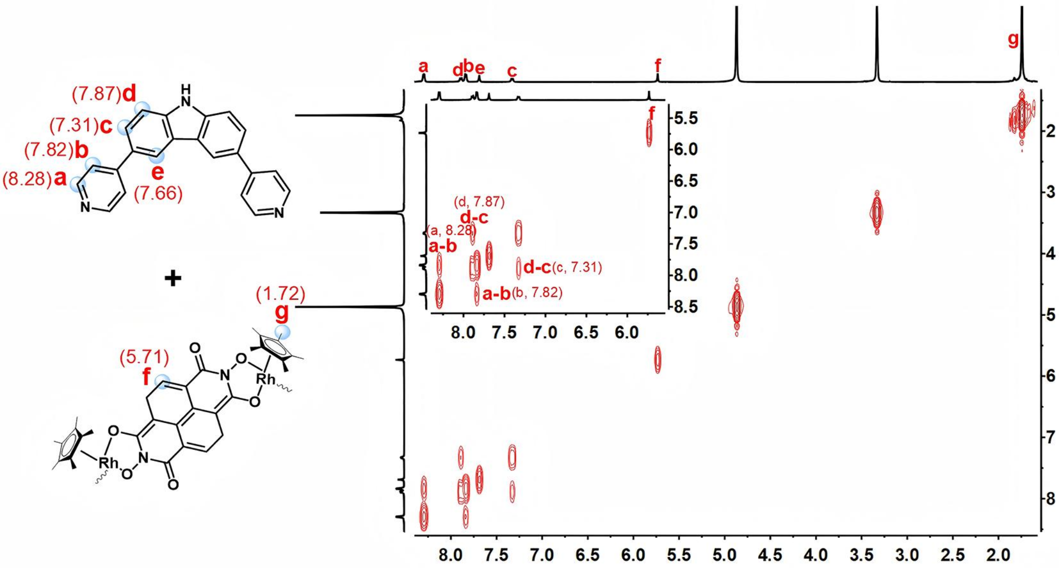

Figure S11. The ^{1}H-^{1}H COSY NMR spectrum (500 MHz, CD_3OD, 298 K) of snowflake-shaped folded assembly **2**$(OTf)_{12}$ (24.5 mM, with respect to Cp*Rh).

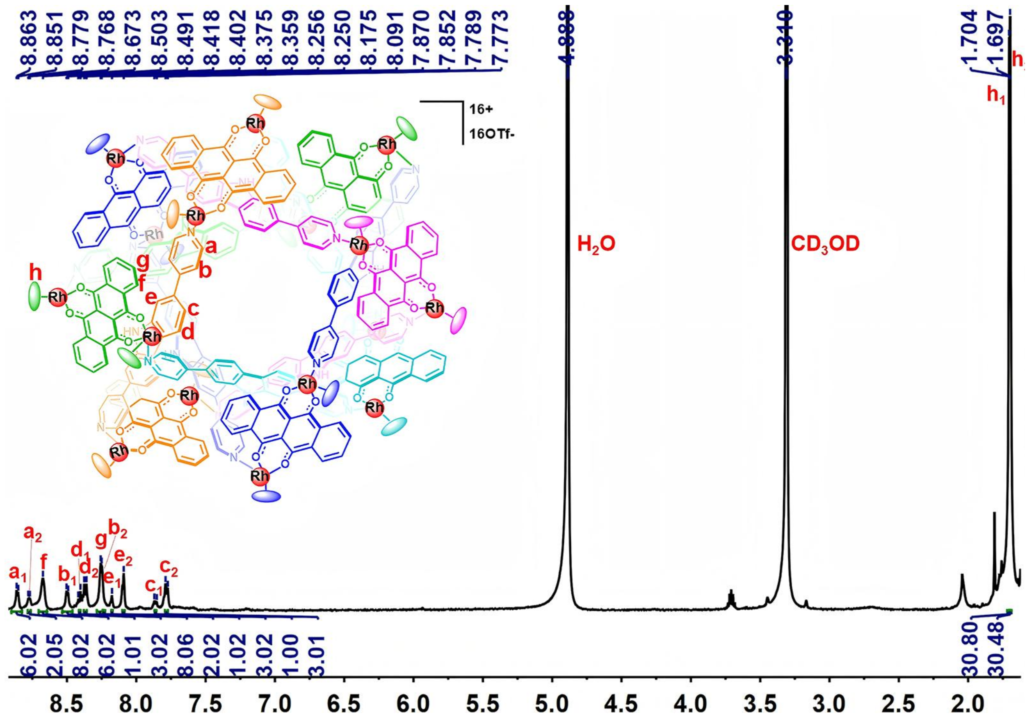

Figure S12. The ^{1}H NMR spectrum (500 MHz, CD_3OD, 298 K) of 8_{18} knot **3**$(OTf)_{16}$ (14.5 mM, with respect to Cp*Rh).

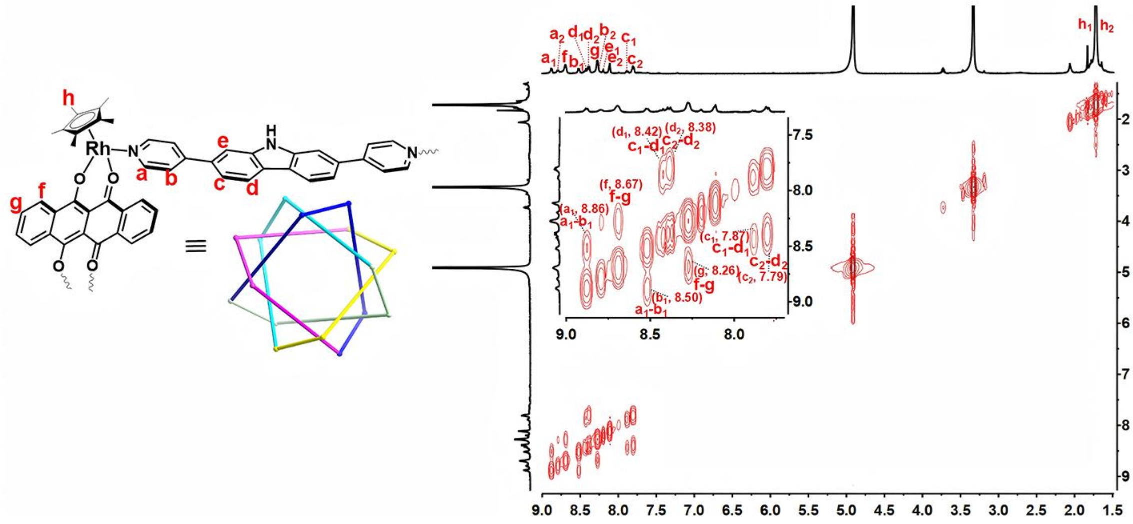

Figure S13. The ^{1}H-^{1}H COSY NMR spectrum (500 MHz, CD_3OD, 298 K) of 8_{18} knot **3**$(OTf)_{16}$ (14.5 mM, with respect to Cp*Rh).

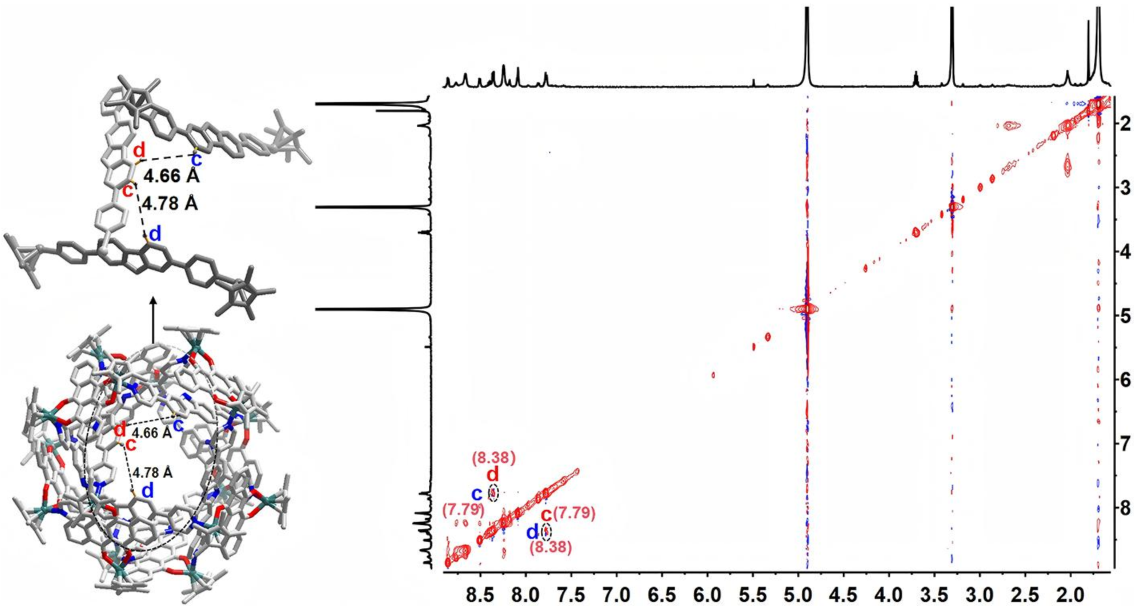

Figure S14. The ^{1}H-^{1}H NOESY NMR (500 MHz, CD_3OD, ppm) for 8_{18} knot **3**$(OTf)_{16}$ (30.0 mM, with respect to Cp*Rh).

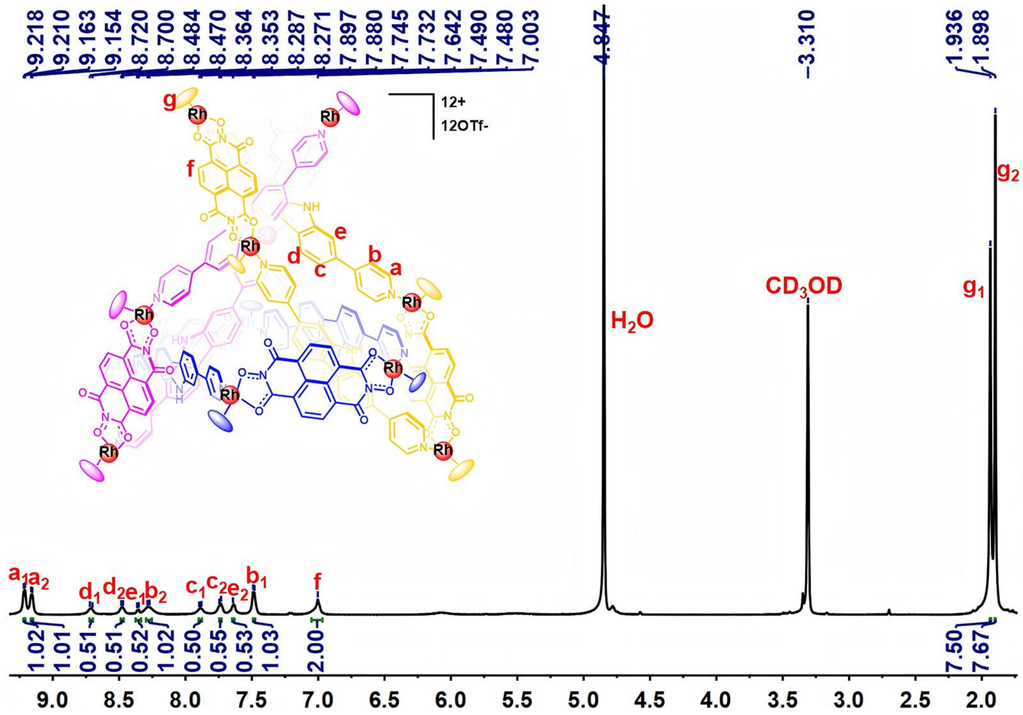

Figure S15. The ^{1}H NMR spectrum (500 MHz, CD_3OD, 298 K) of the closed three-link chain **4**$(OTf)_{12}$ (13.0 mM, with respect to Cp*Rh).

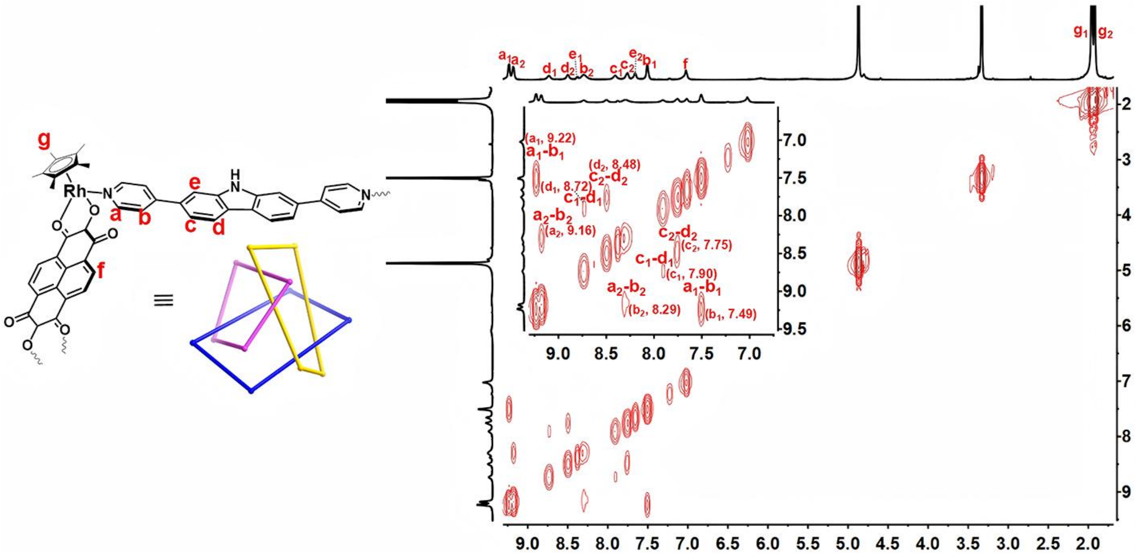

Figure S16. The ^{1}H-^{1}H COSY NMR spectrum (500 MHz, CD_3OD, 298 K) of the closed three-link chain **4**$(OTf)_{12}$ (13.0 mM, with respect to Cp*Rh).

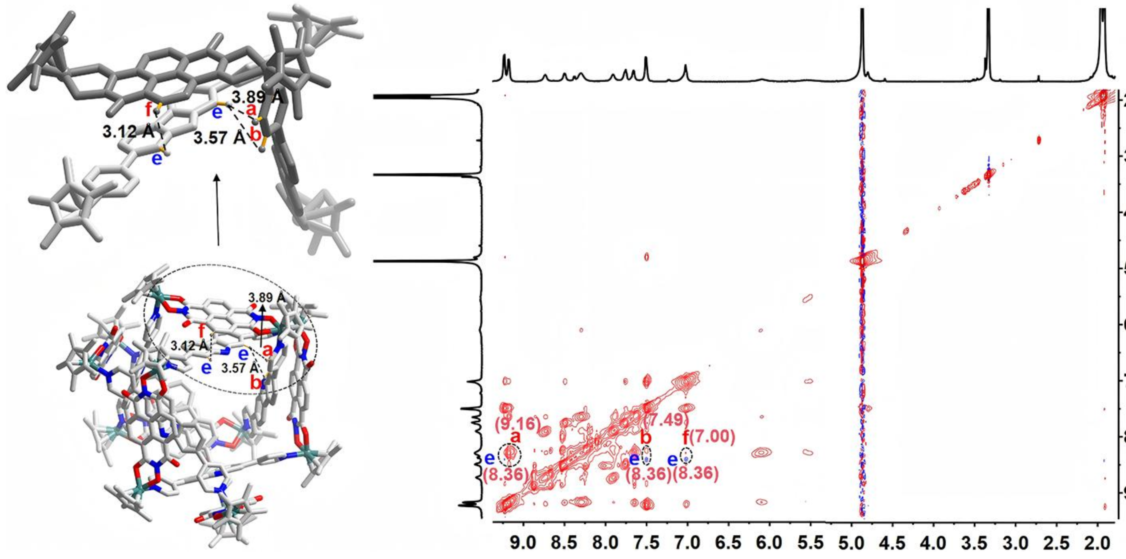

Figure S17. The ^{1}H-^{1}H NOESY NMR (500 MHz, CD_3OD, ppm) for the closed three-link chain **4**$(OTf)_{12}$ (25.0 mM, with respect to Cp*Rh).

5. Hydrodynamic radii

The size of the obtained compounds was calculated from the ^{1}H DOSY spectra. For the sphere model, according to the Stokes-Einstein equation, the hydrodynamic radii (Rh) of the obtained compounds particles have the relationship with diffusion coefficients (D):

$$D = \frac{K_B T}{6\pi\eta R_h} \quad (1)$$

"kB" is the Boltzmann constant ($1.3806488 \times 10^{-23}$ J/K), "T" is the experimental temperature (293 K), and "η" is the viscosity of CD_3OD (0.00058 Pa s). The hydrodynamic radii of **2**$(OTf)_{12}$ is 14.82 Å ($D = 2.54 \times 10^{-6}$ cm^2 s^{-1}). The hydrodynamic radii of **3**$(OTf)_{16}$ is 14.79 Å ($D = 2.55 \times 10^{-6}$ cm^2 s^{-1}). The hydrodynamic radii of **4**$(OTf)_{12}$ is 14.36 Å ($D = 2.62 \times 10^{-6}$ cm^2 s^{-1}).

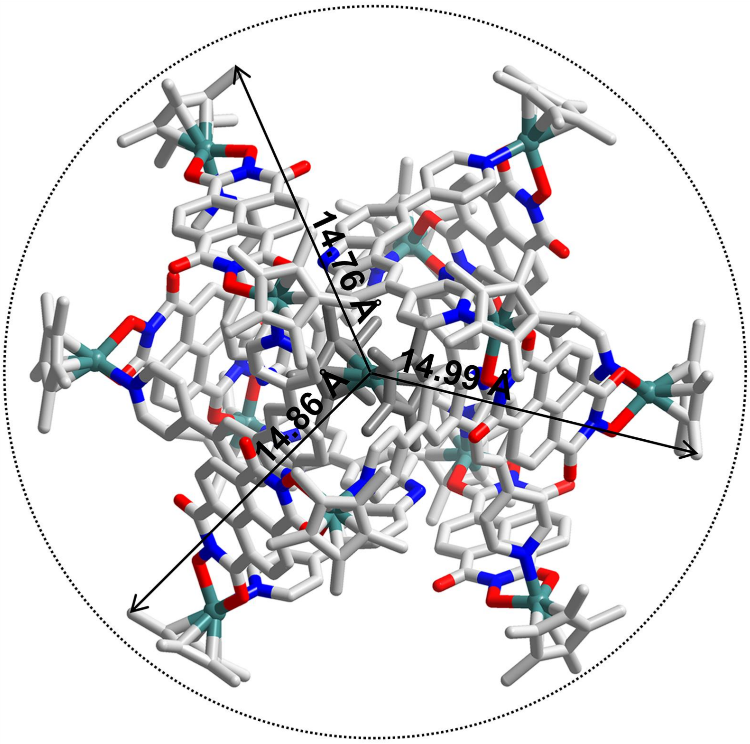

Figure S18. Space-filling model of **2**$(OTf)_{12}$, fitted according to a spherical model ($D = 2.54 \times 10^{-6}$ cm^2 s^{-1}, r_h = 14.82 Å).

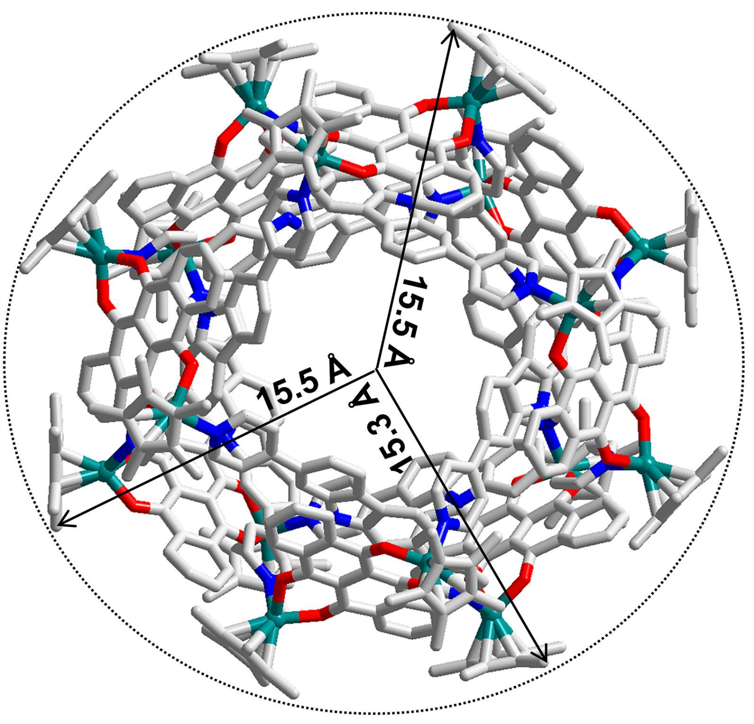

Figure S19. Space-filling model of **3**$(OTf)_{16}$, fitted according to a spherical model ($D = 2.55 \times 10^{-6}$ $cm^2\ s^{-1}$, $r_h = 14.79$ Å).

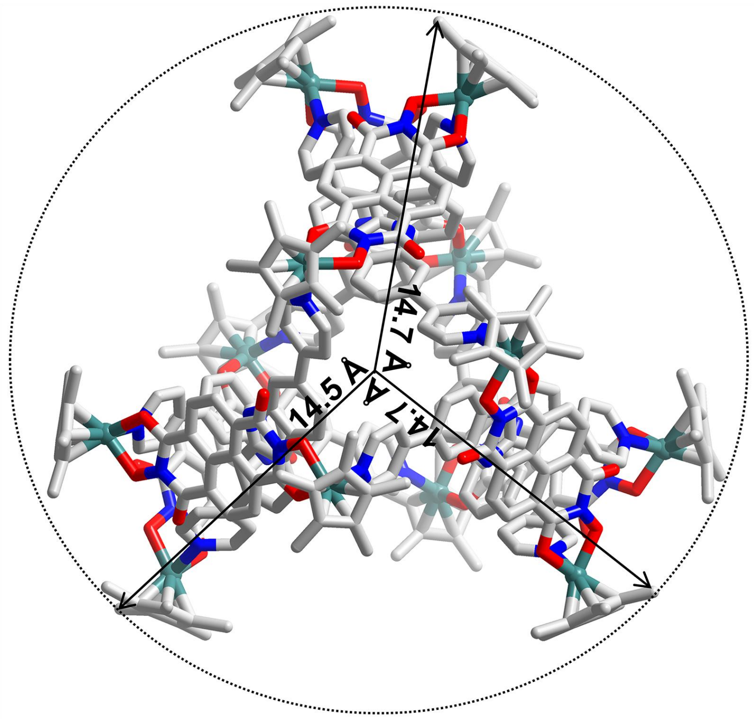

Figure S20. Space-filling model of **4**$(OTf)_{12}$, fitted according to a spherical model ($D = 2.62 \times 10^{-6}$ $cm^2\ s^{-1}$, $r_h = 14.36$ Å).

6. ESI-TOF-MS spectra

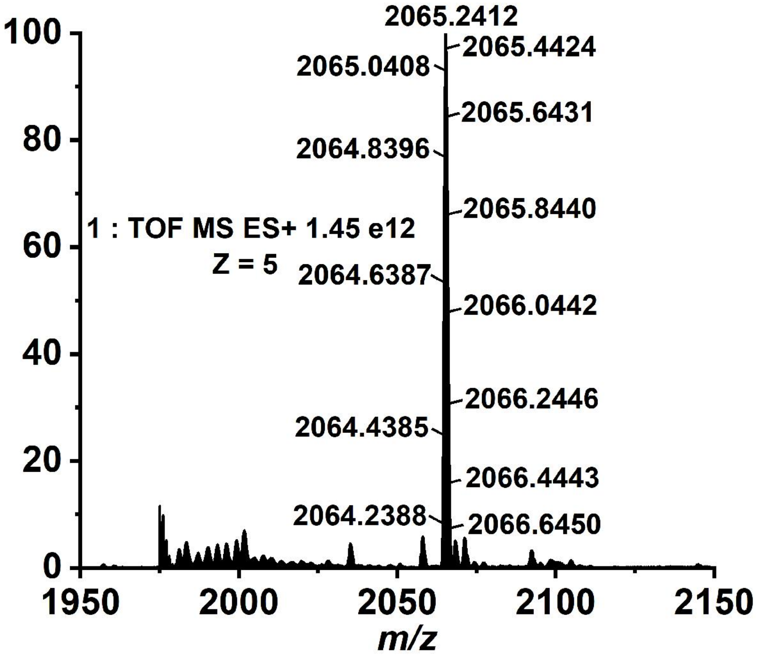

Figure S21. Partial ESI-TOF-MS spectrum of 8_{18} knot **3**$(OTf)_{16}$ ($[\mathbf{3}(OTf)_{16}\text{-}5OTf^{-}]^{5+}$).

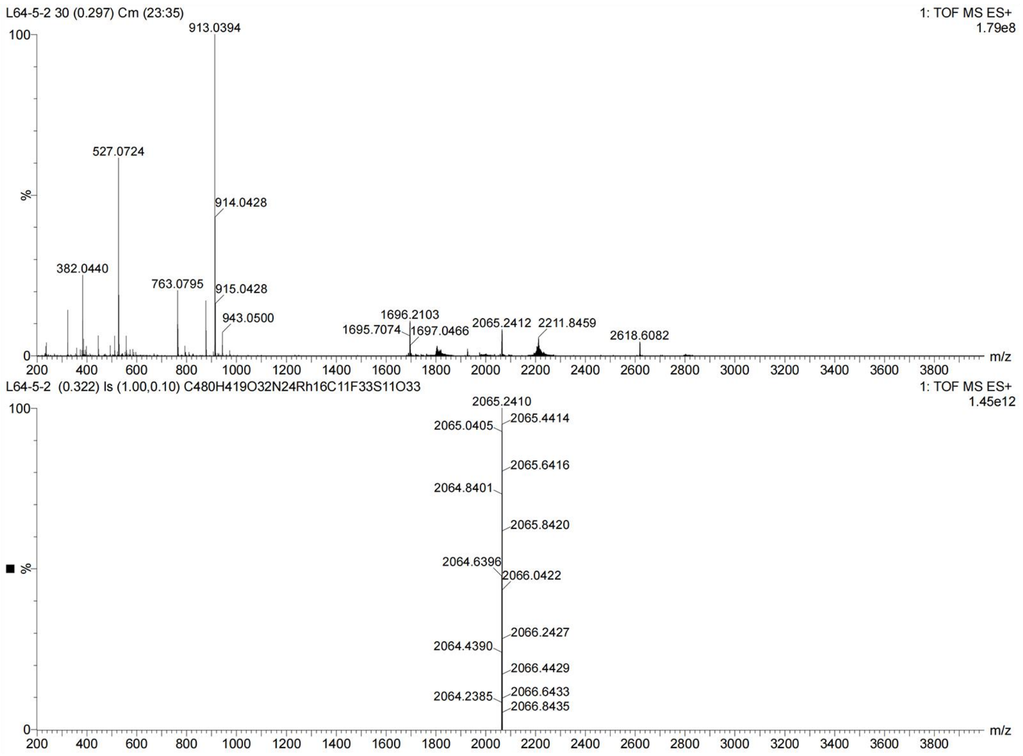

Figure S22. Full ESI-TOF-MS spectra of 8_{18} knot **3**$(OTf)_{16}$ ($[\mathbf{3}(OTf)_{16}\text{-}5OTf^{-}]^{5+}$).

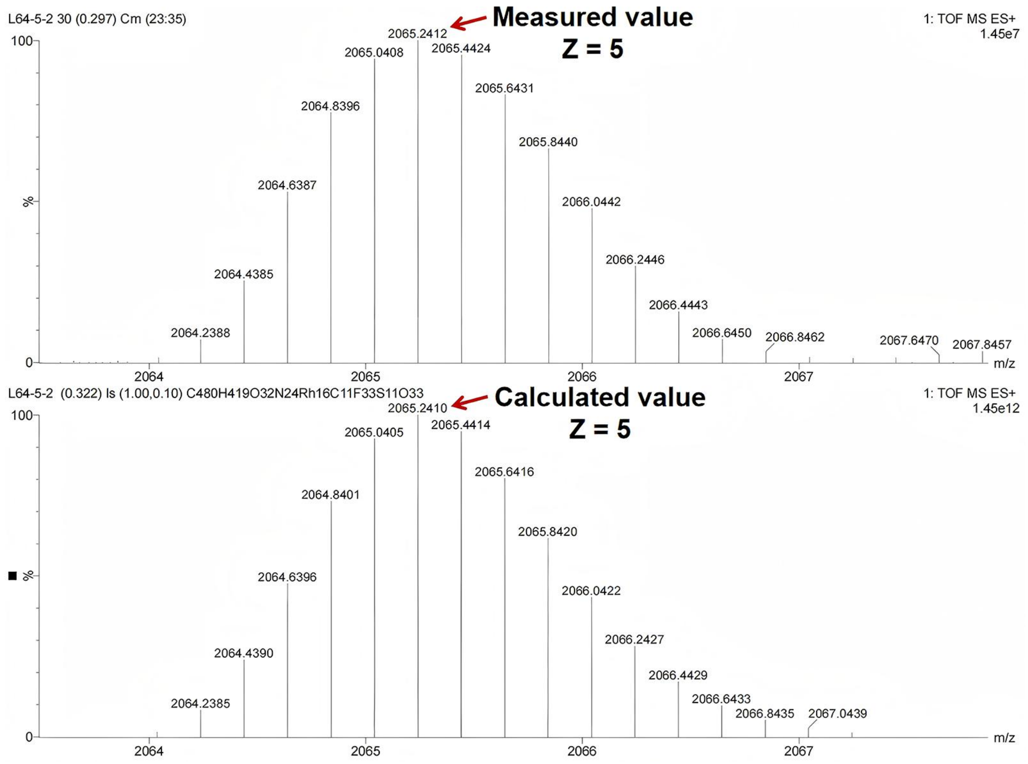

Figure S23. The ESI-TOF-MS spectra of 8_{18} knot **3**(OTf)$_{16}$ ([**3**(OTf)$_{16}$-5OTf$^-$]$^{5+}$). (Measured value, upper; Calculated value, lower).

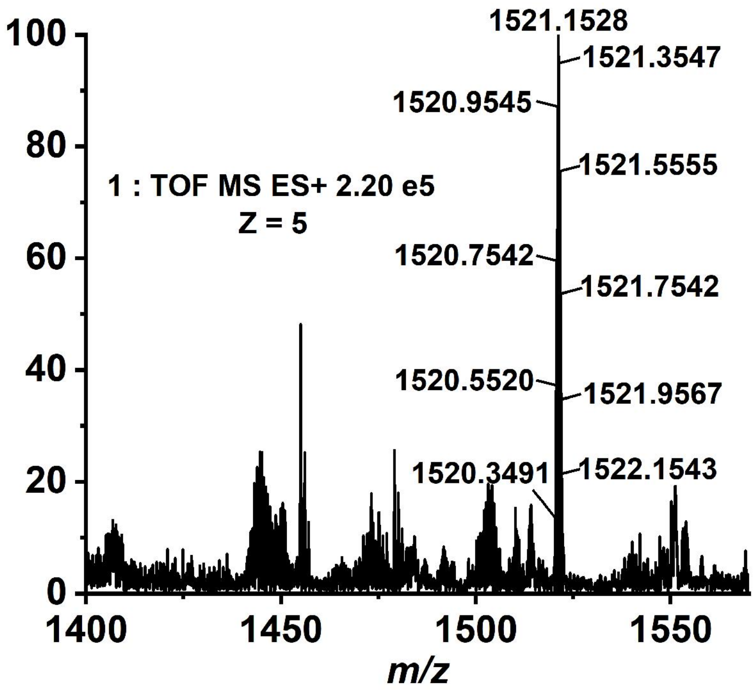

Figure S24. Partial ESI-TOF-MS spectrum of the closed three-link chain **4**(OTf)$_{12}$ ([**4**(OTf)$_{12}$-5OTf$^-$]$^{5+}$).

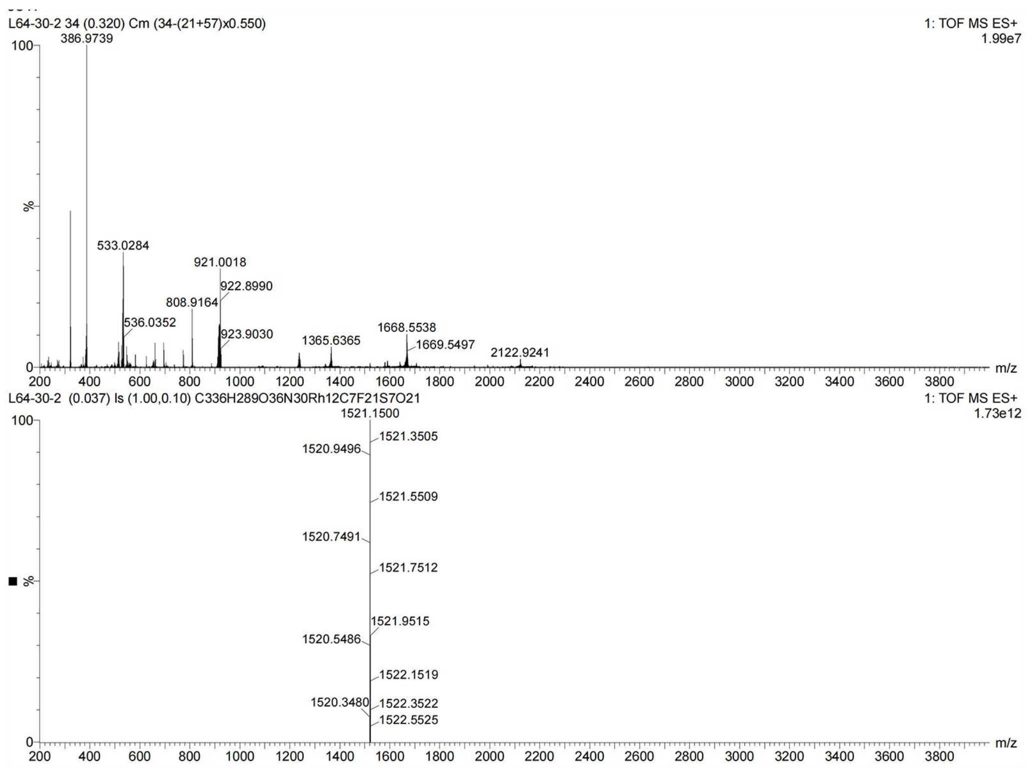

Figure S25. Full ESI-TOF-MS spectra of closed three-link chain **4**$(OTf)_{12}$ ([**4**$(OTf)_{12}$-5OTf$^-$]$^{5+}$).

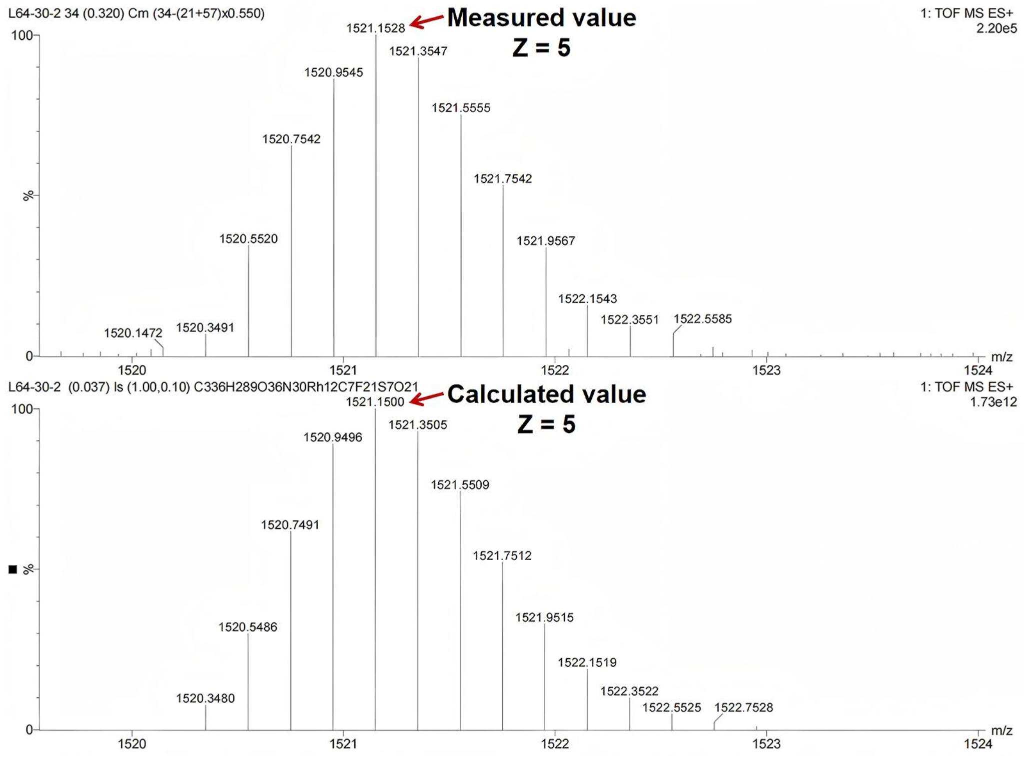

Figure S26. The ESI-TOF-MS spectra of the closed three-link chain **4**$(OTf)_{12}$ ([**4**$(OTf)_{12}$-5OTf$^-$]$^{5+}$).

7. Experimental details of photothermal conversion

Details in solution:

Since compounds **3**$(OTf)_{16}$ and **4**$(OTf)_{12}$ contain eight and six sets of intramolecular *π–π* stacking interactions, respectively, a molar ratio of 3:4 for compounds **3**$(OTf)_{16}$ and **4**$(OTf)_{12}$ was used to ensure the same conjugated *π* area. **3**$(OTf)_{16}$ (2.77 mg, 0.00025 mmol) was added to methanol (3 mL), and **4**$(OTf)_{12}$ (2.78 mg, 0.00033 mmol) was added to another portion of methanol (3 mL). After the solid dissolved absolutely, 2.0 mL of this solution was taken into a quartz spectrophotometer cell (1 × 1 × 5 cm) to measure its absorbance and put into the bright spot of a laser with a 730 nm wavelength at different current intensities. Temperature variation of the solution was detected by an infrared camera. The methanol solution in each contained **3**$(OTf)_{16}$ and **4**$(OTf)_{12}$ and was put in a 0.5 mL sample vessel. And then, they were put under a laser and temperature variations were detected by the same infrared camera. Equations used to calculate photothermal conversion efficiency were exhibited as follows:

$$\eta = hS(\Delta T_{sample} - \Delta T_{solvent}) / I(1 - 10^{-A}) \quad (2)$$

$$hS = \sum mC_p/\tau_s \quad (3)$$

$$\tau_s = -t/\ln\theta \quad (4)$$

$$\theta = (T_{amb} - T)/(T_{amb} - T_{max}) \quad (5)$$

Thereinto, $\mathbf{C_p}$ = 2.51 KJ/(Kg K), $\Delta T_{solvent}$ = 2.2°C. **I** = 0.6 W/cm^2, **h** is the heat transfer coefficient, and **S** is the surface area of the container. $\boldsymbol{\tau_s}$ is the sample system time constant, m is the mass of the products. Thus, $\sum mC_p = m_{(methanol)} C_{p(methanol)} = \rho_{(methanol)} V_{(methanol)} C_{p(methanol)} = 0.791 \times 0.7 \times 2.51 = 1.3898$ J K^{-1}. $\Delta T_{solvent}$ = 2.2°C, where **I** is the laser power and A_{730} is the absorbance of the samples at the wavelength of 730 nm.

Strictly speaking, the volume of the mixed solvent was not a simple addition of the two components. However, the deviation was small enough to ignore it.

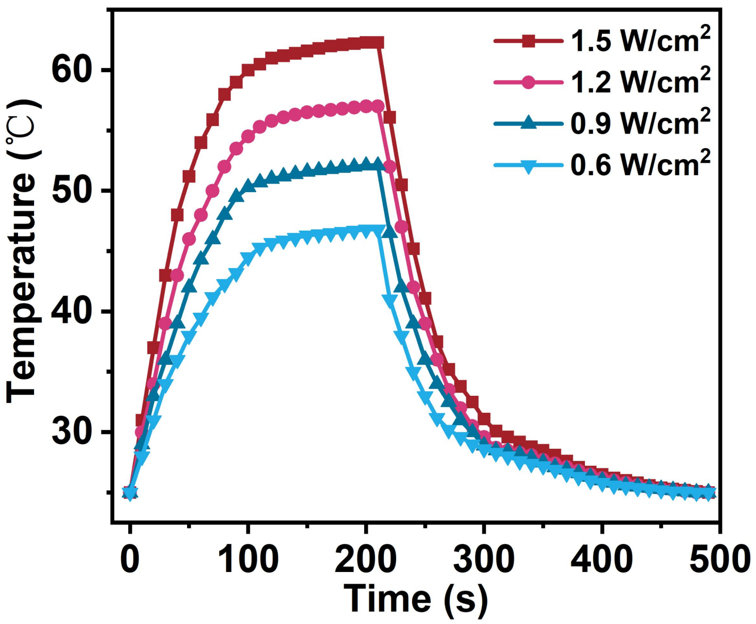

Figure S27. Photothermal conversion curves of **3**(OTf)$_{16}$ in methanol.

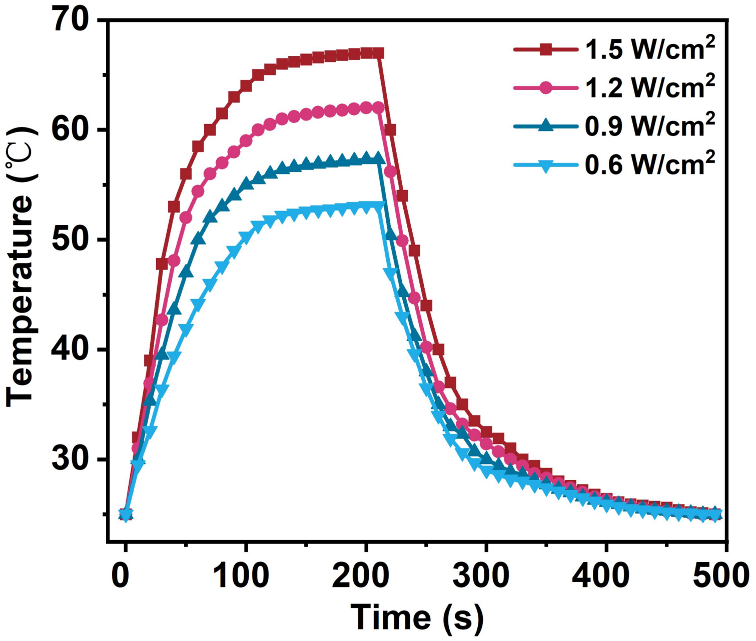

Figure S28. Photothermal conversion curves of **4**(OTf)$_{12}$ in methanol.

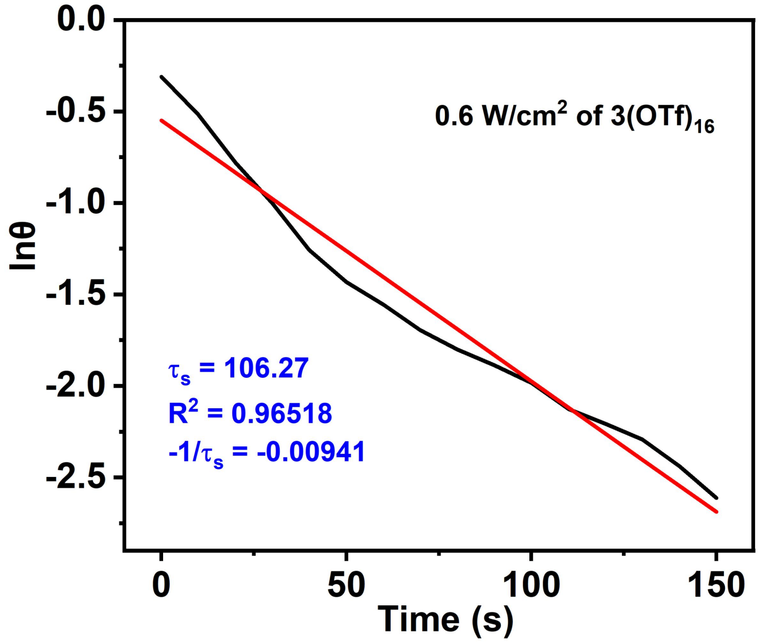

Figure S29. Fitting a linear of lnθ-t of 0.6 W/cm^2 of **3**(OTf)$_{16}$ in methanol.

Photothermal conversion efficiency η of **3**(OTf)$_{16}$ at 0.6 W/cm^2 was calculated by the equations above. A fitting linear of lnθ-t was obtained by Eqs (4) and (5), by which τ_s was calculated as 106.27 s. Thus, hS = 1.3898 / 106.27 = 1.31 × 10^{-2} J K^{-1} S^{-1}. ΔT_{sample} = 21.8°C (46.8°C - 25°C). A = 0.603. η = 1.31 × 10^{-2} × (21.8 - 2.2) / [0.603 × (1 - 10$^{-0.603}$)] = 56.92%.

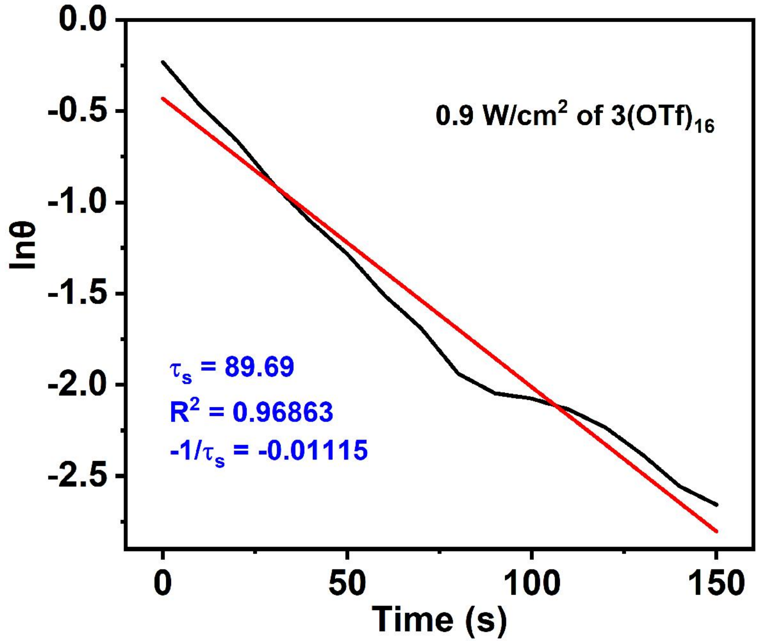

Figure S30. Fitting a linear of lnθ-t of 0.9 W/cm^2 of **3**(OTf)$_{16}$ in methanol.

The photothermal conversion efficiency η of **3**(OTf)$_{16}$ at 0.9 W/cm^2 was calculated by the equations above. A fitting linear of lnθ-t was obtained by Eqs (4) and (5), by which τs was calculated as 89.69

s. Thus, hS = 1.3898 / 89.69 = 1.55 × 10^{-2} J K^{-1} S^{-1}. ΔT_{sample} = 27.1°C (52.1°C - 25°C). A = 0.603. η = 1.55 × 10^{-2} × (27.1 - 2.2) / [0.603 × (1 - $10^{-0.603}$)] = 57.12%.

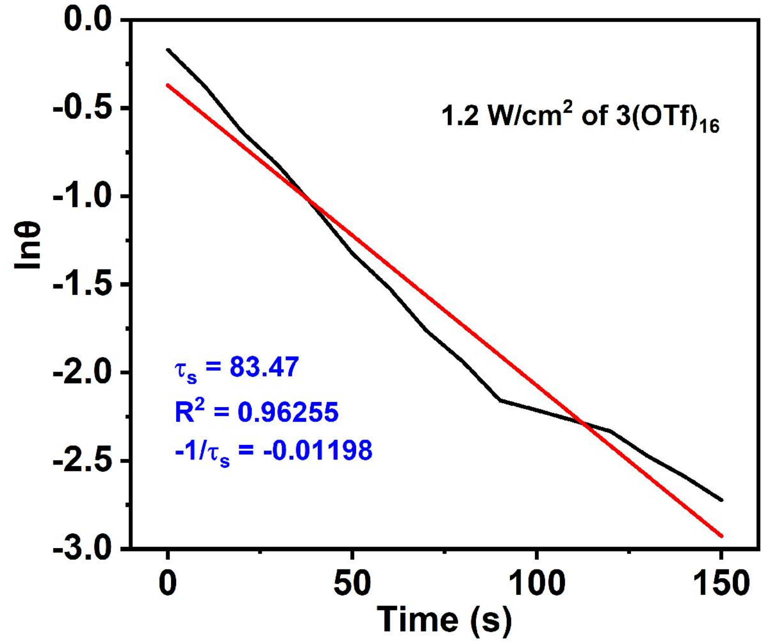

Figure S31. Fitting a linear of lnθ-t of 1.2 W/cm^2 of **3**(OTf)$_{16}$ in methanol.

The photothermal conversion efficiency η of **3**(OTf)$_{16}$ at 1.2 W/cm^2 was calculated by the equations above. A fitting linear of lnθ-t was obtained by Eqs (4) and (5), by which τ_s was calculated as 83.47 s. Thus, hS = 1.3898 / 83.47 = 1.67 × 10^{-2} J K^{-1} S^{-1}. ΔT_{sample} = 32°C (57°C - 25°C). A = 0.603. η = 1.67 × 10^{-2} × (32 - 2.2) / [0.603 × (1 - $10^{-0.603}$)] = 55.09%.

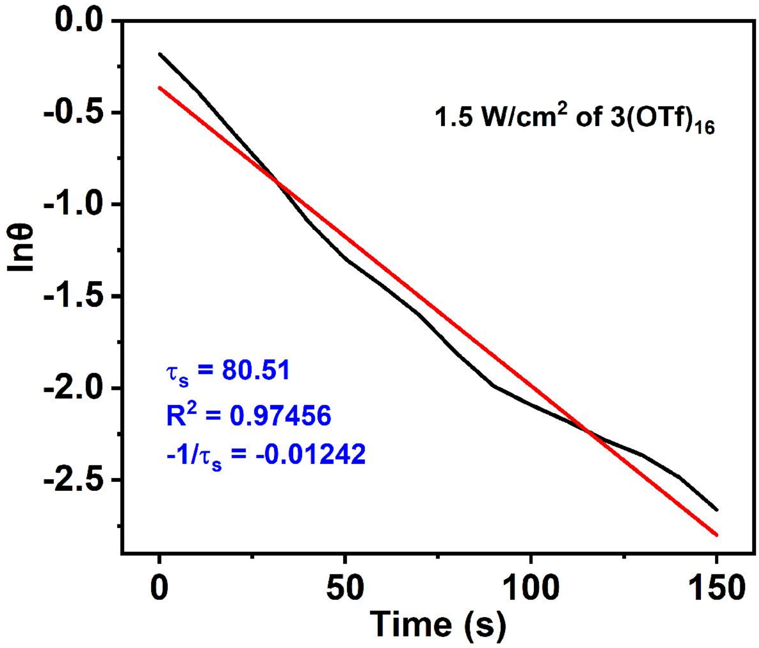

Figure S32. Fitting a linear of lnθ-t of 1.5 W/cm^2 of **3**(OTf)$_{16}$ in methanol.

The photothermal conversion efficiency η of **3**$(OTf)_{16}$ at 1.5 W/cm^2 was calculated by the equations above. A fitting linear of lnθ-t was obtained by Eqs (4) and (5), by which τ_s was calculated as 80.51 s. Thus, hS = 1.3898/80.51 = 1.73 × 10^{-2} J K^{-1} S^{-1}. ΔT_{sample} = 37.3°C (62.3°C - 25°C). A = 0.603. η = 1.73 × 10^{-2} × (37.3 - 2.2) / [0.603 × (1 - 10$^{-0.603}$)] = 53.82%.

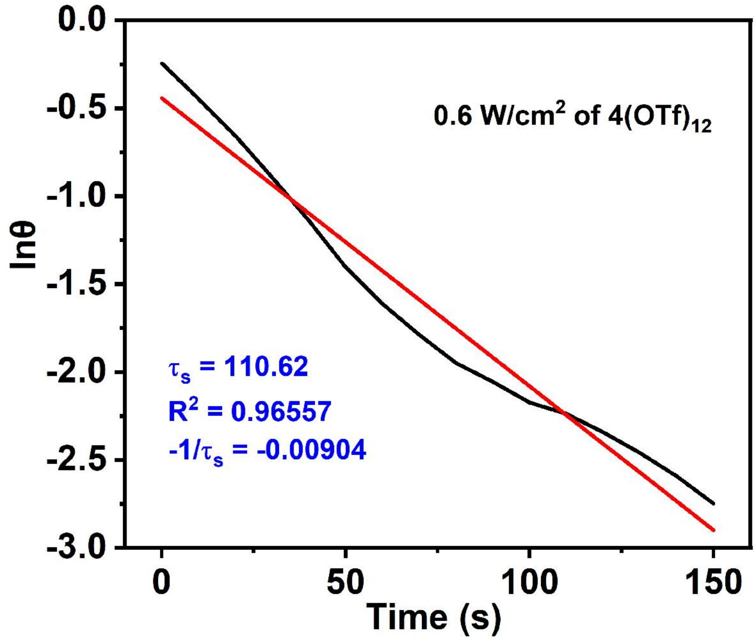

Figure S33. Fitting a linear of lnθ-t of 0.6 W/cm^2 of **4**$(OTf)_{12}$ in methanol.

Photothermal conversion efficiency η of **4**$(OTf)_{12}$ at 0.6 W/cm^2 was calculated by the equations above. A fitting linear of lnθ-t was obtained by Eqs (4) and (5), by which τ_s was calculated as 110.62 s. Thus, hS = 1.3898 / 110.62 = 1.26 × 10^{-2} J K^{-1} S^{-1}. ΔT_{sample} = 28.1°C (53.1°C - 25°C). A = 0.674. η = 1.26 × 10^{-2} × (28.1 - 2.2) / [0.674 × (1 - 10$^{-0.674}$)] = 68.81%.

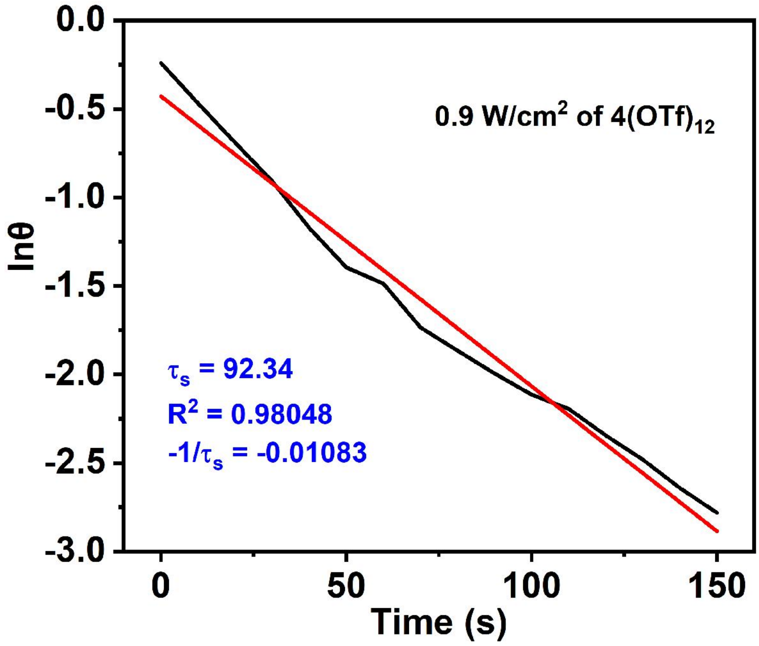

Figure S34. Fitting a linear of lnθ-t of 0.9 W/cm^2 of **4**$(OTf)_{12}$ in methanol.

The photothermal conversion efficiency η of **4**$(OTf)_{12}$ at 0.9 W/cm^2 was calculated by the equations above. A fitting linear of lnθ-t was obtained by Eqs (4) and (5), by which τ_s was calculated as 92.34 s. Thus, hS = 1.3898 / 92.34 = 1.51 × 10^{-2} J K^{-1} S^{-1}. ΔT_{sample} = 32.3°C (57.3°C - 25°C). A = 0.674. η = 1.51 × 10^{-2} × (32.3 - 2.2) / [0.674 × (1 - $10^{-0.674}$)] = 63.86%.

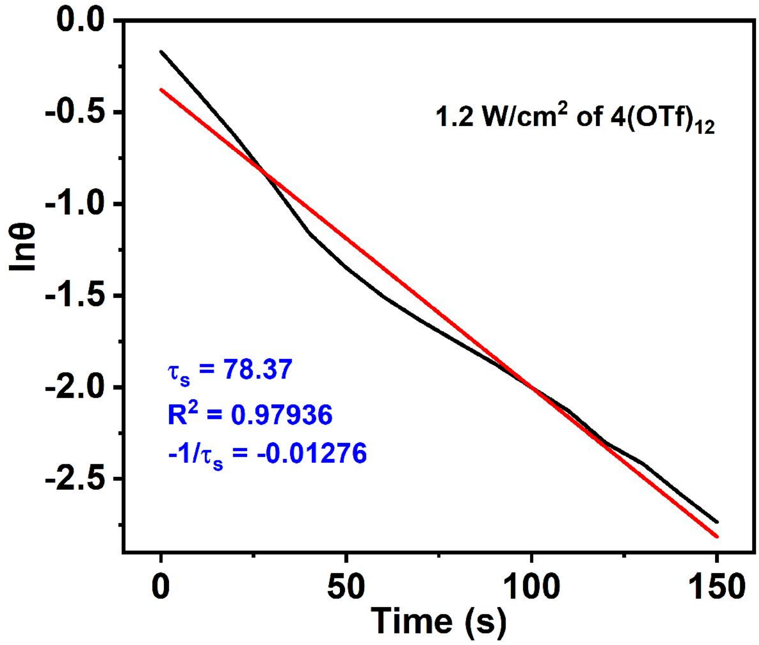

Figure S35. Fitting a linear of lnθ-t of 1.2 W/cm^2 of **4**$(OTf)_{12}$ in methanol.

The photothermal conversion efficiency η of **4**$(OTf)_{12}$ at 1.2 W/cm^2 was calculated by the equations above. A fitting linear of lnθ-t was obtained by Eqs (4) and (5), by which τ_s was calculated as 78.37

s. Thus, hS = 1.3898 / 78.37 = 1.77 × 10^{-2} J K^{-1} S^{-1}. ΔT_{sample} = 37°C (62°C - 25°C). A = 0.674. η = 1.77 × 10^{-2} × (37 - 2.2) / [0.674 × (1 - $10^{-0.674}$)] = 65.25%.

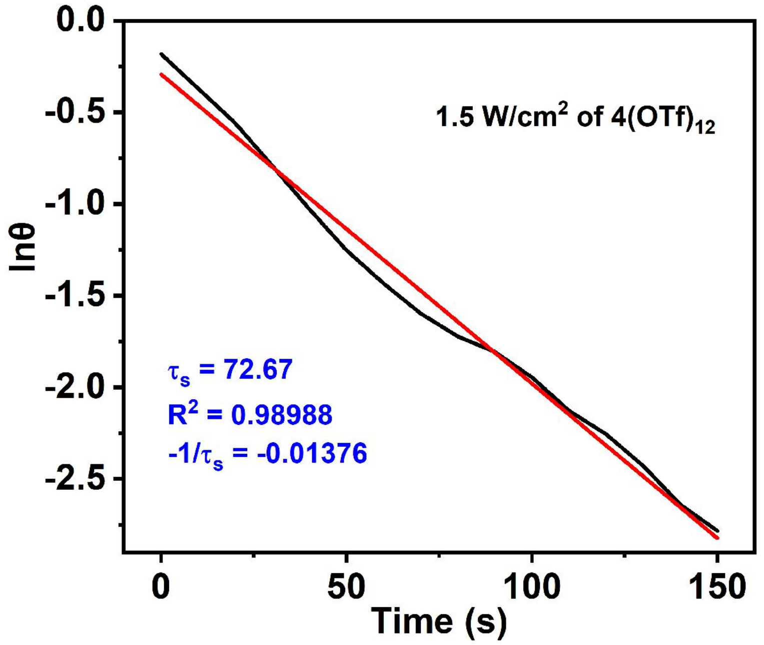

Figure S36. Fitting a linear of lnθ-t of 1.5 W/cm^2 of **4**$(OTf)_{12}$ in methanol.

The photothermal conversion efficiency η of **4**$(OTf)_{12}$ at 1.5 W/cm^2 was calculated by the equations above. A fitting linear of lnθ-t was obtained by Eqs (4) and (5), by which τ_s was calculated as 72.67 s. Thus, hS = 1.3898 / 72.67 = 1.91 × 10^{-2} J K^{-1} S^{-1}. ΔT_{sample} = 42°C (67°C - 25°C). A = 0.674. η = 1.91 × 10^{-2} × (42 - 2.2) / [0.674 × (1 - $10^{-0.674}$)] = 64.38%.

**Table S1.** Comparison of materials in photothermal conversion with other literature systems.				
Materials	Wavelength	Temperature rise range	Photothermal conversion efficiency	References
ABI NYs	808 nm	5°C - 50.2°C	35.6%	*Materials Today* Bio 2024; **26**: 101052.
rPMo·cTMB	1064 nm	25°C - 60°C	48.4%	*Adv Healthcare* Mater 2022, **11**: 2102352.
B-POM NPs	1064 nm	25°C - 55°C	57.2%	*Chem Eng J,* 2024, **490**: 151601.
ir-Na-CDs@BSA	1064 nm	25°C - 67.8°C	43%	*Adv Funct Mater* 2024; **34**: 2402976.
Cluster-400	808 nm	25°C - 53.3°C	51.1%	*Chem Eng J* 2025; **504**: 159045.
PFQ	808 nm	20°C - 43°C	44.74%	*ACS Appl Mater Interfaces* 2022; **14**: 28781–28791.
matchstick-like Bi_2Se_3/CdSe-Au	808 nm	25.0°C - 62.9°C	60.3%	*Adv Funct Mater* 2021; **31**: 2104424.
BAF4	1064 nm	25.0°C - 60°C	80%	*Angew Chem Int Ed* 2021; **60**: 22376–22384.
**3**$(OTf)_{16}$	730 nm	25.0°C - 62.3°C	57.12%	**In work**
**4**$(OTf)_{12}$	730 nm	25.0°C - 67°C	68.81%	**In work**

Table S2 Comparison of supramolecular complexes in photothermal conversion with other literature systems.

Supramolecular complexes	Wavelength	Temperature rise range	Photothermal conversion efficiency	References
Tweezer-like molecular assembly 2	660 nm	25°C - 35°C	11.83%	*Chin J Chem* 2023; **41**: 3229–3237.
Tetranuclear Macrocycles 1	730 nm	21.6°C - 33.6°C	15.6%	*J Am Chem Soc* 2023; **145**: 18036–18047.
[2]catenane 3a	730 nm	25.8°C - 50.3°C	40.42%	*Chin Chem Lett* 2025; **36**: 111460.
cyclic [4]catenane 1	730 nm	24.8°C-44.5°C	24.3%	*Angew Chem Int Ed* 2025; **64**: 202422444.
[2]catenane Rh-2	660 nm	27.6°C - 45.8°C	58.29%	*Sci China Chem* 2025; **68**: 1345–1354.
[3]catenane 2	730 nm	21.3°C - 37.9°C	62.4%	*J Am Chem Soc* 2021; **143**: 17833−17842.
$MCAT_{CAAC}$	808 nm	23.4°C - 72.7°C	79.6%	*Nat Commun* 2026; **17**: 1632.
Compound 1	1064 nm	15.0°C - 74.3°C	84.4%	*Chem Eng J* 2024; **499**: 156059.
**3**$(OTf)_{16}$	730 nm	25.0°C - 62.3°C	53.82%	**In work**
**4**$(OTf)_{12}$	730 nm	25.0°C - 67°C	64.38%	**In work**

8. Thermogravimetric analysis

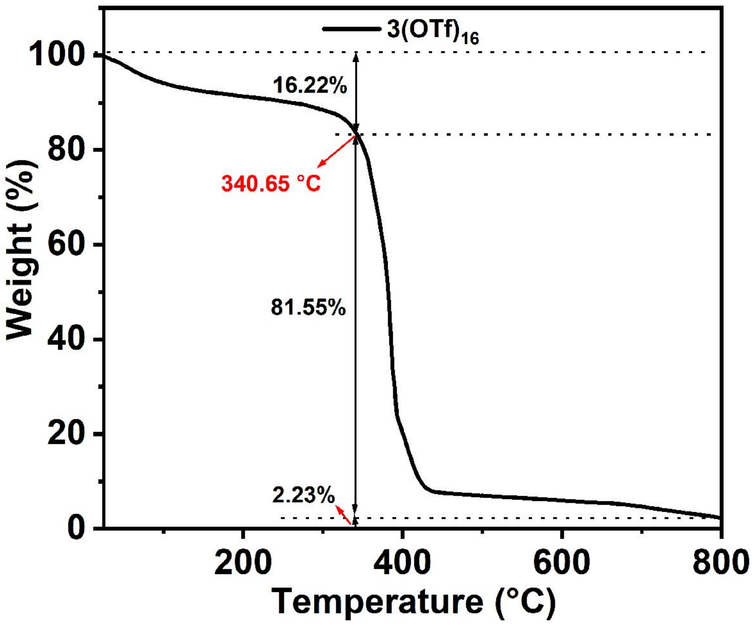

Figure S37. Thermogravimetric analysis experiment of **3**$(OTf)_{16}$.

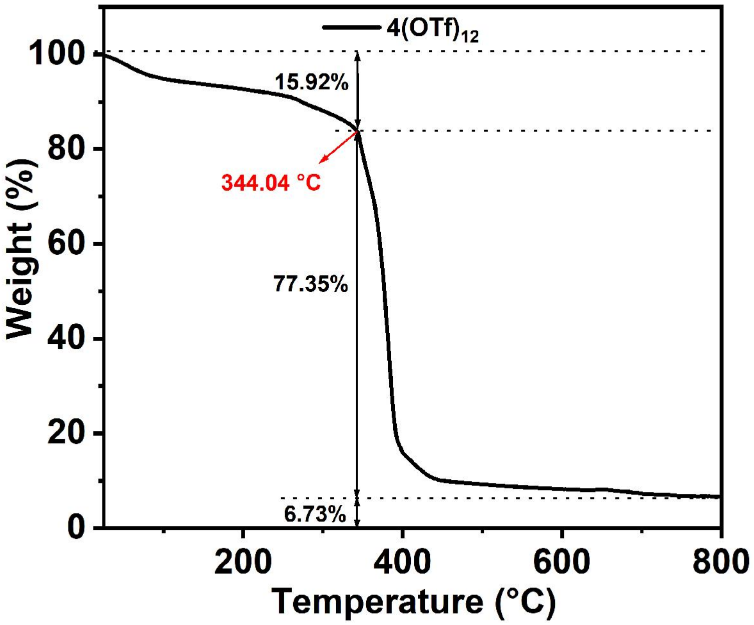

Figure S38. Thermogravimetric analysis experiment of **4**$(OTf)_{12}$.

9. Theoretical arithmetic

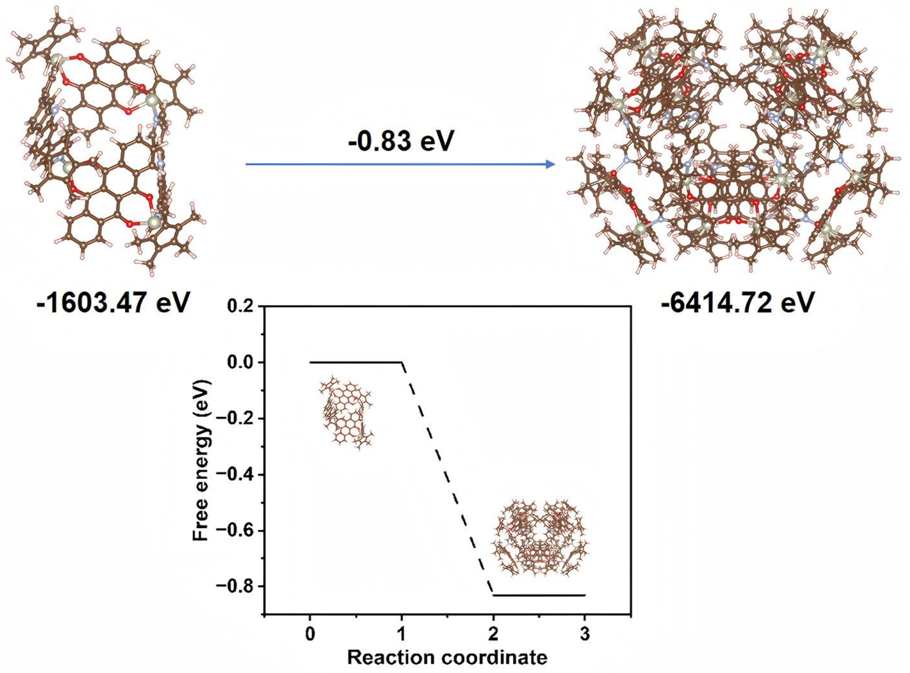

Figure S39. Structural evolution and free energy profile of the 8_{18} knot self-assembly process.

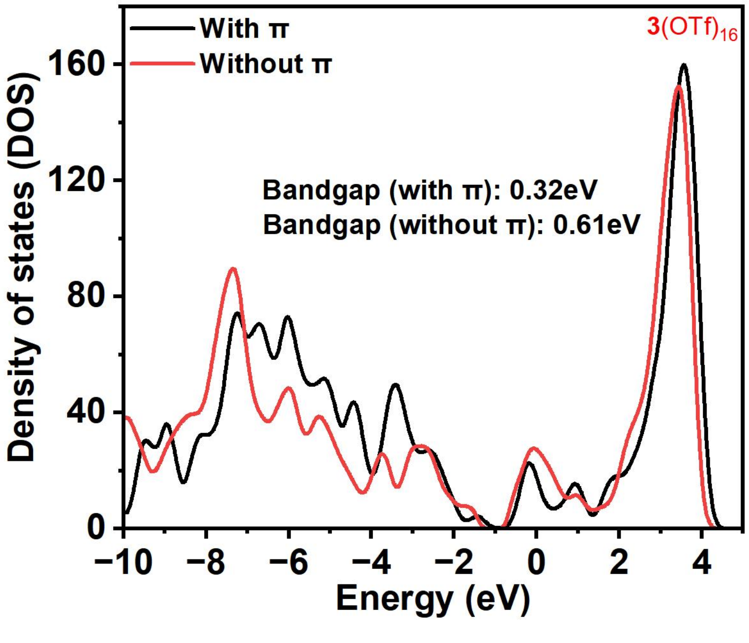

Figure S40. Density of states of **3**$(OTf)_{16}$ with (gray) and without (red) π-π stacking.

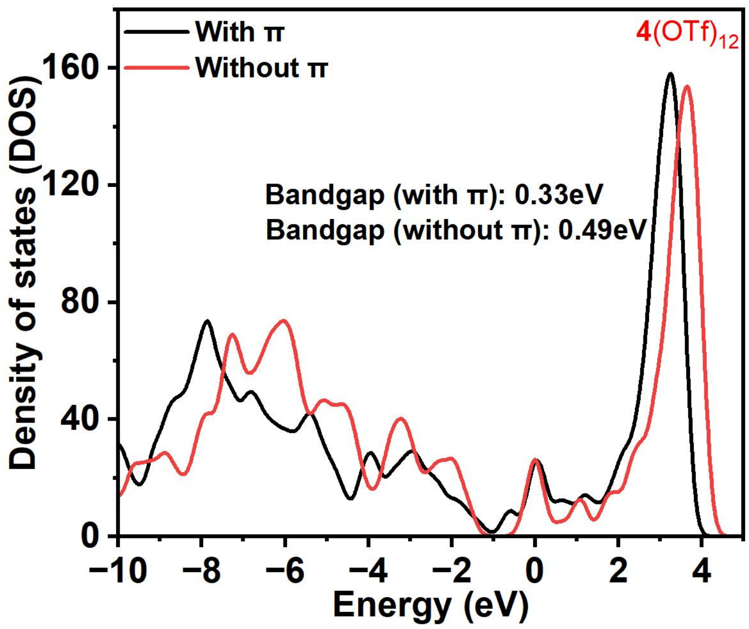

Figure S41. Density of states of **4**(OTf)$_{12}$ with (gray) and without (red) π-π stacking.

10. Measurement of photophysical properties

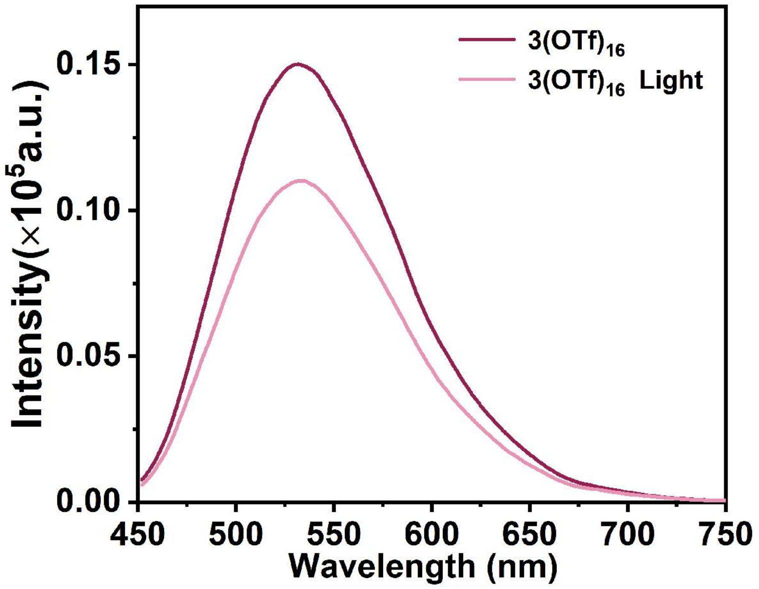

Figure S42. Fluorescence spectra comparison of **3**(OTf)$_{16}$ before and after light irradiation.

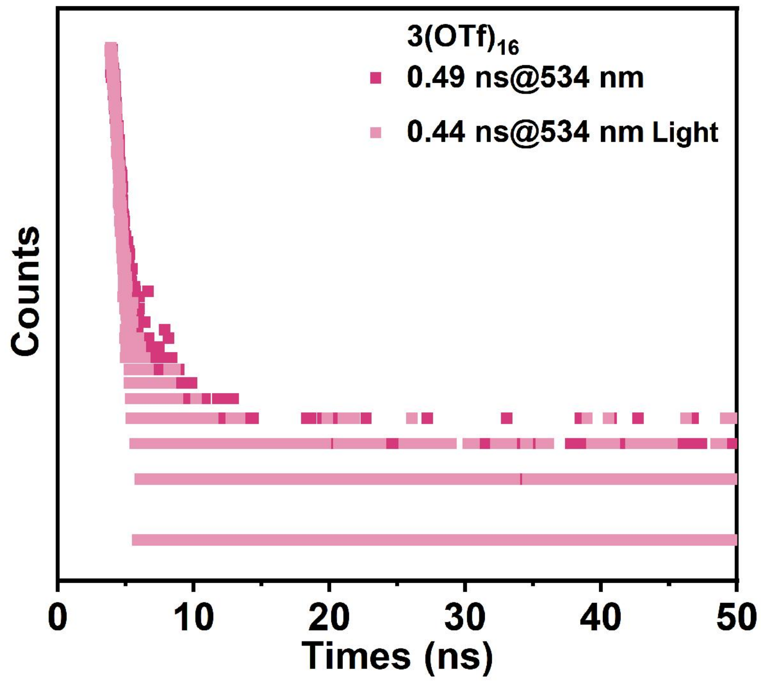

Figure S43. Fluorescence decay curve of **3**$(OTf)_{16}$ before and after light irradiation.

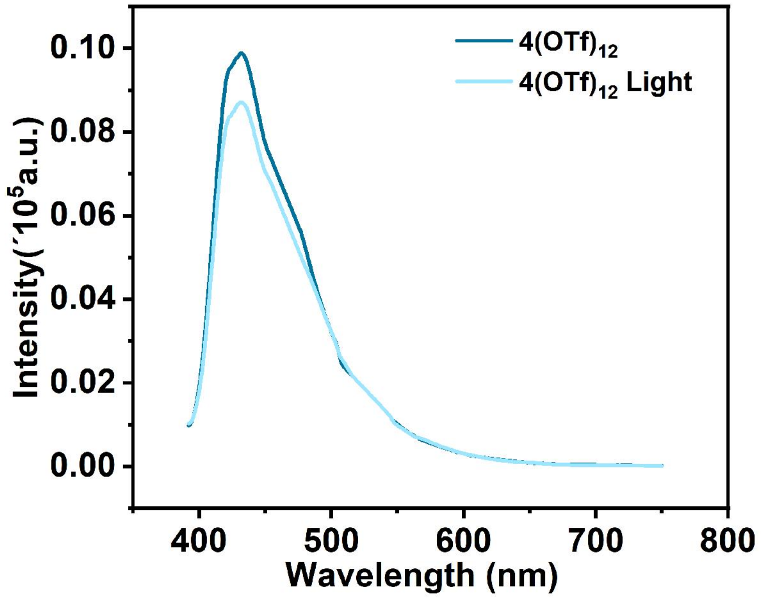

Figure S44. Fluorescence spectra comparison of **4**$(OTf)_{12}$ before and after light irradiation.

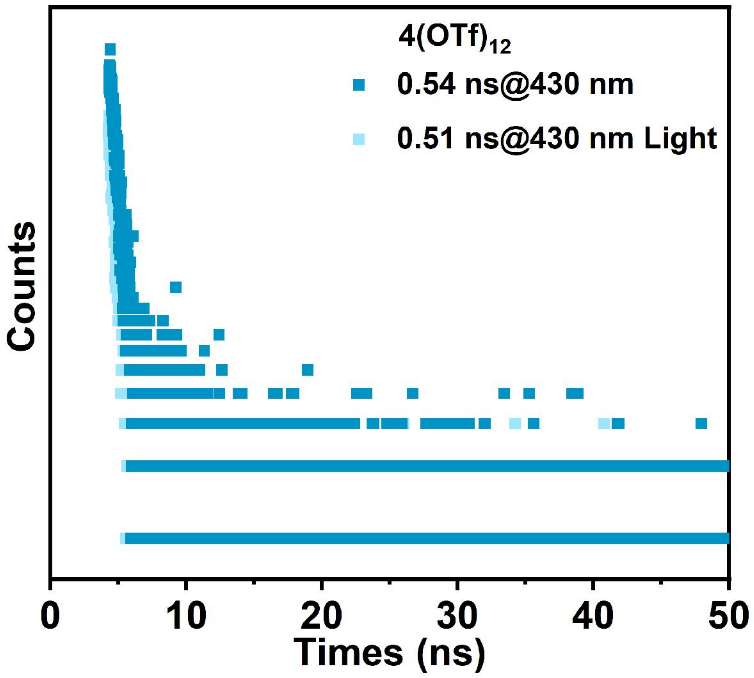

Figure S45. Fluorescence decay curve of **4**$(OTf)_{12}$ before and after light irradiation.

11. X-ray crystallography details and experimental details

Single crystals of **2**(OTf)$_{12}$, **3**(OTf)$_{16}$ and **4**(OTf)$_{12}$ suitable for X-ray diffraction analysis were obtained at room temperature. Their X-ray intensity data were collected at 150 K using a CCD-Bruker SMART APEX system. In these data, disordered solvent molecules that could not be properly restrained were removed using the PLATON Squeeze routine.

In the asymmetric unit of **2**(OTf)$_{12}$, a solvent mask was calculated, and 471 electrons were found in a volume of 1480\%A^3^ in 4 voids per unit cell. This is consistent with the presence of 6[CF_3SO_3] per unit cell, which account for 438 electrons per unit cell.

In the asymmetric unit of **4**(OTf)$_{12}$, a solvent mask was calculated, and 15033 electrons were found in a volume of 57921\%A^3^ in 1 void per unit cell. This is consistent with the presence of 6[CF_3SO_3] per asymmetric unit, which accounts for 15768 electrons per unit cell.

Table S3. Crystal data and structure refinement for snowflake-shaped folded assembly **2**$(OTf)_{12}$.

snowflake-shaped folded assembly **2**$(OTf)_{12}$	
Empirical formula	$C_{372}H_{322}O_{84}N_{30}S_{16}F_{48}Rh_{13}$
Formula weight	9319.37
Temperature/K	150
Crystal system	triclinic
Space group	*P*1
a/Å	20.2428 (17)
b/Å	22.0368 (17)
c/Å	23.2357 (18)
α/°	80.668 (4)
β/°	76.437 (5)
γ/°	76.641 (5)
Volume/Å^3	9739.3 (14)
Z	1
ρ_{calc}g/cm^3	1.589
μ/mm^{-1}	6.034
F(000)	4709.0
Crystal size/mm^3	0.20 × 0.17 × 0.15
Radiation	Cu Kα (λ = 1.54178)
Index ranges	$-23 \le h \le 23$, $-24 \le k \le 25$, $-27 \le l \le 27$
2θ range for data collection/°	2.292 to 63.821
Reflections collected	103573
Independent reflections	31315 [R_{int} = 0.1225, R_{sigma} = 0.1442]
Data/restraints/parameters	31315 / 2495 / 2342
Goodness-of-fit on F^2	1.018
Final R indexes [I>=2σ (I)]	R_1 = 0.1356, wR_2 = 0.2996
Final R indexes [all data]	R_1 = 0.2016, wR_2 = 0.3508
Largest diff. peak/hole / e Å^{-3}	1.044 / -1.061
CCDC number	2482774

Table S4. Crystal data and structure refinement for 8_{18} knot **3**$(OTf)_{16}$.

8_{18} knot **3**$(OTf)_{16}$	
Empirical formula	$C_{482}H_{424}O_{38}N_{24}S_2F_6Rh_{16}$
Formula weight	8985.11
Temperature/K	150
Crystal system	monoclinic
Space group	*P*2
a/Å	53.5860 (18)
b/Å	22.2676 (8)
c/Å	54.8920 (19)
α/°	90
β/°	118.834 (2)
γ/°	90
Volume/Å^3	57379 (4)
Z	4
ρ_{calc}g/cm^3	1.040
μ/mm^{-1}	4.106
F(000)	18376
Crystal size/mm^3	0.20 × 0.17 × 0.15
Radiation	Cu Kα (λ = 1.54178)
Index ranges	$-56 \leq h \leq 59$, $-21 \leq k \leq 24$, $-54 \leq l \leq 61$
2θ range for data collection/°	1.649 to 59.460
Reflections collected	360077
Independent reflections	145651 [R_{int}= 0.1014, R_{sigma}= 0.0682]
Data/restraints/parameters	145651 / 142629 / 10183
Goodness-of-fit on F^2	1.107
Final R indexes [I>=2σ (I)]	R_1 = 0.1031, wR_2 = 0.2071
Final R indexes [all data]	R_1 = 0.1986, wR_2 = 0.2316
Largest diff. peak/hole / e Å^{-3}	0.999 / -0.546
CCDC number	2482775

Table S5. Crystal data and structure refinement for the closed three-link chain **4**$(OTf)_{12}$.

closed three-link chain **4**$(OTf)_{12}$	
Empirical formula	$C_{118}H_{98}O_{30}N_{10}S_6F_{18}Rh_4$
Formula weight	3082.06
Temperature/K	150
Crystal system	trigonal
Space group	*R*3*c*
a/Å	32.8386 (8)
b/Å	32.8386 (8)
c/Å	137.726 (5)
α/°	90
β/°	90
γ/°	120
Volume/Å^3	128622 (8)
Z	36
ρ_{calc}g/cm^3	1.432
μ/mm^{-1}	5.304
F(000)	55944
Crystal size/mm^3	0.20 × 0.17 × 0.15
Radiation	Cu Kα (λ = 1.54178)
Index ranges	$-30 \leq h \leq 32$, $-31 \leq k \leq 30$, $-122 \leq l \leq 136$
2θ range for data collection/°	2.691 to 51.089
Reflections collected	143553
Independent reflections	14801 [R_{int} = 0.1014, R_{sigma} = 0.0682]
Data/restraints/parameters	14801 / 2178 / 1101
Goodness-of-fit on F^2	1.070
Final R indexes [I>=2σ (I)]	R_1 = 0.1545, wR_2 = 0.2935
Final R indexes [all data]	R_1 = 0.2396, wR_2 = 0.3357
Largest diff. peak/hole / e Å^{-3}	0.834 / -0.534
CCDC number	2482776

12. References

1. Gottlieb HE, Kotlyar V, Nudelman A. NMR chemical shifts of common laboratory solvents as trace impurities. *J Org Chem* 1997; **62**: 7512−5.
2. Muller P, Herbst-Irmer R, Spek A *et al*. Crystal structure refinement: a crystallographer's guide to SHELXL (Vol. 8). OUP 2006.
3. Zhang X, Li DS, Wang WX *et al*. A novel NIR-II FL/PA imaging-guided synergistic photothermal-immune therapy: Biomineralizing nanosystems integrated with anti-tumor and bone repair. *Materials Today Bio* 2024; **26**: 101052.
4. Kong XP, Yang YM, Wan GF *et al*. Charge-transfer complex combining reduced cluster with enhanced stability for combined near-infrared ii photothermal therapy. *Adv Healthcare Mater* 2022, **11**: 2102352.
5. Wang C, Dai ZD, Zhang QH *et al*. A bifunctional biomineralized polyoxometalate enabling efficient non-inflammatory NIR-II photothermal tumor therapy. *Chem Eng J*, 2024, **490**: 151601.
6. Wang QC, Zhang TS, Cheng QS *et al*. Combination of efficient red fluorescence and high photothermal conversion in the second near-infrared window from carbon dots through photoinduced sodium-doping approach. *Adv Funct Mater* 2024; **34**: 2402976.
7. Yang RP, Yu XG, Wei CX *et al*. Fe_3O_4 clusters with high photothermal performance for photothermal therapy of tumor. *Chem Eng J* 2025; **504**: 159045.
8. Chen YT, Wen XY, He JX *et al*. Boosting near-infrared photothermal conversion by intermolecular interactions in isomeric cocrystals. *ACS Appl Mater Interfaces* 2022; **14**: 28781−91.
9. Liu XD, Chen B, Wang GG *et al*. Controlled growth of hierarchical Bi_2Se_3/CdSe-Au nanorods with optimized photothermal conversion and demonstrations in photothermal therapy. *Adv Funct Mater* 2021; **31**: 2104424.
10. Jia, ZY, Zhang CL, Wang, XQ *et al*. A borondifluoride-complex-based photothermal agent with an 80% photothermal conversion efficiency for photothermal therapy in the NIR-II window. *Angew Chem Int Ed* 2021; **60**: 22376–84.
11. Zou Y, Zhang HN, Mu QS *et al*. Selective construction of borromean rings and tweezer-like molecular assembly featuring Cp*Rh/Ir clips for near-infrared photothermal conversion. *Chin J Chem* 2023; **41**: 3229−37.
12. Chen T, Zhao Y, Dang LL *et al*. Self-assembly and photothermal conversion of metallaRussian doll and metalla[2]catenanes induced via multiple stacking interactions. *J Am Chem Soc* 2023; **145**: 18036−47.
13. Zhao Y, He Y, Yang JX *et al*. Controllable self-assembly and photothermal conversion of metalla[2]catenanes induced by synergistic effect of free radicals and stacking interactions. *Chin Chem Lett* 2025; **36**: 111460.
14. Dang LL, Zheng J, Tian D *et al*. Highly selective construction of unique cyclic [4]catenanes induced by multiple noncovalent interactions. *Angew Chem Int Ed* 2025; **64**: 202422444.
15. Wang XY, Mu QS, Gao X *et al*. Guest-induced topological transformation and near-infrared photothermal conversion featuring the cp*rh unit via coordination-driven self-assembly. *Sci China Chem* 2025; **68**: 1345−54.
16. Gao X, Cui Z, Shen YR *et al*. Synthesis and near-infrared photothermal conversion of discrete supramolecular topologies featuring half-sandwich [Cp*Rh] units. *J Am Chem Soc* 2021; **143**: 17833−42.
17. Zhang YW, Zhang HN, Wang MX *et al*. Sequence-encoded layered heteroleptic metalla-[2]catenanes for programmable supramolecular function. *Nat Commun* 2026; 17: 1632.
18. Yan Y, Li ZY, Zhang NN *et al*. A *π-π* stacked porous framework for highly efficient second near-infrared

photothermal effects and photo-thermo-electric conversion. *Chem Eng J* 2024; **499**: 156059.
